# ESSM Position Statement “Sexual Wellbeing After Gender Affirming Surgery”

**DOI:** 10.1016/j.esxm.2021.100471

**Published:** 2021-12-28

**Authors:** Müjde Özer, Sahaand Poor Toulabi, Alessandra D. Fisher, Guy T'Sjoen, Marlon E. Buncamper, Stan Monstrey, Marta R. Bizic, Miroslav Djordjevic, Marco Falcone, Nim A. Christopher, Daniel Simon, Luis Capitán, Joz Motmans

**Affiliations:** 1 Department of Plastic, Reconstructive and Hand Surgery, Amsterdam University Medical Center, Amsterdam, The Netherlands; 2 Andrology, Women's Endocrinology, Gender Incongruence Unit, Department of Experimental Clinical and Biomedical Sciences, University of Florence, Florence, Italy; 3 Department of Endocrinology and Center for Sexology and Gender, Ghent University and Ghent University Hospital, Ghent, Belgium; 4 Department of Plastic, Reconstructive and Hand Surgery, Ghent University Hospital, Ghent, Belgium; 5 Department of Plastic, Reconstructive and Hand Surgery, Ghent University and Ghent University Hospital, Ghent, Belgium; 6 Department of Pediatric Urology, University of Belgrade, Belgrade, Serbia; 7 Department of Urology, Città della Salute e della Scienza, University of Turin, Turin, Italy; 8 Department of Urology, St Peter's Andrology Centre and The Institute of Urology, London, UK; 9 Facialteam Surgical Group, HC Marbella International Hospital, Marbella, Málaga, Spain; 10 Center for Sexology and Gender, Ghent University Hospital, Ghent, Belgium

**Keywords:** Transgender, Transsexual, Gender Incongruence, Gender Diverse, Gender Affirming Surgery, Vaginoplasty, Metaidoioplasty, Phalloplasty, Sexual Wellbeing

## Abstract

**Introduction:**

Much has been published on the surgical and functional results following Gender Affirming Surgery (‘GAS’) in trans individuals. Comprehensive results regarding sexual wellbeing following GAS, however, are generally lacking.

**Aim:**

To review the impact of various GAS on sexual wellbeing in treatment seeking trans individuals, and provide a comprehensive list of clinical recommendations regarding the various surgical options of GAS on behalf of the European Society for Sexual Medicine.

**Methods:**

The Medline, Cochrane Library and Embase databases were reviewed on the results of sexual wellbeing after GAS.

**Main Outcomes Measure:**

The task force established consensus statements regarding the somatic and general requirements before GAS and of GAS: orchiectomy-only, vaginoplasty, breast augmentation, vocal feminization surgery, facial feminization surgery, mastectomy, removal of the female sexual organs, metaidoioplasty, and phalloplasty. Outcomes pertaining to sexual wellbeing- sexual satisfaction, sexual relationship, sexual response, sexual activity, enacted sexual script, sexuality, sexual function, genital function, quality of sex life and sexual pleasure- are provided for each statement separately.

**Results:**

The present position paper provides clinicians with statements and recommendations for clinical practice, regarding GAS and their effects on sexual wellbeing in trans individuals. These data, are limited and may not be sufficient to make evidence-based recommendations for every surgical option. Findings regarding sexual wellbeing following GAS were mainly positive. There was no data on sexual wellbeing following orchiectomy-only, vocal feminization surgery, facial feminization surgery or the removal of the female sexual organs. The choice for GAS is dependent on patient preference, anatomy and health status, and the surgeon's skills. Trans individuals may benefit from studies focusing exclusively on the effects of GAS on sexual wellbeing.

**Conclusion:**

The available evidence suggests positive results regarding sexual wellbeing following GAS. We advise more studies that underline the evidence regarding sexual wellbeing following GAS. This position statement may aid both clinicians and patients in decision-making process regarding the choice for GAS. **Özer M, Toulabi SP, Fisher AD, et al. ESSM Position Statement “Sexual Wellbeing After Gender Affirming Surgery”. Sex Med 2022;10:100471**.

## INTRODUCTION

Human sexual behaviour is a complex phenomenon, both for trans and non-trans or cis individuals, orchestrated by the interaction between biological, psychological and social factors. General studies on sexual wellbeing show that having a poor physical health or a chronic illness has a negative impact on sexual wellbeing,^1^^,^^2^ and that issues such as sex frequency, sexual pleasure and sexual satisfaction are strongly positively correlated with mental health.^3^, ^4^, ^5^, ^6^ With trans individuals, having an increased susceptibility to poor mental health outcomes due to a lack of social acceptance and/or access to care,^7^ sexual health outcomes are thought to be equally affected.^8^ Furthermore, for trans individuals who might be undergoing changes in body composition and perception to align these with their gender identity, specific challenges may arise making sexuality a delicate subject to deal with in counselling.^9^ Additionally, data on the significance of sex steroids with respect to sexual functioning and satisfaction in cis individuals^10^, ^11^, ^12^, ^13^, ^14^, ^15^, ^16^, ^17^, ^18^, ^19^, ^20^ brings about the notion that Gender Affirming Medical Interventions (GAMI), such as hormone therapy and surgical interventions, might affect sexual functioning in trans individuals.

### Studies on Sexual Wellbeing

Up to now, studies on sexual wellbeing of trans individuals are scarce or often based on a small population.^21^ Current literature mostly pays attention either at sexuality prior to GAMI,^22^^,^^23^ *or* on the combined effect of hormonal and surgical interventions on sexual wellbeing.^24^, ^25^, ^26^, ^27^, ^28^, ^29^, ^30^, ^31^, ^32^, ^33^, ^34^

Data on sexuality before Gender Affirming Surgery (GAS) from a multicentre prospective study in four European gender identity clinics (Amsterdam, Ghent, Hamburg, Florence, and Oslo) found no difference in frequency of the involvement of the genitalia and appraisal of genital sensation during sexual contact among individuals AMAB and AFAB (Assigned Female at Birth), prior to (GAS).^33^

In a small clinical study, about half of all trans individuals prior to genital surgeries, rated their sexual life as “poor or dissatisfied” or “very poor or very dissatisfied.”^22^ Receiving hormone treatment, experiencing negative feelings, and having a partner, however, were found to relate to better subjective perceptions of sexual quality of life.^22^ Other studies also report on the improved sexual functioning after GAS. The only available prospective study on this matter reported a significant decrease in sexual distress in trans individuals under hormone treatment.^35^ Despite the perceived detrimental effects of hormone treatment on sexual function - especially in individuals AMAB (Assigned Male at Birth)^36^ - sexual distress indeed is reduced after starting hormone treatment,^35^ and sexual wellbeing might significantly improve by minimizing the incongruence between one's body and gender identity.^37^

### Defining Sexual Health in Treatment Seeking Trans Individuals

Although sexual wellbeing is considered as an important aspect of quality of life, and recent studies show considerable improvement of quality of life after GAMI and GAS,^38^, ^39^, ^40^ little information is available on this subject in trans individual after GAS.^22^

This position paper uses ‘sexual wellbeing’ as the core concept of interest. The first written definition of sexual wellbeing originates from 2014 by Byers and Rehman^41^, and Özer et al^42^ modified this definition in the scope of treatment seeking trans individuals in 2021.^42^ Sexual wellbeing in this position statement is a combination of sexuality, enacted sexual script, sexual activities, sexual relations, sexual response cycle, genital function, sexual function, sexual pleasure, sexual satisfaction and quality of sex life.

### Aim

The European Society for Sexual Medicine expressed the need for a position statement on sexual wellbeing after GAS, to supplement the existing World Professional Association for Transgender Health *Standards of Care,* which lacks data on the effects of GAMI *specifically* on sexuality and sexual wellbeing.^43^ This position statement is a continuation on the previous European Society for Sexual Medicine (ESSM) Position Statement on “*hormonal management of adolescent and adult trans people*”.^2^ The adjective ‘trans’ is used here in line with the previous ESSM Position Statement on “*hormonal management of adolescent and adult trans people*”,^2^ to refer to both binary and gender diverse individuals. This position statement therefore does not focus on differences in sexual health outcomes between binary-oriented or non-binary trans individuals, but is aimed at reviewing the available evidence on sexual wellbeing *following* GAS. The position statement wishes to provide clinicians who specialize in trans-related care with recommendations about the impact of various GAS on sexual wellbeing in treatment seeking trans individuals, on behalf of the ESSM.

## Methodology

This position statement aimed at providing results on sexual wellbeing following various gender affirming surgeries, based on the results from a systematic literature review, divided into four main sections: Somatic and General Requirements before GAS, Sexual Wellbeing after GAS (studies who did not specify in gender or surgery when presenting results of sexual wellbeing), Feminizing GAS and Masculinizing GAS, each with the specific surgical procedures and their effects on sexual wellbeing.

The ESSM selected the authors based on their long-standing clinical experience and scientific involvement in specific areas of trans-related healthcare. A multidisciplinary approach was established by involvement of physicians from various specialties, including: endocrinology, oral and maxillofacial surgery, urology, plastic and gender surgery, sociology and sexology.

The search strategy was developed with aid from a research librarian of the Amsterdam University Medical Center. Relevant papers were sourced from the Medline, EMBASE, and Cochrane Library electronic databases from May 2017 until April 2020. Keywords and index terms, including applicable MeSH and Entree terms, were applied to each database. Search terms were generated under two broad headings - ‘gender incongruence’ and ‘sexual wellbeing’ –to create a wide scope on the subject, and were subsequently narrowed down to sexual wellbeing after GAS.

The following MeSH terms were applied to the Medline database: sex reassignment procedures; gender dysphoria; transgender persons; transsexualism; gender incongruence; gender affirming; trans women; trans men; sexual behaviour; coitus, courtship; masturbation; orgasm; dyspareunia; intercourse; copulation; penetration; lubrication; sexual; sensation; pain; arousal; desire; pleasure; satisfaction; dysfunction; wellbeing; relation; behaviour; activity and quality of sex life were applicable for MeSH terminology.

Literature was selected, discussed among the authors and combined with their multidisciplinary knowledge and clinical expertise to establish the statements. The overall quality of evidence of the literature was low, most recommendations of this position statement are therefore low in Level of Evidence. The statements that are strongly recommended are phrased as “should” and suggestions, phrased as “is advised to” or “may”. The statements were formed after consensus of all the authors. Details on the literature search, eligibility and inclusion, data extraction and quality assessment are provided in *Supplement 1.*

An overview of the results on sexual wellbeing following various gender affirming surgeries can be accessed through Table 1-5,7.

**Table 1:** Sexual wellbeing following gender affirming surgery

Study	Design		Sample size	Age range	Sexual Topics	Methods/Tools	Outcomes regarding Sexual Wellbeing
Cohen-Kettenis et al 1997 Sexual Reassignment of Adolescent^63^	Follow-up study	4	9 AMAB 20 AFAB	19–27	Sexual activity, intercourse, orgasm, relationship, masturbation, satisfaction with sex life	Semi-structured interview	Sexually active: n = 13 Orgasm: 77% No partner at time of interview or had never had one: 57% Masturbation: 50% < 1/mo or never, 43% >1/mo; AMAB decrease in frequency, AFAB no change or increase Sexual satisfaction: 71% satisfied, 14% neutral view, 14% were dissatisfied
Jarolim et al 2000 Surgical conversion of genitalia^65^	Outcome meassure	4	52 AMAB 30 AFAB	17–51	Lubrication, orgasm, clitoral activity	Chart review	Vaginoplasty (n = 29): Sexual stimulation leads to production of urethral secretions which served as natural lubrication. Enabled coitus with orgasm Metoidioplasty (n = 28): Preserved erotogenic clitoral activity
Johansson et al 2010 A Five-Year Follow-Up^69^	Follow-up study	4	25 AMAB 17 AFAB	18–60	Relationships, sexual orientation, sex life, sexual impairment	Semi-structured interview, self-developed questionnaire	Sexual impairment after GAS: 5% AMAB Sex life: 70% better, 25% unchanged, 5% worsened Partner relations: 62% better, 30% unchanged, 8% worsened
Kuhn et al 2011 Vaginal prolapse, pelvic floor^70^	Follow-up study	4	52 AMAB 3 AFAB	No data	Sexual activity: satisfaction, frequency	Sheffield prolapse questionnaire	Stable relationship: 71% 75% considered sex life to interfere to some degree with enjoyment of life (better and/or worse) Most were sexually satisfied and had sex on a regular basis
Lief et al 1993 Orgasm in the postoperative^61^	Outcome meassure	4	14 AMAB 9 AFAB	27–63	Frequency of sex (not restricted to coitus, orgasmic capacity, reasons for anorgasmia, sexual satisfaction, sexual preference	Self-developed questionnaire	AMAB Orgasm: 10 anorgasmic, 10/14 Orgasmic before, 8 lost capacity, 4/14 anorgasmic before, 2 gained capacity Reasons for anorgasmia: dissatisfied with vaginal depth or cosmesis Frequency of sex: 75% increase Sexual satisfaction: 9/14 increase in satisfaction AFAB Orgasm: 7/9 Orgasmic, through masturbation, intercourse, and oral sex. 6 anorgasmic before: 4 gained capacities Reasons for anorgasmia: dissatisfied with masculinity level of body Frequency of sex: 100% increase Same people reporting being orgasmic report being sexually satisfied. Sexual satisfaction: 6/9 increase in satisfaction
Lobato et al 2006 Follow-Up of Sex Reassignment^68^	Follow-up study	4	18 AMAB 1 AFAB	18–47	Satisfaction with sexual experience, partnerships, and relationship with family members, sexually active, frequency of sex, pleasurability of sex.	Self-developed questionnaire	Sexually active: 95% More frequent sex: 64.7% Pleasurability of sex: 83.3% Partnership Initiating and maintaining relationship easier post SRS: 64.7% Relationship pre-op: 52.6% Relationship post-op: 73.7% Satisfaction with sexual experience poor or very poor post-op: 11.2% Improvement of sexual experience post-op: 83.3%
Lothstein et al 1980 The postsurgical transsexual^71^	Group comparison / Follow-up study	4	8 AMAB 6 AFAB	21–42	Sexual adjustment and functioning, improvement sex life, sexual activity, number of sexual partners, orgasm, sexual behaviour, partnering	Self-developed questionnaire	AMAB Improvement sex life 67% Sexual activity & number of sexual partners: no increase Orgasm: 2 ejaculatory sensations Sexual behaviour: more flexible and spontaneous (engaging more frequently in oral, anal, and vaginal intercourse) Partnering: tended to seek out new partners, 64% no relationship AFAB Improvement sex life 67% Sexual activity & number of sexual partners: no increase Partnering: kept the same partners, 64% no relationship
Rakic et al 1996 The outcome of sex reassignment^66^	Follow-up study	4	22 AMAB 10 AFAB	19–47	Orgasm, number of sex partners, sexual activity, satisfaction with relationships	Self-developed questionnaire: 'Adjustment to Sex Reassignment Surgery'	Relationship satisfaction: 87% Sexual partner: AMAB 23%, AFAB 80% Several sexual partners: AMAB 50% Orgasm: 50%
Selvaggi et al 2007 Genital sensitivity after sex reassignment^33^	Outcome meassure	4	30 AMAB 27 AFAB	No data	Orgasm, sensitivity	Interview and physical examination: Semmes-Weinstein, vibration tests (biothesiometer)	The reconstructed genitalia obtain tactile and erogenous sensitivity Orgasm: AMAB 85%, AFAB 100%
Smith et al 2001 Adolescents With Gender Identity Disorder^64^	Group comparison / Follow-up study	4	7 AMAB 13 AFAB	15–19	Orgasm, relationship status, sexual orientation, intercourse, sexual functioning	Self-developed questionnaire, Utrecht Gender Dysphoria Scale (UGS), Body Image Scale (BIS)	Sexual partner: 10 patients Satisfaction with sex life: 1 FM was dissatisfied (could not have intercourse with a “normal” penis) Several AFAB: difficult to live without a penis know their potential sexual partner well. Masturbation: AMAB –>decrease in masturbation frequency, AFAB–> increase or no change. Orgasms: 69% Sexual orientation: stayed compatible
Sorensen 1981 A follow-up study^60^	Outcome meassure	4	8 AFAB	30–60	Intercourse, sexual satisfaction, masturbation, orgasm, sensitivity, ability to perform intercourse, importance of sexual activity, pain	Structured interview	Sexual partner: all patients Sexual satisfaction: only from clitoris (all); 5 satisfactory, 3 unsatisfactory Masturbation: all, >2 a wk Orgasm: all Intercourse: 2 with phalloplasty with rib, but with pain, 6 with dildo Importance of sexual activity: essential in a life with a partner to all Pain: during intercourse in the 2 patients with phalloplasty from rib
Tsoi et al 1993 Follow-up study of^67^	Outcome meassure	4	45 AMAB 36 AFAB	20–36	Satisfaction with: sexual activities, organ functioning, sex status, sex organ	Semi-structured interview, self-developed questionnaire	Good or satisfactory sexual activity: MtF 64% vs FtM 61% Good or satisfactory sex organ function: MtF 91% vs FtM 39% Good or satisfactory sex status: MtF 95% vs FtM 81% Good or satisfactory sex organ: MtF 91% vs FtM 39%
van de Grift et al 2017 A longitudinal study^62^	Follow-up study	4	21 AFAB	Mean 40.1 y	Sexual activity (type of activity), sexual preference and change in preference, partnering, sexual satisfaction	Self-developed questionnaire, chart review, Body Image Scale (BIS)	Female partner 63,2% Male partner 10,5% Single 26,3% Sexual function: metoidioplasty higher sexual satisfaction Postop sexual activity: more masturbation and sexual activity, genitals more freq used (31% vs 78%) More pleasure, confidence, passive role Changed sexual orientation (“exclusively to men” to “primarily to women”). Grade for sex life: 5.5 of 10 (SD 2.6) à “impossibility to penetrate/no erection prosthesis”, “not sexually active”, “penile size/sensation” and “partner issues”
Wierckx et al 2011 Quality of life and^26^	Follow-up study	4	49 AFAB	22–54	Relationship status, sexual preference, sexual activities (frequency, type of activity, pain), sexual wellbeing, masturbation, sexual satisfaction, orgasm, arousal	Self-developed questionnaire	Treatment phase differed within the group Relationship 63,3% Attracted to females 85,7% Partner: heterosexual woman 77,4% Frequency of sex: Never 22,2% 1-2/month 48,1% Several times a week 29,6% Sex satisfaction: (very satisfied) 64,2% Erection prosthesis (n = 32) Frequency of masturbation: Less than monthly- daily Aroused easily: majority Orgasm through masturbation: majority Orgasm by intercourse: majority Change in orgasmic feeling: 58,3%

## Somatic and General Requirements Before GAS

*Statement #1 The gender surgeon should be aware of the effects of smoking and BMI when considering (genital) GAS. (Level I Grade A)*

*Statement #2 The gender surgeon should engage in shared decision making and counsel the patient on expectation management, including expected sexual outcomes, prior to GAS (Level II Grade D)*

*Statement #3 The gender surgeon is advised to collaborate with sexologists and pelvic floor physical therapists, trained on trans related health care, if available (Level IV Grade D)*

### Evidence

The surgeon and anaesthesiologist are tasked with assessing the general health status, perioperative risk, and contraindications as per the American Surgical Association physical status classification system, for individuals requesting surgery.^44^^,^^45^ Patients should be advised on smoking cessation and ideal weight for surgery, that is, a BMI between 18–30 kg/m^2^, prior to genital GAS.^46^, ^47^, ^48^ The eventual decision for surgery in patients outside of the ideal BMI range falls upon the surgical and anaesthesiology team and should not be considered a hard Contra-Indication.

Hormone therapy may adversely affect fertility in both AFAB and AMAB individuals,^49^ while GAS may terminate potential for reproduction. Fertility preservation that is, cryopreservation of semen or oocytes, embryos or ovarian tissue- may aid in facilitating future parenting options.^50^

The choice between various surgical techniques for GAS is dependent on patient preference, patient anatomy and health status, and the surgeon's skillset. Choices are increasingly being made through shared decision-making.^51^ The surgeon should inform the patient on the techniques available, their advantages and disadvantages, limitations with producing ‘ideal’ results and possible risks and complications.^52^, ^53^, ^54^ How the surgeon presents surgical options, risks and benefits is of great importance. The surgeon should preferably present photos of their previous work and provide data on their complication rate.^52^ Little has been published on postsurgical regret in regard to functional outcomes and complication rate. Lawrence, however, found that less complications and better functional results after vaginoplasty were associated with less postsurgical regret.^55^^,^^56^

## Sexual Wellbeing After Gender Affirming Surgery

Gender Affirming Surgery (GAS) is an umbrella term for a variety of surgical procedures.^57^ It is important to note that trans individuals may or may not adhere to a standard linear progression from hormone treatment to surgical procedures.^58^ Sexual motivations may influence some individuals to prefer surgical interventions without prior hormone treatment, or opt out of some surgical procedures.^59^

The outcomes on sexual wellbeing following GAS are found in Table 1*.* Fourteen studies reported on general sexual wellbeing following GAS, without specifying what kind of procedure was performed or how the participants identified gender-wise, mainly focusing on sexual activities, erogenous sensation, orgasm and sexual satisfaction. Frequency of sexual activities increased after both hormonal and surgical treatment.^26^^,^^60^, ^61^, ^62^ Frequency of masturbation, however, was decreased in AMAB individuals and remained unchanged or increased in AFAB individuals.^63^^,^^64^ Every patient experienced postsurgical tactile erogenous, to some extent.^33^ Every AFAB individual and 85% of AMAB individuals were able to reach orgasm,^33^ either through masturbation or intercourse.^26^^,^^60^^,^^61^^,^^63^^,^^65^^,^^66^ Orgasm after GAS was experienced more frequently by both AMAB and AFAB individuals,^66^ less frequently by AMAB individuals,^61^ than by AFAB individuals.^61^

Most AMAB individuals were satisfied with GAS, reporting sexual satisfaction with the possibility of penetrative sex^61^^,^^67^ and being partnered.^63^ Initiating and maintaining intimate relationships became easier postoperatively.^66^^,^^68^ Limited sensitivity and absence of erectile function after phalloplasty decreased sexual satisfaction in AFAB individuals.^67^ Phalloplasty was not found to be a critical factor in reaching orgasm or sexual satisfaction,^61^ difficulties in engaging in new sexual contacts, however, may have been a factor preoperatively.^63^^,^^64^ The strongest motivation to pursue penile surgery was confirmation of one's identity.^62^ Postsurgical aesthetics and functionality were satisfactory, including the ability to void while standing.^65^ Disappointment following GAS resulted because of a decrease in sex drive, not being partnered or having non-functional genitalia.^69^

Advice regarding postsurgical care and follow-up were provided by two studies. Kuhn et al^70^ concluded that pelvic floor symptoms involving the bladder, bowel, and sexual function may occur in AMAB individuals. Lothstein et al^71^ advised counselling and psychotherapy prior to surgery and continued follow-up after completing GAS to improve sexual wellbeing.

## Surgical Procedures for Feminizing GAS

This section addresses different types of feminizing GAS, with their respective results regarding sexual wellbeing.

### Orchiectomy-Only

*Statement #4 The gender surgeon is advised to consider orchiectomy-only as a viable surgical option for trans individuals AMAB (Level IV Grade D)*

*Statement #5 The gender surgeon should counsel the trans individual AMAB on the expected effects on sexual wellbeing prior to orchiectomy (Level II Grade A)*

#### Evidence

Indications include patient preference- in not opting for vaginoplasty- and failing at meeting somatic requirements for anti-androgen use or vaginoplasty (see: *somatic requirements before GAS*). Orchiectomy does not provide surgical consequences for future vaginoplasty, therefore can also be performed while waiting for a future vaginoplasty. Testosterone levels below 20 ng/dL (0.7 nmol/L) have been reported in patients following orchiectomy,^72^ patients should be counselled on possible adverse effects of low testosterone levels on sexual wellbeing.^73^

#### Sexual Wellbeing After Orchiectomy

We found no studies on sexual wellbeing in trans individuals AMAB after orchiectomy-only.

### Vaginoplasty

*Statement #6 The gender surgeon should provide trans individuals AMAB the penile-inversion technique as the vaginoplasty of choice (Level IV Grade C)*

*Statement #7 The gender surgeon should be capable to offer alternatives to the penile-inversion technique, in trans individuals AMAB, like: skin grafts or bowel segments to create adequate vaginal depth in cases of penoscrotal hypoplasia and inadequate penile skin length (Level IV Grade C)*

*Statement #8 The gender surgeon is advised to counsel on vulvoplasty (or zero-depth vaginoplasty) when this is recommended by health care professionals or requested by the patient, to reduce possible future regret in trans individuals AMAB (Level IV Grade D)*

*Statement #9 The gender surgeon should counsel trans individuals AMAB on expected sexual outcomes, pelvic floor symptoms and possible complications for any kind of vaginoplasty (Level # IV Grade D)*

#### Evidence

Vaginoplasty-comprised of vulvoplasty, penectomy, orchiectomy and vaginal canal creation-aims at obtaining an aesthetically pleasing and functional genital complex, vulva and neo-vagina, with adequate depth.^74^ The vaginal canal is created between the rectum and bladder, and lined with penile skin- optionally modified with skin grafts, urethral mucosa or scrotal flaps-skin grafts-only,^75^ bowel segments or peritoneum.^76^ The clitoris is formed by the dorsocentral part of the glans penis, the clitoral hood is formed either from the prepuce or with penile skin. Urethral grafts may aid in vaginal lubrication and sensitivity.^77^ The penile-inversion vaginoplasty is currently considered the gold standard.^78^ Studies show that penile-inversion vaginoplasty is associated with satisfaction with aesthetics and function.^79^^,^^80^ (See below 5.3.2).

Indications for vulvoplasty (or zero-depth vaginoplasty) include patient preference or extensive morbidity, for example, a history of rectal fistula.^81^ Counselling is strongly recommended to minimize the risk of future regret.^82^

Sexologists and pelvic floor physical therapists may counsel patients on dilation and aid in reducing voiding difficulties, which are not related to meatal stenosis. Consultation should preferably be commenced prior to surgery, and continued postoperatively.^83^ The sexologist may address issues regarding changing sexual function, for example; in, phantom pains, sexual stimulation and arousal.^84^, ^85^, ^86^ Possible complications of vaginoplasty are perforations and fistulae, haemorrhage and possible future secondary corrections. Secondary corrections are dependent on patient preference and may include resection of residual spongiosum, labiaplasty, clitoral repositioning, correction of the meatus or introitus and vaginal depth augmentation.

#### Sexual Wellbeing After Vaginoplasty

Sixty-one studies reported outcomes on sexual wellbeing following vaginoplasty, available in Table 2.^55^^,^^79^, ^80^, ^81^^,^^84^^,^^85^^,^^87^^,^ ^88^^,^ ^89^^,^ ^90^^,^ ^91^^,^ ^92^^,^ ^93^^,^ ^94^^,^ ^95^^,^ ^96^^,^ ^97^^,^ ^98^^,^ ^99^^,^ ^100^^,^ ^101^^,^ ^102^^,^ ^103^^,^ ^104^^,^ ^105^^,^ ^106^^,^ ^107^^,^ ^108^^,^ ^109^^,^ ^110^^,^ ^111^^,^ ^112^^,^ ^113^^,^ ^114^^,^ ^115^^,^ ^116^^,^ ^117^^,^ ^118^^,^ ^119^^,^ ^120^^,^ ^121^^,^ ^122^^,^ ^123^^,^ ^124^^,^ 125, 126, 127, 128, 129, 130, 131, 132, 133, 134, 135, 136, 137, 138, 139, 140, 141 Postoperative genital sensitivity- defined as clitoral sensation, orgasmic sensation and genital sensation-was conserved in almost every patient.^79^^,^^87^^,^^90^^,^^95^^,^^98^^,^^99^^,^^101^^,^^102^^,^^117^^,^^118^^,^^120^^,^^126^ Subjective arousal and desire were similarly experienced by a majority (79,1%) of postsurgical women.^79^^,^^90^^,^^108^^,^^123^^,^^135^^,^^142^

**Table 2:** Sexual wellbeing following vaginoplasty

Study	Design	LoE	Sample size	Age range	Sexual topics	Methods/Tools	Outcomes regarding Sexual Wellbeing
Amend et al 2013, Surgical reconstruction^87^	Outcome measures	3	13 AMAB	20–54	Intercourse, satisfaction, neo-clitoral sensation, vaginal depth, orgasm	Self-developed structured interview	23 (96%) were satisfied with neo-clitoral sensitivity, which led to orgasm. Neo-clitoral sensation was excellent in 18, good in 5, and unsatisfactory in 1. Eight (33 %) had engaged in intercourse, without the need for lubrication. None experienced intravaginal hair growth or loss of vaginal capacity.
Blanchard et al 1983, Vaginoplasty outcome^88^	Outcome measures	3	22 AMAB		Orgasm, intercourse, self-reported depth adequacy, pain/discomfort during sex, discomfort after sex, frequency of sex, sexual orientation	Structured interview, pelvic exam	19 (86,4%) had intercourse at least once: 8 experienced no pain, 2 did always, 5 did initially or after a period of sexual inactivity, 4 did slightly. 3 experienced discomforts after sex
Bouman et al 1988, Sex reassignment^89^	Outcome measures	4	76 AMAB	No data	Sexual intercourse, satisfaction during sex, neo-vaginal dimensions,	Chart review	3 had complaints due to small vaginal diameter, one was unable to perform receptive vaginal sex. 30 had intercourse with men, 11 with men, 15 had not.
Bouman et al 2016, Patient-Reported^90^	Follow-up study	4	31 AMAB	18–45	Vaginal intercourse, neo-vaginal dimensions, sexual arousal, sexual feelings, orgasm, desire, lubrication, satisfaction	Female Sexual Function Index, Female Genital Self-Image Scale, Short Questionnaire for Self-Evaluation of Vaginoplasty, Amsterdam Hyperactive Pelvic Floor Scale—Women	21 were sexually active, 16 had sex more than once. Every participant experienced sexual arousal. 84 % could reach orgasm, 4% could not, and 12% had not tried.
Brotto et al 2005, Psychophysiological and^91^	Prospective cross-sectional study	3	15 AMAB	21–65	Thoughts/desire, Frequency of sexual activity, receptivity/initiation, relationship satisfaction, problems affecting sexual function, sexual arousal, non-genital physical arousal, genital arousal, pleasure from direct genital stimulation, orgasm (eg, clitoral stimulation, intercourse, vibrator use, fantasy), satisfaction with orgasmic function, dissatisfaction or distress, effects of erotic stimuli, objective arousal: using a vaginal pulse amplitude	Self-developed questionnaire during Vaginal pulse amplitude (VPA), Brief Index of Sexual Functioning for Women (BISF-W); Detailed Assessment of Sexual Arousal (DASA);	4 were sexually active, 6 were able to achieve orgasm. 10 were satisfied with their orgasmic function, 3 (20%) dissatisfied or distressed.
Buncamper et al 2015, Aesthetic and^79^	Retrospective cross- sectional survey	4	49 AMAB	29–53	Sexually activity, desire, arousal, lubrication, orgasm, satisfaction, comfort, sexual intercourse, neo-vaginal dimensions, sexual feelings	Female Sexual Function Index (FSFI), Amsterdam Hyperactive Pelvic Floor Scale-Women (AHPFS-W), Female Genital Self-Imaging Scale, short questionnaire for self-evaluation of vaginoplasty	36 were sexually active, 27 had attempted intercourse (3 of those tried but were unable). 83.7% had reached orgasm, 10.2% had not, 6.1% had not tried. Orgasmic sensation was equal in 22.4%, less in 28.6%, more in 46.9% and was missing in 2%, compared to prior to vaginoplasty. Provoked vulvodynia was scored with a mean of 1.33 (SD 0.75; 5-point scale from never - very often). Self-reported sexual arousal was present in 44.
Buncamper et al 2017, Penile Inversion^92^	Group comparison	4	100 AMAB	18–68	Sexually activity, desire, arousal, lubrication, orgasm, satisfaction, comfort, sexual intercourse, neo-vaginal dimensions, sexual feelings	Female Sexual Function Index (FSFI), Female Genital Self-Imaging Scale	42 had been sexually active in the last 4 weeks. Median score for vaginal functionality: 8 (range 2–10; 1–10 scale, 10 being better; n = 45).
Cardoso da Silva. et al 2016, WHOQOL-100^93^	Follow-up study	4	47 AMAB	16–54	Marital status, sexual activity	WHOQOL-100	5 were in a stable relationship, 42 were not.
Cocci et al 2019, Male-to-female^138^	Outcome measures	4	94 AMAB	M 29.5 y	Intercourse, erogenous sensitivity	Not specified	81 (86.1%) had intercourse. Erogenous sensitivity during dilatation, intercourse or masturbation was present in 78 (82.9%).
Collyer et al 2002, Patient satisfaction^94^	Outcome measures	4	57 AMAB	21–35	Orgasm, sexual satisfaction	Self-developed questionnaire	34 patients were more sexually satisfied post-surgery; 17 patients had no change; 4 patients were less satisfied.
di Summa et al 2019, Colic-based^139^	Outcome measures	4	43 AMAB	22–69	Satisfaction with the appearance/dimensions of the genitals, satisfaction with genital function (ing), orgasm (clitoral, vaginal or both), difficulties achieving orgasm, dyspareunia	Retrospective chart review, custom questionnaire	*Of* n = 28 10 (35.7%) was very satisfied with sexual functioning, 14 (50%) satisfied, 4 (14.3%) unsatisfied, none very unsatisfied. 25 (89.3%) was satisfied or very satisfied with vulvar appearance, 3 (10.7%) unsatisfied, none very unsatisfied.
Djordjevic et al 2011, Rectosigmoid vaginoplasty^84^	Outcome measures	4	27 AMAB 59 women	18–57	Vaginal dimensions, mucous production, sexual satisfaction, sexual activity, time till first intercourse, pain	FSFI, interview	Sexual function was rated satisfactory in 21, 6 were unsatisfied. 73 individuals of the entire cohort were sexually active, separate results not provided. 27 experienced temporary mild bleeding and dyspareunia.
Eldh et al. 1993, Construction of^95^	Outcome measures	4	20 AMAB	No data	Orgasm, sexual function, clitoral sensation, intercourse	Chart review	20 could reach orgasm through masturbation or intercourse. 19 (95%) were pleased with their neo-clitoral sensation, 1 (5%) had no sensitivity.
Freundt et al 1993, A modified^96^	Outcome measures	4	23 AMAB	16–52	Sexual relations, vaginal function, sexual satisfaction, intercourse	Structured interview, pelvic examination	5 had regular intercourse, 4 women occasionally, 1 did not. Sexual satisfaction was rated good by 2, satisfactory by 2, doubtful by 5, and unsatisfactory by 1. 2 were satisfied with sex life (20%), 4 were neutral, and 4 dissatisfied.
Giraldo et al. 2004, Corona glans^97^	Outcome measures	4	16 AMAB,	20–41	Orgasm	Chart review	16 were able to achieve orgasm.
Goddard et al. 2007, Feminizing genitoplasty^98^	Outcome measures	4	233 AFAB, 70 (follow-up)	19–76	Clitoral sensation, sexual arousal, vaginal dimensions, intercourse, orgasm	Telephone questionnaire	Of 70 with follow-up, 64 had a neo-clitoris and 62 a vaginal canal: 14 had regular intercourse. 31 could reach clitoral orgasm. Of 183 with neo-clitoral formation: neo-clitoris was sensitive in 158, insensitive in 5, NA in 20. 4 experienced painful or uncomfortable clitoral sensations.
Hess et al 2016, Modified preparation^99^	Follow-up study	4	96 AMAB	19–62	Neo-clitoral sensation, orgasm	Semi-quantitative grading of neo-clitoral sensitivity	Assessment of sensitivity by brushing over the clitoris with a brush, and pallesthesia by placing a 64 Hz tuning fork on the clitoris. A semi quantitative scoring system was formed by accumulating both: grade 0, no tactile sensation and complete pallanesthesia; grade 1, reduced pallesthesia and no tactile sensation; grade 2, intact pallesthesia and reduced tactile sensation; grade 3, complete pallesthesia and tactile sensation. n = 79: 11 had grade 1, 12 grade 2, and 56 grade 3. After second-stage (cosmetic corrections). n = 73: 59 could reach orgasm, 7 could not despite trying, 7 (9.6%) had not tried.
Hess et al 2018, Sexuality after^140^	Follow-up study	4	119 AMAB	16–68	Sexual orientation, intercourse, frequency of sex, orgasm, orgasm frequency and sensation, satisfaction with clitoral sensitivity, satisfaction with the appearance/dimensions of the genitals, satisfaction with sex life, pleasurability of sex, sexual arousal	Unspecified questionnaire	33.7% were heterosexual, 37.6% lesbian, and 22.8% bisexual. 67 (56.3%) did not have regular intercourse. Of those who had sexual intercourse, 55.8% rated orgasm more intense following GAS, 20.8% who felt no difference. 73.9% were satisfied with neo-clitoral sensitivity, and 67.1% with vaginal depth. Of n = 88: sexual activity was always pleasurable for 31 (35.2%), sometimes pleasurable for 44 (50.0%), and never pleasurable for 13 (14.8%).
Imbimbo et al 2009, A report from^100^	Outcome measures	4	163 AMAB	21–59	Sexual activity, type of sexual activity, orgasm, masturbation, Satisfaction with sexual life, vaginal dimensions	Telephone questionnaire	124 were sexually active: 60 had receptive vaginal sex, 75 receptive anal sex. 32 had masturbated. Satisfaction with sexual life post-surgery was improved in 75% and worsened or unchanged in 25%.
Jarolim et al 2009, Gender reassignment^101^	Outcome measures	4	129 AMAB	18–54	Neo-clitoral erogenous sensation, orgasm, lubrication	Chart review	Of n = 98: 92 (94%) had erogenous sensitivity of the neo-clitoris had. 64 (65%) reached orgasm 3 mo., some with urethral secretions, which provided lubrication.
Kanhai et al 2016, Sensate vagina^102^	Outcome measures	4	50 AMAB	19–65	Erogenous sensation in both clitoral pedicles	Chart review	46 (92%) experienced erogenous sensitivity and 41 (82 %) sexual sensations in the clitoris. 44 (88%) experienced erogenous sensitivity and 31 (62 %) sexual sensitivity of the sensate pedicled spot.
Jiang et al 2018, Does depth matter^81^	Outcome measures	4	30 AMAB	28–74	Relationship status, orgasm, sexual activity.	Case-series	Of n = 14: 4 (29%) could achieve orgasm, 3 (21%) could not. 7 (50%) were not sexually active. Of n = 30: 17 (57%) were married or in a stable relationship, 13 (43%) were not.
Karim et al 1991, The importance of^103^	Outcome measures	4	13 AMAB	23–51	Swelling and narrowing of vagina during sexual arousal	Chart review	10 experienced vaginal swelling and narrowing during sexual arousal, none did after removal of the corpora spongiosa and cavernosa, none did after removal of the tissue.
Kim et al 2003, Long-term results^104^	Outcome measures	4	28 AMAB	22–50	Vaginal dimensions, sexual intercourse, lubricant use, pain during intercourse, orgasm, vaginal bleeding during intercourse	Cross-sectional study	22 (78.6%) had intercourse: 1 experienced abdominal pain and 2 vaginal bleeding during intercourse, and 19 could reach orgasm during intercourse.
Kim et al 2017, Is Rectosigmoid^105^	Outcome measures	4	44 AMAB 29 vaginal agenesis 8 female pseudohermaphroditis 3 gynaecologic malignancies after radical pelvic surgery	23–47	Sexual intercourse, orgasm	Chart review	79 (94%) had intercourse: 72 experienced orgasms, 2 had mild intermittent abdominal pain, 6 long-lasting abdominal pain, and 6 a small amount of vaginal bleeding after intercourse.
Krege et al 2001, Male-to-female^106^	Follow-up study	4	66 AMAB	20–57	Sexual intercourse, problems during intercourse, recurrent bleeding after intercourse, clitoral orgasm, vaginal dimensions	Self-developed questionnaire	n = 31 with follow-up: 27 (87%) could reach clitoral orgasm, 18 (58.1%) had intercourse, 8 (25.8%) had problems during intercourse (1 swelling of remnants of the corpus spongiosum; 1 problem intravaginal suture line; 2 pains during intercourse; 1 recurrent bleeding after).
Lawrence et al 2003, Factors associated^55^	Follow-up study	4	232 AMAB	19–72	Vaginal dimensions, vaginal lubrication, vaginal discharge, sensation to touch at the vaginal opening, sensation to touch deep in the vagina, vaginal pain with penetration, vaginal erotic sensation, clitoral touch sensation, clitoral erotic sensation, clitoral pain, sexual attraction, sexual experience, arousal	Self-developed questionnaire	The number of surgical complications was negatively correlated, and functional results were positively correlated with the absence of regret regarding vaginoplasty. The amount of psychotherapy and the number of complications were negatively, and functional results were positively correlated with happiness with the results of vaginoplasty.
Lawrence et al 2005, Sexuality before^107^	Follow-up study	4	232 AMAB	19–72	Sexual orientation, number of sexual partners, frequency of sexual activity, stable partnered relationships, sexual arousal to cross-dressing or cross-gender fantasy, frequency and characteristics of orgasm after GAS	Self-developed questionnaire	Of n = 226: 76% had postsurgical sexual experiences (28% had mostly female partners, 38% mostly male, 25 % bisexual). Of n = 226: 214 (95%) were sexually active prior to surgery, 12 (5.3%) were not. 72% had mostly female partners, 8% mostly male, 15% bisexual. Of n = 227, 217 (95.6%) had masturbated: 82 (36%) almost always orgasm during masturbation, 27 (12%) > half the time, 33 (15%) did < half the time, 34 (15%) rarely, 41 (18%) never, and 10 (4%) NA. Of n = 217: orgasm prior to and after surgery was almost identical for 4 (2%), very similar for 19 (9%), somewhat similar for 53 (24%), slightly similar for 52 (24%), entirely different for 57 (26%), NA for 32 (15%) . Of n = 218: orgasm after surgery was much more pleasurable for 65 (30%), somewhat more pleasurable for 45 (21%), about as pleasurable for 35 (16%), somewhat less pleasurable for 35 (16%), much less pleasurable for 8 (2%), NA for 30 (14%). Of n = 217: 52 (24%) almost always released fluids during orgasm, 22 did > half of the time for 22 (10%), 17 (8%) did < half of the time, 29 (13%) did rarely, 40 (18%) never, and 57 (26%) NA.
Lawrence et al 2006, Patient-reported complications^85^	Follow-up study	4	232 AMAB	19–72	Vaginal dimensions, vaginal lubrication, vaginal discharge, sensation to touch at the vaginal opening, sensation to touch deep in the vagina, vaginal pain with penetration, vaginal erotic sensation, clitoral touch sensation, clitoral erotic sensation, clitoral pain, sexual attraction, sexual experience, arousal, frequency of orgasm	Self-developed questionnaire	Mean rating on 0–10 scale, 10 being better, were: 7.8 (SD 2.4) for overall happiness with genital sexual function after GAS; 4.4 (SD 2.8) for vaginal lubrication; 7.1 (SD 2.4) for mean rating for pain with vaginal penetration. Frequency of achieving orgasm was significantly associated with overall happiness with sexual function. Individuals who could never orgasm were significantly less happy with their sexual function than others.
LeBreton et al 2017, Genital Sensory^108^	Outcome measures	3	28 AMAB	25–60	Genital sensitivity, overall satisfaction (patient's satisfaction with the appearance of their genitals, sexual functioning, and clitoral sensitivity), frequency of sexual activities (masturbation, mutual masturbation, vaginal intercourse, anal stimulation, anal intercourse, and oro-genital stimulation) orgasm frequency with each of these activities.	Genital sensitivity: Semmes-Weinstein monofilaments (light touch), vulvalgesiometer (pressure), vibralgic 4 device (vibration), questionnaire developed by Lothstein and Shinar, self-developed questionnaire, Derogatis Fantasy Scale.	Subjective clitoral sensation was not statistically significantly correlated with sexual satisfaction. Detection thresholds for light touch showed the highest sensitivity on the neck, followed by the anus, abdomen, clitoris, labia minora and then the vaginal opening. Detection thresholds for pressure showed the highest sensitivity on the neck, followed by the clitoris, anus, abdomen, labia minora and the vaginal opening. Detection thresholds for vibration showed the highest sensitivity on the clitoris; followed by the labia minora; the neck; the abdomen; and the vaginal opening and anus. *Frequencies of sexual activities ranged from: 0 = N.A.; 1 = < 1/y, 2 =< 1/mo; 3= 1/mo; 4 = 2/mo; 5 = 1/wk; 6 = several times/week; 7 = 1/day; 8 = >1/d*. 20 (80%) had experienced orgasm. Mean frequencies of achieving orgasm prior to and after having GAS, respectively, were 0.90 (SD 1.38) and 0.56 (SD 1.36) for masturbation, 0.50 (SD 1.47) and -0.24 (SD 1.27) for mutual masturbation, 1.00 (SD 1 .29) and -0.12 (SD 1.09) for vaginal intercourse (penetrative and receptive, respectively), -1.18 (SD 1.30) and -0.16 (SD 1.52) for receptive oral sex, -0.14 (SD 1.36) and -0.36 (SD 1.15), and -0.05 (SD 1.47) and 0.08 (SD 1.04). Prior to GAS: mean frequency of receptive anal sex was 3.20 (SD 2.63), 2.40 (SD 2.71) for insertive vaginal sex, 2.32 (SD 2.75) for receptive oral sex, and 3.20 (SD 2.48) for receptive anal stimulation. Following GAS: mean frequency of receptive vaginal sex was 3.44 (SD 2.49), 2.04 (SD 2.54) for receptive anal sex, 3.48 (SD 2.45) for receptive oral sex, and 2.48 (SD 2.69) for receptive anal stimulation. The difference between pre- and postoperative frequency of receptive oral sex was statistically significant.
Lindemalm et al 1986, Long-term^109^	Evaluation of GAS	4	13 AMAB	27–62	Sexual adjustment, sexual function, libido, sexual activity, orgasm, partner relations	Semi structured interview, chart review	Of n = 12: 11 (92%) were sexually active prior to GAS, 1 (8%) was not. Following GAS, 10 (77%) were sexually active. Orgasm prior to GAS: 9 (69.2%) could, 1 (7.7%) could not, unclear for 3 (23.1%). Orgasm after GAS: 6 (46.2%) could (2 with ejaculation), 6 (46.2%) could not, unclear for 1 (7.6%). Strength of libido prior to GAS was low for 5, high for 6, NA for 2. Following GAS: low for 7 (1 of which previously high; 2 NA), moderate for 1 (previously low), high for 5.
Lindemalm et al 1987, Prognostic factors^110^	Evaluation of GAS	4	13 AMAB	27–62	Sexual adjustment, libido, sexual activity with partner, number of partners, orgasm, object choice, partner relations	Retrospective rating of interview	The following outcomes prior to GAS were associated with fair or good overall sexual adjustment after GAS: high sexual activity with a partner, strong libido, intercourse with women, and bisexual experience. High frequency of masturbation was not associated with good adjustment.
Manrique et al 2018, Gender-Confirmation ^111^	Evaluation of rectosigmoid vaginoplasty	4	15 AMAB	18–32	Sexual function	Retrospective chart review, Female Sexual Function Index (FSFI), Female Genital Self-Image Scale (FGSIS)	One (6.7%) had by narrowing at the introitus, which required intervention. The mean Female Sexual Function Index score was 28.6 (range, 24–31). Every individual achieved normal sexual function (FSFI ≥ of 25)
Mate-Kole et al 1990, A controlled study^112^	Outcome measures	4	40 AMAB (20 postop, 20 preop)	21–53	Sexual interest, sexual relationships	Chart review	Sexual interest during follow-up of 2 years for n = 20 following GAS was unchanged for 4, 15 were more active, none were less active. Sexual interest for n = 20 awaiting surgery remained unchanged for 17, 0 were more active, 3 were less active.
Morrison et al 2015, Long-Term Outcomes^113^	Outcome measures	4	83 AMAB	36–78	Dyspareunia, need for lubricant, mucorrhea, orgasmic capacity, sexual function	Phone interview; chart review	Of n = 44: 43 (98%) were able to orgasm. Of n = 34: 13 (38%) experienced dyspareunia. Of n = 27: 7 (26%) needed lubrication during intercourse. Average rating for satisfaction for n = 24 was 4.24 (1–5 scale).
Mukai et al 2017, Vaginoplasty with^114^	Outcome measures	4	15 AMAB	M 34.2 (SD 4.0)	Intercourse, pain, vaginal dimensions	Chart review	14 (93.3 %) had intercourse. 1 (6.7%) experienced discomfort during intercourse, because of neovaginal depth of 5–6 cm.
Papadopulos et al 2020, Psychological Pathologies^115^	Follow-up study	4	47 AMAB	18–57	Improvement of sex life, sexual orientation, change in sexual preference	Custom questionnaire	29 (61.7%) experienced an improvement of sex life following GAS. Prior to surgery: 12 (25.5%) were heterosexual, 22 (46.8%) homosexual, 11 (23.4%) bisexual, and 2 (4.3%) other. Following surgery n = 46, 15 (32.6) were heterosexual, 10 (21.7%) were homosexual gay, 21 (45.7%) were bisexual.
Papadopulos et al 2017, Combined vaginoplasty^116^	Follow-up study	4	40 AMAB	M 38.6 (SD 12.6)	Vulvar sensitivity, vaginal dimensions	Chart review and follow-up	Most women reported normal labial and vaginal sensitivity, and strong clitoral sensitivity.
Perovic et al 2000, Vaginoplasty in^117^	Outcome measures	4	89 AMAB	18–56	Orgasm, vaginal sensitivity, vaginal moisture, intercourse, vaginal dimensions	Interview	73 (82%) had orgasmed, 69 (79%) were having intercourse. Presence of vaginal moisture was satisfactory for 71 (80%) and unsatisfactory for 16 (18%)
Raigosa et al 2015, Male-to-Female^118^	Outcome measures	4	60 AMAB	19–50	Frequency and quality of intercourse, orgasm, vaginal dimensions, clitoral sensation	Interview (direct questioning during follow-up)	52 (86%) had regular intercourse. Clitoral sensitivity was acceptable and led to orgasm for all participants.
Reed et al 2015, Non-grafted Vaginal^119^	Outcome measures	4	18 AMAB	No data	Vaginal dimensions, sexual function	FSFI and clinical examination	Of n = 10: FSFI domain scores (lubrication 3,7; desire 3,5; arousal 4,0; orgasm 3,9; satisfaction 3,6; pain 4,7) were ≥ mid-range. Average total score was 23.4 (r 2–36).
Rehman et al 1999, The reported sex^120^	Outcome measures	4	28 AMAB	18–44	QoL, sexual orientation, sexual activity, type of sexual contact (oral, anal, vaginal, other), sexual satisfaction, orgasm (ability and importance), lubricant use, reasoning for sexual inactivity	Interview, self-developed questionnaire	15 (53.6%) had intercourse, all had some degree of pain during sex and all were using some form of lubricant. 7 (25%) had masturbated. 14 (50%) reported satisfaction from sexual activities and experienced orgasm most of the time, quality and intensity of orgasms were better postoperatively. 15 (53.6%) could orgasm, 7 (25%) orgasmed infrequently and 6 (21.4%) could not orgasm.
Rehman et al 1999 Formation of^121^	Outcome measures	4	10 AMAB	23–60	Clitoral sensitivity, QoL, sexual orientation, sexual activity, type of sexual contact (oral, anal, vaginal, other), sexual satisfaction, orgasm (ability and importance), lubricant use, reasoning for sexual inactivity	Interview, self-developed questionnaire	Every individual had intercourse and reported satisfactory sexual activities. 9 (90%) could orgasm (2 experienced neo-clitoral necrosis, 1 one could not achieve orgasm). Clitoris sensitivity was good with sensitivity to touch, vibration and light pressure in 8 (80%).
Salgado et al 2018, Primary Sigmoid^122^	Outcome measures	4	12 AMAB	M 47 (SD 15.4)	Vaginal dimensions, reported sensation, intercourse, satisfaction with depth, odour and excessive secretions	Chart review	5 (42%) had intercourse and reported satisfaction with vaginal depth and pleasurable sensitivity. None experienced malodour or excessive secretions.
Schroder et al 1999, New women^123^	Outcome measures	4	17 AMAB	35–58	Orgasm, masturbation, sexual fantasies, intercourse, relationship status, sexual orientation, sexual satisfaction, genital and breast sensitivity, arousal, sexual desire	Postoperative Male-to-Female Questionnaire (Carroll & Schroder, 1993a), New Woman's Gynaecological Index (NWGI) (Schroder, 1993), Stress Inventory (Carroll, 1985), Postoperative Male-to-Female Interview (Carroll & Schroder, 1993b), vaginal plethysmography	Mean self-reported rating of sexual satisfaction was 5.4 (0–10 scale, 10 is better). 11 (64.7%) could orgasm through masturbation (8 with ease, 3 with difficulty): 5 achieved multiple orgasms, and 5 ejaculated. Of n = 16 sexually active (approximately half had intercourse): 9 orgasmed during partnered activity (4 through penile-vaginal penetration, 3 through masturbation with a partner present).
Seyed-Forootan et al 2018, Autologous Fibroblast^124^	Group comparison/follow-up study	4	24 AMAB	Fibroblast: 28 SD 4y Amnion: 32 SD 3y	Vaginal dimensions, secretions, intravaginal sensitivity, orgasm, intercourse, satisfaction with intercourse	Self-developed questionnaire, interview, clinical examination of vaginal dimensions	Neo-vaginal sensitivity and lubrication was good for everyone. 18 (75%) had sexual experiences: 93.7% of the fibroblast and 87.5% of the amnion group were satisfied with orgasm and intercourse.
Sigurjónsson et al 2017, Long-Term Sensitivity^125^	Outcome measures	4	22 AMAB	23–63	Clitoral sensitivity, orgasm, sexual dysfunction	Semmes-Weinstein monofilaments, Bio-Thesiometer, self-developed scale	Average clitoral tactile thresholds were 12.5 g/mm^2^, average vibration threshold was 0.3 m. Surgical complications were not associated with diminished clitoral sensitivity or orgasmic capacity.
Soli et al 2008, Male to female^126^	Outcome measures	4	15 AMAB	21–60	Orgasm, clitoral sensitivity	Interview, self-developed questionnaire	7 (46.7%) experienced some form of climax during intercourse. Clitoral sensitivity was present and pleasant for every individual, and was present during digital examination by the authors.
Stanojevic et al 2007, Sacrospinous ligament ^127^	Outcome measures	4	62 AMAB	18–58	Ability to perform intercourse	Chart review	42 (76%) had intercourse.
Stein et al 1990, Follow-up observations^128^	Follow-up study	4	22 AMAB	20–49	Orgasm, vaginal intercourse, pain during intercourse, need for lubricants	Interview, physical examination	2 (14.3%) had never orgasmed, 6 (43%) seldom, 2 (14.3%) usually, NA for 4 (28.6%). Orgasm was not at all important for sexual satisfaction for 3 (21.4%), somewhat important for 6 (42.9%), very important for 1 (7.1%), NA for 4. 7 (31.8%) had intercourse: 1 (14.3%) used lubricants always, 3 (42.9%) often, 1 (14.3%) never, unknown for 2 (28.6%). Of n = 9: 6 (66.7%) had intimate lovers prior to GAS, 3 (33.3%) did not.
Tavakkoli Tabassi et al 2014, Fold-back^129^	Outcome measures	4	112 AMAB	M 25.8 (SD 3.3)	Satisfaction with function	Chart review	96 (85.7%) were satisfied with the appearance and function, 16 (14.3%) were dissatisfied (10 due to depth or stenosis, 6 due to aesthetics).
Thalaivirithan et al 2018, Application of embryonic^130^	Outcome measures	4	30 AMAB	21–42	Satisfaction with sexual function, frequency of sexual activities (oral, anal), orgasm, sexual satisfaction	Chart review	26 (86.6%) could orgasm, 30 (100%) had intercourse. Frequency of receptive, oral sex increased and anal sex decreased significantly following GAS. Satisfaction with sexual function and the appearance of the labia, vulva and clitoris was good-very good for 98% (5-point scale, unsatisfactory-very good). Sexual satisfaction was statistically (positively) correlated with vaginal function and depth, clitoral sensation, appearance of the vulva/labia minora, and natural lubrication and negatively correlated with depression scores.
Toolenaar et al 1993, The occurrence of^131^	Cross-sectional study	4	11 AMAB 6 women with MRKHS	AMAB: 22–48 MRKHS: 19–28	Intercourse, lubricant	Self-developed questionnaire, clinical examination	14 (82.4%) had regular intercourse, 3 (17,6%) did not have a sexual partner. 15 (88,2%) made use of lubricants. 13 (76.5%) experienced white discharge, 15 (88,2%) slight blood loss (6 following intercourse, 9 spontaneously). 3 experienced vaginal cramping (1 solely following sex).
van der Sluis et al 2016, Long-Term^132^	Outcome measures	4	24 AMAB	22–73	Intercourse, adequacy of vaginal dimensions, sexual arousal, orgasm, desire, lubrication, sexual satisfaction, discomfort	Female Sexual Function Index (FSFI), Amsterdam Hyperactive Pelvic Floor Scale for Women, (AHBBS) Female Genital Self-Imaging Scale (FGSIS), self-developed questionnaire	8 (89%) had intercourse, 1 had never. Mean FSFI satisfaction domain score was 4.2 (SD 1.3), mean score for orgasm 4.0 (SD 2.2). 8 (89%) had performed (frequent) penetrative intercourse, orgasm was possible through direct neo-clitoral stimulation. 8 (89%) had intercourse frequently, depth was adequate. 1 did not have intercourse (identified as asexual). Sexual arousal was possible, orgasm could be reached through neo-clitoral stimulation. Mean rating for neovaginal functionality was 7.3 (SD 1.8; 1–10 scale), appearance was 7.4 (SD 1.9)
van der Sluis et al 2016, Morphological spectrum^133^	Outcome measures	4	26 AMAB	19–52	Sexual activity (type of activity), sexual preference, lubricant use, condom use, vaginal symptoms (discharge, odour, pain)	Self-developed questionnaire, clinical examination (biopsies, vaginal swabs)	8 (31%) reported discharge, 4 (15%), 1 (4%) reported tenesmus, 4 (15%) neovaginal pain (3 of which during deep penetration).
Wagner et al 2010, Male-to-female^134^	Outcome measures	4	50 AMAB	25–52	Satisfaction with vaginal dimensions, orgasm, intercourse, pain during sex	Self-developed questionnaire	35 (70%) had achieved clitoral orgasm, 42 (84%) had regular intercourse (2 of which reported pain during intercourse).
Watanyusakul 2019, Vaginoplasty Modifications^13^	Outcome measures	4	580 AMAB	18–65	Vaginal depth	Not specified	Average depth >1 y follow-up was 16.0 cm.
Weyers et al 2009, Long-term assessment ^135^	Follow-up study	3	50 AMAB	M43.06 (SD 10.42)	Importance of sex, sexual functioning, relationships (status and quality), sexual preference	Female Sexual Function Index (FSFI), serum hormone levels, self-developed questionnaire	3 (6%) were not interested in sex. Median score for importance of sex in a relationship was 6 (interquartile range 5–9; 0–10 scale). Mean FSFI total score was 16.95 (SD 10.04). Overall FSFI scores were positively correlated with sexual satisfaction, general health perception and satisfaction with female appearance as perceived by others. FSFI total scores were highest for heterosexual individuals, intermediate for bisexual and lowest for homosexual individuals. There was no correlation between estradiol levels and mode of estrogen administration with testosterone levels and FSFI scores.
Wu et al 2009, Laparoscopic vaginal ^136^	Outcome measures	4	11 AMAB 67 DSD women 7 Cis women	AMAB: M 23.5 (SD 3.8) DSD: M 24.7 (SD 4,6) Cis: M 47.8 (SD 4.1)	Intercourse (time between surgery and first contact), orgasm, lubrication, satisfaction with sexual life, vaginal dimensions, dyspareunia, bleeding during intercourse	Chart review, self-developed questionnaire	71 (88.8%) was sexually active. More than half reported frequent orgasms, and 90% reported adequate lubrication for intercourse. None reported dyspareunia, use of external lubrication, or mild bleeding during intercourse.
Zavlin et al 2017, Male-to-Female^80^	Follow-up study	4	49 AMAB, 40 with questionnaire results	M 38.6 (|SD 12.6)	Intercourse (satisfaction with), (satisfaction with) orgasmic capacity, orgasm, masturbation, (satisfaction with) clitoral sensitivity, pain during masturbation or intercourse, sexual orientation	Self-developed questionnaire	Mean scores for satisfaction on 0–10 scale (10 is better) with: orgasm 8.21 (SD 2.47, n = 38); preoperative intercourse 3.29 (SD 2.75, n = 7); postoperative intercourse 6.7 (SD 2.03, n = 23). Mean rating of clitoral sensation was 8.53 (SD 1.93, n = 40), and erogenous clitoral sensation was 8.48 (SD 2.04, n = 40). Mean rating of pain during masturbation or intercourse was 2.33 (SD 2.89; n = 39; 10 is extreme pain). 7 (17.5%) engaged in regular intercourse prior to GAS, 57.5% following GAS.
Zavlin et al 2019, Age-Related Differences^137^	Cross-sectional study	4	40 AMAB	19-66	Sexual orientation, marital status, frequency of intercourse, sexual preference, satisfaction with intercourse, sexually active,	Self-developed questionnaire	Following GAS, younger individuals were mostly attracted to men (52.6%), later-onset individuals mostly to women or both (85.7%). Younger trans individuals were more frequently sexually active (73.7% vs 42.9%).

Twenty-four studies discussed whether participants could attain orgasm.^79^^,^^87^^,^^88^^,^^90^^,^^91^^,^^95^^,^^96^^,^^98^, ^99^, ^100^, ^101^^,^^104^^,^^106^, ^107^, ^108^, ^109^^,^^113^^,^^120^^,^^121^^,^^123^^,^^125^^,^^126^^,^^128^^,^^134^ A majority (about 70%) could achieve orgasm,^79^^,^^87^^,^^88^^,^^90^^,^^91^^,^^95^^,^^98^, ^99^, ^100^, ^101^^,^^104^^,^^106^, ^107^, ^108^, ^109^^,^^113^^,^^117^^,^^120^^,^^121^^,^^123^^,^^125^^,^^126^^,^^128^^,^^134^^,^^143^ whereas less than 10% could not or had not,^79^^,^^88^^,^^90^^,^^95^^,^^99^^,^^100^^,^^107^^,^^109^^,^^120^^,^^125^^,^^143^ 10% had not tried^79^^,^^90^^,^^99^^,^^125^, and another 10% chose ‘not applicable.’^107^ Five studies applied the Female Sexual Function Index (FSFI) and reported a mean orgasm domain score ranging between 2.82–3.9 (scores CIS women without sexual problems 5.1 SD1.1).^79^^,^^90^^,^^92^^,^^119^ Finally, Zavlin et al^80^ found a mean frequency of achieving orgasm of 6.73 (SD 3.32) during masturbation and 6.52 (SD 3.11) during intercourse.^80^

Over half of participants masturbated regularly,^60^^,^^62^^,^^100^^,^^107^^,^^108^^,^^120^^,^^121^^,^^123^^,^^140^^,^^144^, ^145^, ^146^ and every participants had engaged in receptive vaginal activity.^79^^,^^89^^,^^90^^,^^96^^,^^104^^,^^106^^,^^114^ Some, however, failed at penetrative sex, either because of short time since surgery, inadequate vaginal dimensions or pain.^79^^,^^90^^,^^114^^,^^128^ Reporting on other sexual activities, for example, receptive anal sex^60^^,^^100^^,^^109^^,^^121^ and- active and passive- oral sex was limited.^100^^,^^120^ Whether GAS brings about a change in sexual activities remains unclear, this data and associations between presurgical and postsurgical sexual a^142^ctivity were not provided.^92^^,^^96^^,^^107^^,^^109^^,^^128^^,^^147^

Overall sexual satisfaction or satisfaction with sex life (77%),^79^^,^^84^^,^^90^^,^^92^^,^^94^^,^^107^^,^^108^^,^^113^^,^^119^^,^^135^^,^^142^^,^^143^ satisfaction during sex,^80^^,^^120^^,^^123^ and satisfaction with orgasmic function was present in a majority of postsurgical individuals.^87^^,^^91^

Some studies reported on sexual dysfunction, where sexual wellbeing was mostly defined as a lack of sexual dysfunction. Ten studies discussed pain during receptive vaginal sex in AMAB individuals; one third experienced either pain in or around the introitus, deep or superficial dyspareunia or vulvodynia.^79^^,^^80^^,^^84^^,^^88^^,^^100^^,^^113^^,^^120^^,^^125^^,^^134^^,^^143^ Difficulties for AMAB individuals during penetrative sex were described in 10 studies: a third experienced difficulties or were unable to perform receptive vaginal sex, due to inadequate vaginal depth or width.^79^^,^^85^^,^^89^^,^^90^^,^^98^^,^^100^^,^^114^^,^^120^^,^^123^

### Breast Augmentation

*Statement #10 The gender surgeon is advised to suspend breast augmentation in trans individuals AMAB until 12 months of hormone therapy have been completed (Level III Grade C)*

*Statement #11 The gender surgeon is advised to consider fat grafting as available alternative to or extension of implant use in trans individuals AMAB (Level IV Grade C)*

*Statement #12 The gender surgeon should counsel the trans individuals AMAB on risks associated with implant use (Level III Grade B)*

#### Evidence

Feminizing hormone therapy might yield unsatisfactory breast development. Individuals might not reach the final stages of breast development and opt for breast augmentation,^148^ which may improve feminine contour, increase subjective feelings of femininity, aid in passability and adjustment to the female gender role, and consequently increase sexual and psychosocial wellbeing.^149^ Choice of augmentation technique is dependent on both patient and surgeon's preference.

Patients should be counselled on implant type, implant surface and placement. Care must be taken to properly centre the implant under the nipple-areolar complex (NAC) to prevent diverging nipples and wide cleavage.^150^^,^^151^ Implant placement more medial of the NAC can be considered in individuals with a laterally placed NAC, or surgical medialization of the NAC can be pursued.^152^^,^^153^

Implant use is advantageous in regard to predictability of results. Associated risks, however, are capsular contracture, implant malposition, autoimmune responses and Breast Implant Associated Anaplastic Large Cell Lymphoma (BIA-ALCL).^154^ Fat grafting provides an alternative to implant use, eliminating the risk of BIA-ALCL.^155^ Fat grafting can be done solitary, in conjunction with implant use, as well as during secondary corrections.^156^ Breast cancer screening should be performed according to the local guidelines.

#### Sexual Wellbeing

One study reported on sexual wellbeing following breast augmentation, finding a significant increase in sexual wellbeing four months postoperatively (Table 3*)*.^149^

**Table 3:** Sexual wellbeing following breast augmentation

Study	Design	LoE	Sample size	Age range	Sexual Topics	Methods/Tools	Outcomes regarding Sexual Wellbeing
Weigert et al 2013 Patient satisfaction^149^	Follow-up study	4	35 AMAB	18,9–62,6	Sexual wellbeing	Breast-Q	Sexual wellbeing: 4 mo post-op: ↑ 34 points 12> mo post-op: ↑ 33 points

### Vocal Feminization Surgery

*Statement #13 The Ear, Nose and Throat (ENT) surgeon should consider vocal feminization surgery when treatments with speech-language pathologists have yielded unsatisfactory results in trans individuals AMAB (Level IV Grade D)*

#### Evidence

Consulting an ENT surgeon prior to starting with speech therapy is recommended, to rule out vocal cord anatomy and functioning anomalies. Surgery may be considered if speech therapy yields unsatisfactory results. Surgical results are unpredictable.^157^

#### Sexual Wellbeing

We found no studies on sexual wellbeing after vocal feminization surgery in AMAB individuals.

### Facial Feminization Surgery (FFS)

*Statement #14 The gender surgeon should treat secondary facial aspects before beginning structural facial GAS in trans individuals AMAB (Level IV Grade C)*

*Statement #15 The gender surgeon is advised to consider adjustments of the frontonasal-orbital complex, the nose, the lower jaw and the thyroid cartilage when performing FFS in trans individuals AMAB (Level IV Grade C)*

#### Evidence

Secondary or non-skeletal facial aspects, such as hair and hairline, facial hair, skin texture, and the distribution and volume of facial fat can be heavily determined by hormonal influence, generally responding well to hormone therapy, which in itself does not interfere in any way with surgery. In addition, many AMAB will opt to undergo laser facial hair removal or electrolysis. It is therefore preferable to treat the secondary aspects before beginning structural or skeletal facial gender confirmation surgery (at least 12 months before surgery). Obtaining a female bone structure while maintaining male secondary aspects is self-defeating to both the result and the perception of the patient's femininity. The expectations for the results will be more real if the initial anticipation, both psychological and physical, is realistic.^158^

Adjustments of the frontonasal-orbital complex, the nose, the lower jaw and the thyroid cartilage are elements of Facial Feminization Surgery (FFS).^159^, ^160^, ^161^, ^162^, ^163^. Forehead reconstruction and hairline corrections-approached through a coronal or hairline incision^164^ open up the upper face.^165^, ^166^, ^167^, ^168^ Lower jaw contouring corrects the lower face by reducing the transverse and vertical dimensions of the jaw, softening the angle of the mandible, improving jawline contour and adjusting of the volume, format and position of the chin.^169^, ^170^, ^171^, ^172^, ^173^, ^174^, ^175^, ^176^, ^177^, ^178^ The midface may be addressed with rhinoplasty.

#### Sexual Wellbeing

We found no studies on sexual wellbeing after facial feminization surgery (FFS).

## Surgical Procedures for Masculinizing GAS

This section addresses different types of masculinizing GAS, with their respective results regarding sexual wellbeing. Decision making is on patient preference and patient specifics.

### Mastectomy

*Statement #16 The gender surgeon is advised to consider performing a mastectomy in trans individuals AFAB prior to starting living in the desired gender, in selected cases of severe dysphoria and with large volume chests (Level IV Grade D)*

#### Evidence

**Figure 1:**
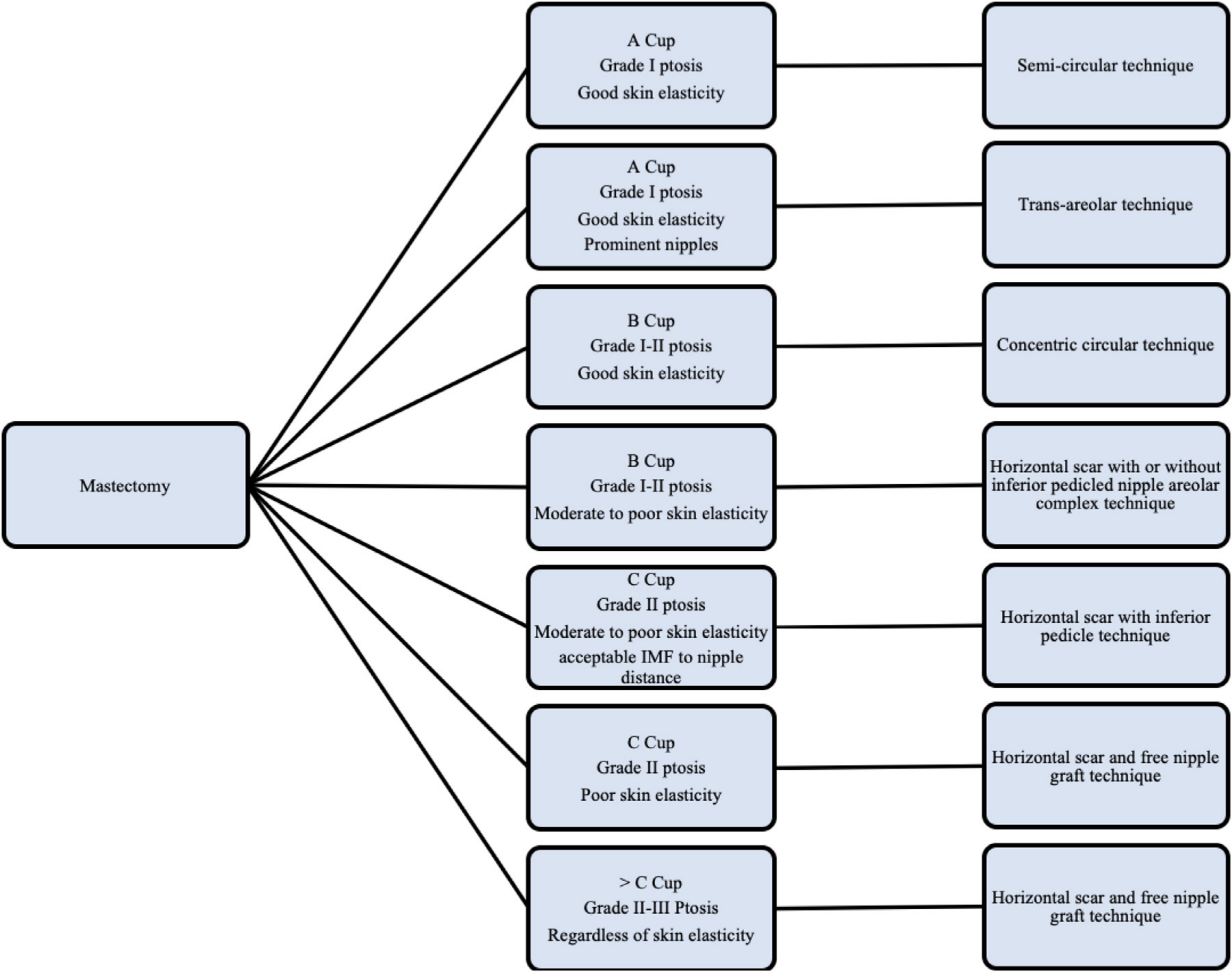
Breast size, ptosis and skin elasticity in regards to mastectomy techniques. Grade I ptosis correspond with the areola placed on the level of the inframammary crease (IMC), Grade II corresponds with the nipple below the IMC and above the level of the gland, grade III corresponds with the areola below the IMC and below the contour of the gland.^224^ Cup size A corresponds with a breast volume of less than 150 cc in individuals with an under bust circumference of 70–75 cm; cup size B corresponds with a volume of 250–299 cc with an under bust of 70–75 cm, cup size C corresponds with 300–349 cc with an under bust of 70–75 cm.^225^^,^^226^

Masculinizing chest surgery may improve dysphoria, body image, psychological and sexual wellbeing and overall quality of life, and carries importance for AFAB individuals in this regard.^38^^,^^179^^,^^180^ Excess skin and glandular tissue is excised, whilst preserving subcutaneous fatty tissue to ensure flap vascularity and facilitating an acceptable contour of the chest wall. Techniques include *the semi-circular technique, trans-areolar technique, concentric circular technique, free nipple technique with horizontal scar* and *the inferior pedicled mammaplasty technique*, for “Breast size and mastectomy techniques” see Figure 1.^156^^,^^181^

The semi-circular technique is applicable for very small breasts. The scar is confined to the lower half of the areola. Limited surgical exposure carries risk of increasing postoperative hematomas.^181^

The trans-areolar technique allows for correction of the nipple by nature of the scar placement through the areola, horizontally. This carries increased risk of postoperative hematomas.^156^

The concentric circular technique allows for correction of excess skin through an ellipse, or circle shaped, incision. May lead to skin puckering around the areola, areolar widening- due to traction- and nipple necrosis.^156^^,^^181^

The horizontal scar and free graft technique allow for correction of very large breasts. It is met with large scars, NAC depigmentation and partial graft loss.^148^^,^^181^ The inferior pedicled mammaplasty technique is comparable, whilst transposing the NAC on an inferior pedicle, instead of a free graft.^156^

Masculinizing hormone therapy may ameliorate breast cancer risk.^155^ Patients should, however, be made aware of residual breast tissue on the entire plane of dissection.^182^

#### Sexual Wellbeing After Mastectomy

Three studies reported on sexual wellbeing after mastectomy, accessible in Table 4*.* These publications mostly focused on sexual relationships and quality of sex life. Mastectomy improved quality of life and confidence in social and sexual situations, in both dressed and undressed situations. Reduction of dysphoria, improvement of body image and confidence following mastectomy affected sexual relationships positively.^38^^,^^183^^,^^184^

**Table 4:** Sexual wellbeing following mastectomy

Study	Design	LoE	Sample size	Age range	Sexual Topics	Methods/Tools	Outcomes regarding Sexual Wellbeing
Esmonde et al 2019 What is “Nonbinary”^183^	Outcome measure	4	58 AFAB	18–48	Sexual orientation, relationship status	Retrospective chart review	Surgery improved quality of life, sex life, and comfort in physical appearance with and without clothes.
Poudrier et al 2019 Assessing Quality of Life^184^	Cross sectional study	4	58 AFAB	18–58	Satisfaction with sex life, sexual confidence (sexually confident without a shirt, likely to remove shirt for sex), comfortable during sexual activities, sexually attractive in clothes, when unclothed	Custom questionnaire	Most respondents rated their quality of life and sexual confidence before top surgery as very low. Post-op, quality of life and sexual confidence improved significantly in all domains.
van de Grift et al 2016 Subcutaneous Mastectomy^38^	Follow-up study	4	26 AFAB	18–59	Sexual orientation, sufficiency as sexual partner, pleasure of sexual activities	Appearance Schemas Inventory Revised, Body Image Quality of Life Inventory (BIQLI), Body Image Scale for Transsexuals (BIS), Multidimensional Body-Self Relations Questionnaire, Rosenberg Self-Esteem Scale, Perceived Effect of Surgery (self-developed)	Mastectomy positively influences body image. Positive evaluation of the body and decreased dysphoria during social situations –> increased quality of life and self-esteem.

### Removal of the Female Sexual Organs

*Statement #17 The gender surgeon is advised to counsel on intervention options salpingo-oophorectomy, hysterectomy and vaginectomy, dependent on trans individuals AFAB preference (Level IV Grade D)*

*Statement #18 The gender surgeon should advise routine screening, by a general practitioner or gynaecologist, for cervical cancer if the uterus remains in situ in trans individuals AFAB (Level IV Grade D)*

#### Evidence

Indications for hysterectomy include persistent blood loss, dysphoric feelings, unwanted discharge or lubrication, and within the context of GAS with urethral lengthening. Vaginectomy, and subsequent hysterectomy, maybe a required when opting for urethral lengthening, because of higher risks of developing complications.^185^ The choice for salpingo-oophorectomy is dependent on patient preference-only.

Routine screening for cervical cancer should be continued. Screening for endometrial cancer should be commenced in older patients with vaginal blood loss. Evidence for positive effects of routine screening for ovarian cancer in trans individuals AFAB are lacking. We would therefore not encourage routine screening, currently. Consider counselling on HPV self-sampling to increase testing rates.

#### Sexual Wellbeing After Removal of the Female Sexual Organs

We found no results of studies on sexual wellbeing in trans individuals AFAB after removal of the female sexual organs.

### Metaidoioplasty

*Statement #19 The gender surgeon should suggest metaidoioplasty as a variant of one-stage neo-phalloplasty in masculinizing genital GAS in trans individuals AFAB (Level IV, Grade C)*

*Statement #20 The gender surgeon should advise postoperative genital stretching, to prevent genital shrinkage after metaidoioplasty in trans individuals AFAB (Level IV, Grade D)*

*Statement #21 The gender surgeon should engage in shared decision making and counselling in choosing type of genital masculinisation surgery in trans individuals AFAB (Level II Grade D)*

*Statement #22 The gender surgeon is advised to counsel trans individuals AFAB on the specific advantages of a metaidoioplasty being: preservation of sexual arousal, erogenous sensation and spontaneous erections (Level IV Grade D)*

*Statement #23 The gender surgeon is advised to counsel trans individuals AFAB on the specific disadvantagesof a metaidoioplasty being: possible lack of length for penetration or to void standing (Level IV Grade D)*

#### Evidence

Metaidoioplasty can be carried out as a one-stage procedure, with removal of the female sexual organs, optional urethral lengthening, scrotoplasty and testicular implants placement.^186^^,^^187^ The majority of the lengthening of the clitoris is achieved through ventral division of the urethral plate. Additional length may be obtained by dividing the clitoral suspensory ligaments.^188^^,^^189^ Clitoral length should be sufficient for voiding while standing when urethral lengthening is requested, studies do not report on what is meant by “sufficient”. The technique for urethral lengthening is dependent on patient anatomy and tissue quality, options include *pedicled labia minora grafts and buccal mucosa grafts*. Having non-overlapping suture lines and covering suture lines with vascularized tissue prevent fistulation.^146^^,^^190^^,^^191^ Scrotoplasty is achieved through labial tissue,^192^ testicular implants can be inserted.^192^^,^^193^ Postoperative genital stretching- either manual, vacuum-assisted or withPDE5 inhibitors- may prevent genital shrinking. However, evidences on this topic are poor.^189^^,^^191^^,^^193^

Choices on genital masculinization surgery are increasingly being made through shared decision-making.^51^ The surgeon should inform the patient on the different options (metaidoioplasty and phalloplasty, the techniques available, their advantages and disadvantages, limitations with producing ‘ideal’ results and possible risks and complications.^52^, ^53^, ^54^

Common minor complications are urinary tract infections and bladder overactivity. Minor urethral fistulae and strictures can be managed non-surgically, revision surgery is indicated for major fistulae and strictures,^186^^,^^194^ regenerating vaginal mucosa,^186^^,^^193^^,^^194^ and displaced or expelled testicular implants.

#### Sexual Wellbeing After Metaidoioplasty

Sexual wellbeing was reported on in six publications, provided in Table 5.^146^^,^^187^^,^^189^^,^^193^^,^^195^, ^196^, ^197^ Five of these publications may contain an overlap in study population.^146^^,^^187^^,^^189^^,^^193^^,^^197^

**Table 5:** Sexual wellbeing following metoidioplasty

Study	Design	LoE	Sample size	Age range	Sexual Topics	Methods/Tools	Outcomes regarding Sexual Wellbeing
Djordjevic et al 2013 Comparison of Two Different Methods^146^	Group comparison / Follow-up study	4	207 AFAB	18–62	Neo-phallic dimensions, erection of the clitoris, sensation, sexual arousal, masturbation, orgasm, sexual intercourse	Chart review	Length of neophallus ranged from 4 cm–10 cm. Majority: pleased with the aesthetic appearance. All: erection of the clitoris and completely preserved sensation None: problems or difficulties in sexual arousal, masturbation, or orgasms. Patients who reported sexual intercourse with partners: length of the neophallus was inadequate for full penetration
Djordjevic et al 2009 Metoidioplasty as a Single^189^	Outcome measure	4	82 AFAB	18–54	Erection of the clitoris, sensation	Chart review	All: erection of the clitoris and completely preserved sensation
Djordjevic et al 2018 Novel surgical techniques in^193^	Outcome measure	4	694 AFAB	18–62	Erection, sexual arousal, masturbation, orgasm, neo-phallic sensation (tactile, erogenous), sexual intercourse	Retrospective chart review	Majority: pleased with the aesthetic appearance. All: erection of the clitoris and completely preserved sensation None: problems or difficulties in sexual arousal, masturbation, or orgasms. Patients who reported sexual intercourse with partners: length of the neophallus was inadequate for full penetration
Stojanovic et al 2017 One-Stage Gender-Confirmation Surgery^187^	Outcome measure	4	374 AFAB	18–43	Sexual function, quality of erection, sexual arousal, erogenous sensation	Chart review	Majority: complete satisfaction with appearance, overall complete sexual satisfaction and always experienced orgasm during masturbation. All: good quality of erection, sexual arousal, and completely preserved erogenous sensation.
Takamatsu et al 2009 Labial ring flap^195^	Outcome measure	4	43 AFAB	18–33	Sensitivity, intercourse	Chart review	One patient: intercourse with his female partner. No complaints of a reduction of erogenous sensation on the clitoral glans.
Van de Grift et al 2019 Transmen's Experienced Sexuality^196^	Cross sectional study	4	38 AFAB	Average 40 (SD 10)	Arousability, sexual sensation, sexual pleasure, interest in sex, sexual initiative, orgasmic capacity/intensity, satisfaction with genital appearance, satisfaction with sexual functioning/relationships, sexual orientation, use of genitals during sexual contact, the influence of GAS on sexual outcomes	Custom questionnaires	Not specific for metaidoioplasty Majority: sexually active. Areas of improvement after surgery: use and enjoyment of both chest and genitals, arousability, sexual interest, and pleasure. Genital GAS positively impacts transmen's sexuality, issues with genital sensation or penetration may exist
Vukadinovic et al 2014 The Role of Clitoral Anatomy^197^	Outcome measure	4	97 AFAB	18–41	Sexual arousal, masturbation, orgasm, ability to perform penetration, sexual activity (type of activity), quality of erection, erogenous sensation, sexual satisfaction	Biographical Questionnaire for Transsexuals and Transvestites, self-developed questionnaire	Majority: complete satisfaction with appearance, overall complete sexual satisfaction and always experienced orgasm during masturbation. All: good quality of erection, sexual arousal, and completely preserved erogenous sensation. Patients who reported sexual intercourse with partners: length of the neophallus was inadequate for full penetration

Metaidoioplasty provided satisfying aesthetics and positive outcomes regarding sexual wellbeing, sensation, erectile function and orgasm. Sexual arousal, which resulted in erection, and sensation were present in every individual.^146^^,^^187^^,^^189^^,^^193^^,^^197^ A majority of participants had masturbated, which had not resulted in orgasm for everyone,^146^^,^^187^^,^^193^^,^^197^ and insufficient length for penetrative sex proved the main disadvantage.^146^^,^^193^^,^^197^ An average of 10% initially opting for metaidoioplasty, pursued conversion to phalloplasty at a later stage.

### Phalloplasty or Total Phallic Construction (TPC)

*Statement #24 The gender surgeon should provide trans individuals AFAB the radial free forearm flap (RFFF) phalloplasty as the technique of choice for masculinizing genital GAS in patients desiring a full-size phalloplasty, accompanied by urethral lengthening (Level IV, Grade C)*

*Statement #25 The gender surgeon should be capable to offer alternatives to the free forearm flap (RFFF) in trans individuals AFAB, like: the antero-lateral thigh flap (ALT), pedicled pubic phalloplasty (PP) or latissimus dorsi (LD) phalloplasty as an alternative full-size phalloplasty with or without urethral lengthening (Level IV, Grade C)*

*Statement #26 The gender surgeon is advised to combine a skin flap (RFFF or pedicled superficial circumflex iliac artery perforator flap) for urethral lengthening, when a single-flap reconstruction cannot be accomplished, in trans individuals AFAB (Level IV, Grade C)*

*Statement #27 The gender surgeon should engage in shared decision making and counselling in choosing type of genital masculinisation surgery, in trans individuals AFAB (Level II Grade D)*

*Statement #28 The gender surgeon is advised to counsel, trans individuals AFAB, on the specific advantagesof a phalloplasty being: volume in genital area when dressed, possibility of penetration (Level IV Grade D)*

*Statement #29 The gender surgeon is advised to counsel, trans individuals AFAB, on the specific disadvantagesof a phalloplasty being: cutaneous and erogenous sensitivity can be poor (Level IV Grade D)*

#### Evidence

Total Phallic Construction (TPC) aims at creating a phallus with acceptable aesthetics, a degree of cutaneous and erogenous sensitivity and sufficient bulk to house potential erectile prostheses. Standing urination is achieved through urethral lengthening. Techniques include: *the radial free forearm flap, suprapubic pedicled pubic (PP), superficial circumflex iliac artery perforator flap (SCIP), antero-lateral thigh flap (ALT)*and *the latissimus dorsi flap (LD) techniques.* Table 6 shows donor sites, flap types, urethral lengthening options, sensation, advantages and disadvantages of all flaps. The RFFF presents superior aesthetics and functionality, allowing for integrated urethral lengthening,^198^, ^199^, ^200^, ^201^ compared to other techniques, and is considered “the gold standard” by some.^202^ RFFF-TPC is commonly carried out in three stages of six-month intervals: the creation of the phallus and neo-urethra, microsurgically anastomosed; glans- and coronaplasty using a full-thickness skin graft; and potential erectile prosthesis implantation. Proper preparation reduces donor-site morbidity and full-thickness skin grafts result in less scarring and discoloration.^203^^,^^204^ Interposition of collagen-matrix, between the recipient site and a split-thickness skin graft, simulates the appearance of a full-thickness skin graft without hair. The pedicled pubic phallus is fashioned from a cutaneous flap that is raised from the inferior aspect of the abdominal wall, allowing for primary closure of the donor site.^205^ Both the PP flap and the *SCIP* flap are Hair-Bearing, have poor cutaneous sensitivity and difficult urethral lengthening. Direct urethral lengthening is carries increased risk of complications, delayed incorporation of a free radial artery based flap bears less complications and is not prone to sacculation.^206^ The ALT flap is a reasonable option^207^^,^^208^ established from a pedicled Fascio-Cutaneous flap of the perforating vessels of the vastus lateralis and rectus femoris. Pedicle length is commonly sufficient for tunnelling to the pubic area, obviating microsurgical techniques. A thick fat layer and hair complicate integrated urethral lengthening, often requiring other flaps for urethral lengthening.^209^ Cutaneous sensation is moderate due to the presence of one cutaneous nerve within the flap. The donor site is usually covered with split-thickness skin grafts. The LD flap allows for a larger myocutaneous flap based on the thoracodorsal artery and nerve, allowing for primary closure of the donor site. Cutaneous sensation is poor as a single motor nerve is available.^210^ Urethral lengthening requires multi-stage buccal mucosa and labia minora flaps.

**Table 6:** Total phallic construction: donor sites, flap types, urethral lengthening options, sensation, advantages and disadvantages of all flaps

Type of TPR	Donor site	Type of flap	Neo-urethra	Sensation	Advantage	Disadvantage
Radial artery forearm free flap (RAFFF)	Non-dominant forearm	Free flap	Direct possible	Sensate	Good aesthetics Tip-reaching urethral lengthening	Significant donor-site scarring
Suprapubic pedicled flap	Lower abdomen	Pedicled flap	Staged radial artery-based flap	Poor cutaneous sensitivity	Operating time is relatively short Donor site scarring is limited to the abdomen	Poor cutaneous sensitivity Difficult urethral lengthening
Superficial circumflex iliac artery perforator (SCIP) flap	Groin/flank	Pedicled flap	Direct double SCIP or with another free flap	No data	Operating time is relatively short Donor site scarring is limited to the abdomen	No data on sensation
Antero-lateral thigh (ALT)	Upper leg	Pedicled flap	Direct or with another free flap	Moderate cutaneous sensitivity	Pedicle length sufficient for tunnelling	Patient selection important: subcutaneous fat layer Moderate cutaneous sensitivity
Latissimus dorsi (LD)	Side of chest under arm	Free flap	In 2 stages with buccal mucosa grafts	Poor cutaneous sensation	Minimal donor site morbidity	Second-stage urethral lengthening with buccal mucosa grafts

As stated in the metaidoioplasty section choices on genital masculinization surgery are increasingly being made through shared decision-making.^51^ The surgeon should inform the patient on the different options (metaidoioplasty and phalloplasty, the techniques available, their advantages and disadvantages, limitations with producing ‘ideal’ results and possible risks and complications.^52^, ^53^, ^54^

#### Sexual Wellbeing After Phalloplasty

Eighteen studies reported on sexual wellbeing after phalloplasty, provided in Table 7.^26^^,^^62^^,^^196^^,^^211^, ^212^, ^213^, ^214^, ^215^ Outcomes for various surgical techniques were often pooled, separate results were rarely provided. Four pairs of studies may have had an overlap in study population.

**Table 7:** Sexual wellbeing following phalloplasty

Study	Design	LoE	Sample size	Age range	Sexual Topics	Methods/Tools	Outcomes regarding Sexual Wellbeing
Bettocchi et al 2005 Pedicled pubic^205^	Outcome measure	4	85 AFAB	19–54	Possibility to have penetrative sexual intercourse	Chart review	Penetrative sex: 16 without penile prosthesis 17 with prosthesis (n = 8 malleable; n = 9 Dynaflex) 6 lost malleable prosthesis through skin erosion
Djordjevic et al 2018 Novel surgical^193^	Outcome measure	4	694 AFAB	18–62	Erection, sexual arousal, masturbation, orgasm, neo-phallic sensation (tactile, erogenous), sexual intercourse	Retrospective chart review	Metaidoioplasty: None reported difficulties or problems related to sexual arousal, masturbation or orgasm Phalloplasty: Erogenous sensation based on clitoral stimulation in all None reported problems or difficulties in sexual arousal, masturbation or orgasms Sexual intercourse with complete penetration was totally adequate in all with penile implants
Falcone et al 2018 Outcomes of^220^	Outcome measure	4	247 AFAB	21–69	Phallic sensation, sexual intercourse, orgasm, partner satisfaction	Chart review, Self-developed questionnaire	Satisfactory phallic sensation: 83%. Penetrative sexual intercourse: 77% Orgasm: 61% Partner satisfaction: 60%
Fang et al 1999 Phalloplasty in^219^	Outcome measure	3	22 AFAB	No data	Erotic sensation of the clitoris, neophallus sensation, orgasm, masturbation, intercourse	Chart review	All preserved clitorises had erotic sensation Erotic sensation on neophallus: 8 (shaft, hypothesis: coapted forearm cutaneous nerves to ilioinguinal and iliohypogastric nerves) Orgasm by masturbation the neophallus only: 1 (pudendal nerve anastomosis) Regular sex: 9 Orgasm during intercourse: 9 Sexual performance: satisfactory 9
Garaffa et al 2010 Total phallic^198^	Outcome measure	4	115 AFAB	20–55	Neo-phallus sensation, sexual activity	Self-developed questionnaire	Complete phallus sensation: 71.5% Sensation within neourethra: 14,7% Insensate phallus: 6% Recent surgery too early to assess 5,2% Phalluses that were lost: 2,6%
Garcia et al 2014 Overall satisfaction^221^	Outcome measure	3	5 AFAB	M 35.1 (SD 2.23)	Erogenous sensation, orgasm, masturbation	Interview	SP: Suprapubic Phalloplasty 10 RAP-: Radial Artery free flap Phalloplasty without nerve anastomosis 5 RAP+: Radial Artery free flap Phalloplasty with nerve anastomosis 10 Orgasm pre-op: SP: 9 of 10 RAP -: 4 of 5 RAP +: 7 of 10 Orgasm post-op SP: all RAP -: all RAP +: 8 of 10 Orgasm with direct stimulation of buried clitoris: SP: all RAP -: all RAP +: 4 of 6 masturbate with phallus SP: 9 of 10 RAP -: all RAP +: 9 of 10 diminished orgasm after penile prosthesis: none regret of surgery: none
Leriche et al 2008 Long-term outcome^216^	Outcome measure	4	56 AFAB	20–44	Cutaneous sensitivity, erogenous sensitivity, sexual satisfaction, satisfactory sexual intercourse with penetration	Self-developed questionnaire	Cutaneous sensitivity of the phalloplasty: 83% Erogenous sensitivity: 9% Sexual satisfaction (penetration with penile implant): 51%
Monstrey et al 2009 Penile reconstruction^202^	Outcome measure	4	280 AFAB, 7 men with various conditions	No data	Sensitivity, improvement in sexuality, orgasm, ability to perform penetration.	Chart review	Tactile sensitivity: 100% Improvement in sexuality: 80% Sexually active: 100% Explantation rate erection prosthesis: 44% Sexual intercourse with penetration: 80%
Noe et al 1974 The surgical construction (230)	Outcome measure	4	12 AFAB	No data	Ability to perform penetrative sex, orgasm	Chart review	Intercourse: 10 of 12 Orgasm: 9 of 12
Papadopulos et al 2001 Usefulness of free^214^	Outcome measure	4	24 AFAB	No data	Intercourse, phallic sensitivity, pain	Self-developed questionnaire, clinical and radiologic examination	Questionnaire score: 2 = excellent; 1 = acceptable; 0 = poor Sensibility: Free forearm flap: 1,41 Free fibula flap: 0,66 Intercourse: Free forearm flap: 0,83 Free fibula flap: 2 Pain: Free forearm flap: 1,41 Free fibula flap: 1,5
Ranno et al 2007 Neo-phalloplasty with^212^	Cross-sectional study	4	18 AFAB	24–38	Contractile power (measured and asked)	Clinical examination of phallic contraction power (measurement of weight lifted and electromyography)	"Paradox erection": 18 Neo-phallus length (relaxed): 7–17 cm (mean 12.2 cm) Circumference (relaxed): 13–20 cm (mean 13.7 cm) Contract the muscle: all Average length reduction: 3.08 cm Average circumference enlargement: 4 cm
Ranno et al 2008 An objective^211^	Outcome measure	4	22 MtF	No data	Intercourse, penile dimensions (relaxed and contracted)	Self-developed questionnaire, clinical examination	Sexual intercourse without the need for prosthesis Successful contraction when 2 cm shortened with average weight 1129 gr
Schaff et al 2009 A new protocol^215^	Outcome measure	4	37 AFAB	No data	Intercourse (and it's quality), sensation	Self-developed questionnaire, chart review	Fibula flap, sexual intercourse: Excellent: 100% Sensibility minor to forearm flap: 83,3%
Van de Grift et al 2019 Transmen's experienced^196^	Cross sectional study	4	38 AFAB	Average 40 (SD 10)	Arousability, sexual sensation, sexual pleasure, interest in sex, sexual initiative, orgasmic capacity/intensity, satisfaction with sexual functioning/relationships, sexual orientation, use of genitals during sexual contact, the influence of GAS on sexual outcomes	Custom questionnaires	Sexual partner: 78% Sexual attraction: mainly women Use of chest during sex: 40% Use of genitals during sex: 78% Satisfaction with sexual function: Metaidoioplasty: 63,5% Phalloplasty: 28% Arousability: ↑32%; = 65%; ↓3% Sexual sensation: ↑45%; = 39%; ↓16% Sexual pleasure: ↑40%; = 47%; ↓13% Interest in sex: ↑39%; = 45%; ↓16% Sexual initiative: ↑26%; = 58%; ↓16% Orgasmic intensity: ↑21%; = 52%; ↓23% Orgasmic capacity: ↑18%; = 52%; ↓26%
Vesely et al 2007 New technique^217^	Outcome measure	4	22 AFAB	24–38	Ability to have sexual intercourse	Self-developed questionnaire	Sexual intercourse: 42% Muscle movement: 100% to stiffen the penis and/or move the penis during sexual intercourse Penetration, but too short to keep inside: 11% No sexual activity: 16% Penetration not possible: 31% (too wide, too small, or too soft)
Wierckx et al 2011 Quality of life and^26^	Follow-up study	4	49 AFAB	22–54	Relationship status, sexual preference, sexual activities (frequency, type of activity, pain), sexual wellbeing, masturbation, sexual satisfaction, orgasm, arousal	Self-developed questionnaire	Sexual orientation: Attracted to females: 42 Bisexual: 2 Attracted to males: 5 Relationship: 31 of 49 Gender of partner: Heterosexual woman: 24 Homo/bisexual woman: 4 Homo/bisexual man: 1 AMAB transsexual: 2 Freq sexual activities: 1-2/month: 48% Several times weekly: 30% Sexual satisfaction: (Very) satisfied: 64% Use of clitoris during coitus: Touching: before: 12,8% after: 13% Stimulation: before: 34% after: 38,3% Use of vagina during coitus: Touching: before: 5,8% after: 11,4% Penetration: before: 25,5% after: 11,4% Results with vs without erection prosthesis: Freq of masturbation: (More than) weekly: 64,5% vs 30,8% Freq of easy arousal: Half of time: 45,2 vs 53,8 Orgasm through masturbation: (Almost) always: 67,9% vs 58,3% Orgasm through coitus: (Almost) always: 38,9% vs 37,5% Change in orgasmic feeling: 64,3% vs 58,3%
Wierckx et al 2011 Sexual desire^213^	Outcome measure	3	45 AFAB	22–54	Sexual desire, frequency of sexual activity and masturbation, sexual satisfaction with the current partner	The Dutch version of the Sexual Desire Inventory	Sexual desire after GAS: (Much) higher: 32 No change: 11 Decrease: 1 Nor willing to answer: 1 Satisfaction with sexual life: 64% Neutral 18% Unsatisfied: 18%
Zuckerman et al 2015 Penile prosthesis^223^	Outcome measure	4	15 AFAB 16 Cis men	M 35.6	Sexual activity	Chart review	Sexually active post-implant: 85%

AFAB = assigned female at birth (transmasculine individual, trans man); AMAB = assigned male at birth (transfeminine individual, trans woman); LoE = level of evidence (oxford centre for evidence-based medicine 2011); MRKHS = mayer rokitansky kuster hauser syndrome.

Five studies reported on postsurgical sensation, which was present in 86.3%.^216^, ^217^, ^218^, ^219^, ^220^ Most experienced partial sensation, either in the buried clitoral site,^219^ the shaft,^198^^,^ ^216^ the neo-urethra^198^ and sensation projecting to the thigh.^217^

Postsurgical orgasm was discussed in three studies. Garcia et al^221^ and Wierckx et al^26^ reported that 92% and 97.8% were able to reach orgasm, respectively, and Van de Grift et al^196^ found that orgasmic capacity increased in 18%, was unchanged in 52% and decreased in 26%.

Ten studies reported on the possibility of penetrative sex, which ranged between 38.8–85%.^205^^,^^211^^,^^212^^,^^214^^,^^216^^,^^217^^,^^219^^,^^220^^,^^222^^,^^223^

## Conclusions

This position statement provides healthcare providers with recommendations that may aid in decision making regarding GAS. Although findings may suggest positive outcomes regarding sexual wellbeing following vaginoplasty, mastectomy, metoidioplasty, and phalloplasty, the overall quality of evidence is still low and most recommendations of this position statement are Level of Evidence C.

Not only methods of data gathering and reporting vary, some forms of GAS are not studied at all when it comes to effects on sexual wellbeing. Therefore, we advise more research on the effects of orchiectomy-only, breast augmentation, vocal feminization surgery, facial feminization surgery and the removal of the female sexual organs on sexual wellbeing in trans individuals.

In trans individuals AMAB; breast augmentation is mostly studied with a focus on surgical techniques. Data on sensitivity, functioning and sexual wellbeing are lacking.

Next to the effects of FFS on sexual wellbeing, further research focusing on separate aspects of FFS is encouraged and necessary.

The majority of questionnaires that were applied in evaluating sexual activity after GAS in trans individuals AMAB, were validated for cis women- in heterosexual relationships, who engaged in penetrative sex- only. A substantial portion of aforementioned participants, however, were intimate with women, or did not have sexual relationships. Therefore, the development of specific questionnaires to evaluate the effect of GAS on the sexual wellbeing in trans individuals is needed.

In trans individuals AFAB; it is known that mastectomy is a viable option in improving gender incongruence, body image, psychological wellbeing, sexual wellbeing and overall quality of life. Evidence on sexual wellbeing after mastectomy is limited, focussing mainly on quality of sex life and sexual relationships.

In masculinizing genital GAS a metaidoioplasty provides a sensate neophallus with the possibility to void standing, erotic satisfaction, and high levels of postsurgical satisfaction, with minimal donor site morbidity. However, there is need for validated questionnaires that can measure functionality, aesthetic appearance and patient satisfaction, to improve objective conclusions.

Furthermore, the unique anatomy of the male genitalia and the absence of tissue engineering options, to replace the smooth muscle of the corpora cavernosa and spongiosum, complicate TPC. The absence of comparative studies hampers selection of preferential techniques. Functional outcomes and patient satisfaction are difficult to comment on because of the lack of validated questionnaires to assess these outcomes.

Long-term effects of GAMI and GAS should also be studied, where after consensus on cancer screening in trans individuals should be formed, especially in hormone sensitive cancers or organs.

Next to the lack of studies on the effects of GAS and GAMI on sexual wellbeing, research on the development of validated questionnaires and patient-reported outcome measures may aid in producing less heterogeneous data.

To conclude, heterogeneous methods of data gathering and reporting and missing data on sexual wellbeing after orchiectomy-only, vocal feminization surgery, facial feminization surgery and the removal of the female sexual organs further complicate the ability to draw robust conclusions, together with the lack of studies on the effects of GAS and GAMI on sexual wellbeing, emphasizing the need for future research. Future research on the development of validated questionnaires and patient-reported outcome measures may aid in producing less heterogeneous data. Researchers and clinicians alike should consider exchanging data and actively involve the transgender and gender-diverse community, in a bid to further improve not only surgical care, but trans-related care as a collective.

## Statement Of Authorship

Müjde Özer, Sahaand Poor Toulabi, Guy T'Sjoen, Joz Motmans: Conceptualization; Müjde Özer, Sahaand Poor Toulabi: Methodology; Müjde Özer, Sahaand Poor Toulabi: Investigation; All authors: Writing – Original Draft; All authors: Writing – Review & Editing; Guy T'Sjoen, Joz Motmans: Supervision.

## Appendix Supplementary materials

Image, application 1

Image, application 2

## References

[1] Verschuren JEA, Enzlin P, Dijkstra PU (2010). Chronic disease and sexuality: a generic conceptual framework. J Sex Res.

[2] Whipple B. (2008). The benefits of sexual expression on physical health. Sexologies.

[3] Meston CM, Frohlich PF. (2002). The psychobiology of sexual and gender identity disorders. Biol Psychiatry.

[4] Ein-Dor T, Hirschberger G. (2012). Sexual healing: daily diary evidence that sex relieves stress for men and women in satisfying relationships. J Soc Pers Relat.

[5] W P (2014).

[6] De Graaf H, Wijsen C. Seksuele Gezondheid in Nederland. 2017.

[7] Wilson EC, Chen Y-H, Arayasirikul S (2016). The impact of discrimination on the mental health of trans*female youth and the protective effect of parental support. AIDS Behav.

[8] Kerckhof ME, Kreukels BPC, Nieder TO (2019). Prevalence of sexual dysfunctions in transgender persons: results from the enigi follow-up study. J Sex Med.

[9] Wallwiener S, Strohmaier J, Wallwiener LM (2016). Sexual function is correlated with body image and partnership quality in female university students. J Sex Med.

[10] Corona G, Rastrelli G, Morgentaler A (2017). Meta-analysis of Results of testosterone therapy on sexual function based on international index of erectile function scores. Eur Urol.

[11] Santoro N, Worsley R, Miller KK (2016). Role of estrogens and estrogen-like compounds in female sexual function and dysfunction. J Sex Med.

[12] Davis SR, Davison SL, Donath S (2005). Circulating androgen levels and self-reported sexual function in women. Jama.

[13] Randolph JF, Zheng H, Avis NE (2015). Masturbation frequency and sexual function domains are associated with serum reproductive hormone levels across the menopausal transition. J Clin Endocrinol Metab.

[14] Bancroft J. (2005). The endocrinology of sexual arousal. J Endocrinol.

[15] Travison TG, Morley JE, Araujo AB (2006). The relationship between libido and testosterone levels in aging men. J Clin Endocrinol Metab.

[16] Turna B, Apaydin E, Semerci B (2005). Women with low libido: correlation of decreased androgen levels with female sexual function index. Int J Impot Res.

[17] Burrows LJ, Basha M, Goldstein AT. (2012). The effects of hormonal contraceptives on female sexuality: a review. J Sex Med.

[18] Davis SR, Moreau M, Kroll R (2008). Testosterone for low libido in postmenopausal women not taking estrogen. N Engl J Med.

[19] Redmond GP. (1999). Hormones and sexual function. Int J Fert Women's Med.

[20] Yassin AA, Saad F. (2007). Plasma levels of dihydrotestosterone remain in the normal range in men treated with long-acting parenteral testosterone undecanoate. Andrologia.

[21] Bradford NJ, Spencer K. (2020). Sexual pleasure in transgender and gender diverse individuals: an update on recent advances in the field. Curr Sex Health Rep.

[22] Bartolucci C, Gomez-Gil E, Salamero M (2015). Sexual quality of life in gender-dysphoric adults before genital sex reassignment surgery. J Sex Med.

[23] Cerwenka S, Nieder TO, Cohen-Kettenis P (2014). Sexual behavior of gender-dysphoric individuals before gender-confirming interventions: a European multicenter study. J Sex Marital Ther.

[24] Kronawitter D, Gooren LJ, Zollver H (2009). Effects of transdermal testosterone or oral dydrogesterone on hypoactive sexual desire disorder in transsexual women: results of a pilot study. Eur J Endocrinol.

[25] Kim GW, Kim SK, Jeong GW. (2016). Neural activation-based sexual orientation and its correlation with free testosterone level in postoperative female-to-male transsexuals: preliminary study with 3.0-T fMRI. Surg Radiol Anat.

[26] Wierckx K, Van Caenegem E, Elaut E (2011). Quality of life and sexual health after sex reassignment surgery in transsexual men. J Sex Med.

[27] Kim GW, Jeong GW. (2014). Neural mechanisms underlying sexual arousal in connection with sexual hormone levels: a comparative study of the postoperative male-to-female transsexuals and premenopausal and menopausal women. Neuroreport.

[28] Sturup GK. (1976). Male transsexuals: a long-term follow-up after sex reassignment operations. Acta Psychiatr Scand.

[29] Costantino A, Cerpolini S, Alvisi S (2013). A prospective study on sexual function and mood in female-to-male transsexuals during testosterone administration and after sex reassignment surgery. J Sex Marital Ther.

[30] Beckwith N, Reisner SL, Zaslow S (2017). Keuroghlian AS. factors associated with gender-affirming surgery and age of hormone therapy initiation among transgender adults. Transgend Health.

[31] Elaut E, Bogaert V, De Cuypere G (2010). Contribution of androgen receptor sensitivity to the relation between testosterone and sexual desire: an exploration in male-to-female transsexuals. J Endocrinol Invest.

[32] Kraemer B, Hobi S, Rufer M (2010). Partner relationship and sexuality of female-to-male transsexuals. Psychother Psychosom Med Psychol.

[33] Selvaggi G, Monstrey S, Ceulemans P (2007). Genital sensitivity after sex reassignment surgery in transsexual patients. Ann Plast Surg.

[34] de Cuypere G, Elaut E, Heylens G (2006). Long-term follow-up: psychosocial outcome of belgian transsexuals after sex reassignment surgery. Sexologies.

[35] Ristori J, Fisher AD, Cipriani A (2019). PS-01-005 The effect of hormonal treatment on sexual distress in transgender persons: a two-year follow-up study. J Sex Med.

[36] McCabe MP, Sharlip ID, Lewis R (2016). Risk factors for sexual dysfunction among women and men: a consensus statement from the fourth international consultation on sexual medicine 2015. J Sex Med.

[37] Fisher AD, Castellini G, Fanni E (2016). Cross-sex hormone treatment and psychobiological changes in transsexual persons: 2-years follow-up data. J Sex Med.

[38] van de Grift TC, Elfering L, Greijdanus M (2018). Subcutaneous mastectomy improves satisfaction with body and psychosocial function in trans men: findings of a cross-sectional study using the BODY-Q chest module. Plast Reconstr Surg.

[39] Nobili A, Glazebrook C, Arcelus J. (2018). Quality of life of treatment-seeking transgender adults: a systematic review and meta-analysis. Rev Endocr Metab Disord.

[40] Satterwhite T, Morrison SD, Ludwig DC (2017). Abstract: prospective quality of life outcomes after facial feminization surgery. Plast Reconstr Surg Glob Open.

[41] Byers ES, Rehman US. (2014). APA handbook of sexuality and psychology, Vol 1: Person-based approaches. APA handbooks in psychology®..

[42] Özer PT, Gijs Kreukels, Mullender (2019). Sexual wellbeing in gender incongruent individuals: towards sex positive definition and assessment. JSSM.

[43] Coleman E, Bockting W, Botzer M (2012). The World Professional Association for Transgender Health (WPATH). Standards of care for the health of transsexual, transgender, and gender nonconforming people.

[44] Saklad MMD. (1941). Grading of patients for surgical procedures. Anesthesiology.

[45] Owens WD. (2001). American society of anesthesiologists physical status classification system in not a risk classification system. Anesthesiology.

[46] Hoogendoorn JM, Simmermacher RK, Schellekens PP (2002). Adverse effects if smoking on healing of bones and soft tissues. Der Unfallchirurg.

[47] Bamgbade OA, Rutter TW, Nafiu OO, Dorje P. (2007). Postoperative complications in obese and nonobese patients. World J Surg.

[48] Berrington de Gonzalez A, Hartge P, Cerhan JR (2010). Body-mass index and mortality among 1.46 million white adults. N Engl J Med.

[49] Hembree WC, Cohen-Kettenis PT, Gooren L (2017). Endocrine treatment of gender-dysphoric/gender-incongruent persons: an endocrine society clinical practice guideline. Endocr Pract.

[50] Mattawanon N, Spencer JB (2018). Schirmer DA, 3rd, Tangpricha V. Fertility preservation options in transgender people: a review. Rev Endocr Metab Disord.

[51] van de Grift TC, Mullender MG, Bouman MB. (2018). Shared decision making in gender-affirming surgery. implications for research and standards of care. J Sex Med.

[52] Coleman E, Bockting W, Botzer M (2012). Standards of care for the health of transsexual, transgender, and gender-nonconforming people, version 7. Int J Transgenderism.

[53] Özer M, Pigot GLS, Bouman M-B (2018). Development of a decision aid for genital gender-affirming surgery in transmen. J Sex Med.

[54] Mokken SE, Özer M, van de Grift TC (2020). Evaluation of the decision aid for genital surgery in Transmen. J Sex Med.

[55] Lawrence AA. (2003). Factors associated with satisfaction or regret following male-to-female sex reassignment surgery. Arch Sex Behav.

[56] Jiang DD, Gallagher S, Burchill L (2019). Implementation of a pelvic floor physical therapy program for transgender women undergoing gender-affirming vaginoplasty. Obstetr Gynecol.

[57] Colebunders B, Brondeel S, D'Arpa S (2017). An update on the surgical treatment for transgender patients. Sex Med Rev.

[58] Wiepjes CM, Nota NM, de Blok CJM (2018). The Amsterdam cohort of gender dysphoria study (1972-2015): trends in prevalence, treatment, and regrets. J Sex Med.

[59] Beek TF, Kreukels BP, Cohen-Kettenis PT (2015). Partial treatment requests and underlying motives of applicants for gender affirming interventions. J Sex Med.

[60] Sorensen T. (1981). A follow-up study of operated transsexual females. Acta Psychiatr Scand.

[61] Lief HI, Hubschman L. (1993). Orgasm in the postoperative transsexual. Arch Sex Behav.

[62] van de Grift TC, Pigot GLS, Boudhan S (2017). A Longitudinal study of motivations before and psychosexual outcomes after genital gender-confirming surgery in transmen. J Sex Med.

[63] Cohen-Kettenis PT, van Goozen SH. (1997). Sex reassignment of adolescent transsexuals: a follow-up study. J Am Acad Child Adolesc Psychiatry.

[64] Smith YL, van Goozen SH, Cohen-Kettenis PT. (2001). Adolescents with gender identity disorder who were accepted or rejected for sex reassignment surgery: a prospective follow-up study. J Am Acad Child Adolesc Psychiatry.

[65] Jarolim L. (2000). Surgical conversion of genitalia in transsexual patients. BJU Int.

[66] Rakic Z, Starcevic V, Maric J, Kelin K. (1996). The outcome of sex reassignment surgery in Belgrade: 32 patients of both sexes. Arch Sex Behav.

[67] Tsoi WF. (1993). Follow-up study of transsexuals after sex-reassignment surgery. Singapore Med J.

[68] Lobato MI, Koff WJ, Manenti C (2006). Follow-up of sex reassignment surgery in transsexuals: a Brazilian cohort. Arch Sex Behav.

[69] Johansson A, Sundbom E, Hojerback T, Bodlund O. (2010). A five-year follow-up study of Swedish adults with gender identity disorder. Arch Sex Behav.

[70] Kuhn A, Santi A, Birkhauser M. (2011). Vaginal prolapse, pelvic floor function, and related symptoms 16 years after sex reassignment surgery in transsexuals. Fertil Steril.

[71] Lothstein LM. (1980). The postsurgical transsexual: empirical and theoretical considerations. Arch Sex Behav.

[72] Oefelein MG, Feng A, Scolieri MJ, Ricchiutti D, Resnick MI. (2000). Reassessment of the definition of castrate levels of testosterone: implications for clinical decision making. Urology.

[73] Rastrelli G, Guaraldi F, Reismann Y (2019). Testosterone Replacement therapy for sexual symptoms. Sex Med Rev.

[74] Karim RB, Hage JJ, Bouman FG (1995). Refinements of pre-, intra-, and postoperative care to prevent complications of vaginoplasty in male transsexuals. Ann Plast Surg.

[75] Nijhuis THJ, Özer M, van der Sluis WB (2020). The Bilateral pedicled epilated scrotal flap: a powerful adjunctive for creation of more neovaginal depth in penile inversion vaginoplasty. J Sex Med.

[76] Horbach SE, Bouman MB, Smit JM (2015). Outcome of vaginoplasty in male-to-female transgenders: a systematic review of surgical techniques. J Sex Med.

[77] Perovic SV, Stanojevic DS, Djordjevic ML. (2000). Vaginoplasty in male transsexuals using penile skin and a urethral flap. BJU Int.

[78] Selvaggi G, Bellringer J. (2011). Gender reassignment surgery: An overview. Nat Rev Urol.

[79] Buncamper ME, Honselaar JS, Bouman MB (2015). Aesthetic and functional outcomes of neovaginoplasty using penile skin in male-to-female transsexuals. J Sex Med.

[80] Zavlin D, Schaff J, Lelle JD (2018). Male-to-female sex reassignment surgery using the combined vaginoplasty technique: satisfaction of transgender patients with aesthetic, functional, and sexual outcomes. Aesthetic Plast Surg.

[81] Jiang D, Witten J, Berli J (2018). Does depth matter? factors affecting choice of vulvoplasty over vaginoplasty as gender-affirming genital surgery for transgender women. J Sex Med.

[82] van der Sluis WB, Steensma TD, Timmermans FW (2020). Gender-confirming vulvoplasty in transgender women in the netherlands: incidence, motivation analysis, and surgical outcomes. J Sex Med.

[83] Manrique OJ, Adabi K, Huang TC (2019). Assessment of pelvic floor anatomy for male-to-female vaginoplasty and the role of physical therapy on functional and patient-reported outcomes. Ann Plast Surg.

[84] Djordjevic ML, Stanojevic DS, Bizic MR. (2011). Rectosigmoid vaginoplasty: clinical experience and outcomes in 86 cases. J Sex Med.

[85] Lawrence AA. (2006). Patient-reported complications and functional outcomes of male-to-female sex reassignment surgery. Arch Sex Behav.

[86] Ramachandran VS, McGeoch PD. (2007). Occurrence of phantom genitalia after gender reassignment surgery. Med Hypotheses.

[87] Amend B, Seibold J, Toomey P (2013). Surgical reconstruction for male-to-female sex reassignment. Eur Urol.

[88] Blanchard R, Legault S, Lindsay WR. (1987). Vaginoplasty outcome in male-to-female transsexuals. J Sex Marital Ther.

[89] Bouman FG. (1988). Sex reassignment surgery in male to female transsexuals. Ann Plast Surg.

[90] Bouman MB, van der Sluis WB, van Woudenberg Hamstra LE (2016). Patient-reported esthetic and functional outcomes of primary total laparoscopic intestinal vaginoplasty in transgender women with penoscrotal hypoplasia. J Sex Med.

[91] Brotto LA, Gehring D, Klein C (2005). Psychophysiological and subjective sexual arousal to visual sexual stimuli in new women. J Psychosom Obstet Gynaecol.

[92] Buncamper ME, van der Sluis WB, de Vries M (2017). Penile inversion vaginoplasty with or without additional full-thickness skin graft: to graft or not to graft?. Plast Reconstr Surg.

[93] Cardoso da Silva D, Schwarz K, Fontanari AM (2016). WHOQOL-100 before and after sex reassignment surgery in brazilian male-to-female transsexual individuals. J Sex Med.

[94] Collyer F, Heal C. (2002). Patient satisfaction with sex re-assignment surgery in New South Wales, Australia. Aust J Prim Health.

[95] Eldh J. (1993). Construction of a neovagina with preservation of the glans penis as a clitoris in male transsexuals. Plast Reconstr Surg.

[96] Freundt I, Toolenaar TA, Huikeshoven FJ (1992). Jeekel H. A modified technique to create a neovagina with an isolated segment of sigmoid colon. Surg Gynecol Obstet.

[97] Giraldo F, Esteva I, Bergero T (2004). Corona glans clitoroplasty and urethropreputial vestibuloplasty in male-to-female transsexuals: the vulval aesthetic refinement by the Andalusia Gender Team. Plast Reconstr Surg.

[98] Goddard JC, Vickery RM, Qureshi A (2007). Feminizing genitoplasty in adult transsexuals: early and long-term surgical results. BJU Int.

[99] Hess J, Hess-Busch Y, Kronier J (2016). Modified preparation of the neurovascular bundle in male to female transgender patients. Urol Int.

[100] Imbimbo C, Verze P, Palmieri A (2009). A report from a single institute's 14-year experience in treatment of male-to-female transsexuals. J Sex Med.

[101] Jarolim L, Sedy J, Schmidt M (2009). Gender reassignment surgery in male-to-female transsexualism: A retrospective 3-month follow-up study with anatomical remarks. J Sex Med.

[102] Kanhai RC. (2016). Sensate vagina pedicled-spot for male-to-female transsexuals: the experience in the first 50 patients. Aesthetic Plast Surg.

[103] Karim RB, Hage JJ, Bouman FG (1991). The importance of near total resection of the corpus spongiosum and total resection of the corpora cavernosa in the surgery of male to female transsexuals. Ann Plast Surg.

[104] Kim SK, Park JH, Lee KC (2003). Long-term results in patients after rectosigmoid vaginoplasty. Plast Reconstr Surg.

[105] Kim SK, Park JW, Lim KR (2017). Is rectosigmoid vaginoplasty still useful?. Arch Plast Surg.

[106] Krege S, Bex A, Lummen G (2001). Male-to-female transsexualism: a technique, results and long-term follow-up in 66 patients. BJU Int.

[107] Lawrence AA. (2005). Sexuality before and after male-to-female sex reassignment surgery. Arch Sex Behav.

[108] LeBreton M, Courtois F, Journel NM (2017). Genital sensory detection thresholds and patient satisfaction with vaginoplasty in male-to-female transgender women. J Sex Med.

[109] Lindemalm G, Korlin D, Uddenberg N. (1986). Long-term follow-up of "sex change" in 13 male-to-female transsexuals. Arch Sex Behav.

[110] Lindemalm G, Korlin D, Uddenberg N. (1987). Prognostic factors vs. outcome in male-to-female transsexualism. A follow-up study of 13 cases. Acta Psychiatr Scand.

[111] Manrique OJ, Sabbagh MD, Ciudad P (2018). Gender-confirmation surgery using the pedicle transverse colon flap for vaginal reconstruction: a clinical outcome and sexual function evaluation study. Plast Reconstr Surg.

[112] Mate-Kole C, Freschi M, Robin A (1990). A controlled study of psychological and social change after surgical gender reassignment in selected male transsexuals. Br J Psychiatry.

[113] Morrison SD, Satterwhite T, Grant DW (2015). Long-term outcomes of rectosigmoid neocolporrhaphy in male-to-female gender reassignment surgery. Plast Reconstr Surg.

[114] Mukai Y, Watanabe T, Sugimoto M (2017). Vaginoplasty with a pudendal-groin flap in male-to-female transsexuals. Acta Med Okayama.

[115] Papadopulos NA, Lelle JD, Zavlin D (2020). Psychological pathologies and sexual orientation in transgender women undergoing gender confirming treatment. Ann Plast Surg.

[116] Papadopulos NA, Zavlin D, Lelle JD (2017). Combined vaginoplasty technique for male-to-female sex reassignment surgery: Operative approach and outcomes. J Plast Reconstr Aesthet Surg.

[117] Perovic SV, Stanojevic DS, Djordjevic ML. (2005). Vaginoplasty in male to female transsexuals using penile skin and urethral flap. Int J Transgenderism.

[118] Raigosa M, Avvedimento S, Yoon TS (2015). Male-to-female genital reassignment surgery: a retrospective review of surgical technique and complications in 60 patients. J Sex Med.

[119] Reed HM, Yanes RE, Delto JC (2015). Non-grafted vaginal depth augmentation for transgender atresia, our experience and survey of related procedures. Aesthetic Plast Surg.

[120] Rehman J, Lazer S, Benet AE (1999). The reported sex and surgery satisfactions of 28 postoperative male-to-female transsexual patients. Arch Sex Behav.

[121] Rehman J, Melman A. (1999). Formation of neoclitoris from glans penis by reduction glansplasty with preservation of neurovascular bundle in male-to-female gender surgery: functional and cosmetic outcome. J Urol.

[122] Salgado CJ, Nugent A, Kuhn J (2018). Primary sigmoid vaginoplasty in transwomen: technique and outcomes. Biomed Res Int.

[123] Schroder M, Carroll RA. (1999). New women: sexological outcomes of male-to-female gender reassignment surgery. J Sex Educ Ther.

[124] Seyed-Forootan K, Karimi H (2018). Seyed-Forootan NS. autologous fibroblast-seeded amnion for reconstruction of neo-vagina in male-to-female reassignment surgery. Aesthetic Plast Surg.

[125] Sigurjonsson H, Mollermark C, Rinder J (2017). Long-term sensitivity and patient-reported functionality of the neoclitoris after gender reassignment surgery. J Sex Med.

[126] Soli M, Brunocilla E, Bertaccini A (2008). Male to female gender reassignment: modified surgical technique for creating the neoclitoris and mons veneris. J Sex Med.

[127] Stanojevic DS, Djordjevic ML, Milosevic A (2007). Sacrospinous ligament fixation for neovaginal prolapse prevention in male-to-female surgery. Urology.

[128] Stein M, Tiefer L, Melman A. (1990). Followup observations of operated male-to-female transsexuals. J Urol.

[129] Tavakkoli Tabassi K, Djavan B, Hosseini J (2014). Fold-back perineoscrotal flap plus penile inversion vaginoplasty for male-to-female gender reassignment surgery in circumcised subjects. Eur J Plast Surg.

[130] Thalaivirithan BM, Sethu M, Ramachandran DK (2018). Application of embryonic equivalents in male-to-female sex reassignment surgery. Indian J Plast Surg.

[131] Toolenaar TA, Freundt I, Huikeshoven FJ (1993). The occurrence of diversion colitis in patients with a sigmoid neovagina. Hum Pathol.

[132] van der Sluis WB, Bouman MB, de Boer NK (2016). Long-term follow-up of transgender women after secondary intestinal vaginoplasty. J Sex Med.

[133] van der Sluis WB, Neefjes-Borst EA, Bouman MB (2016). Morphological spectrum of neovaginitis in autologous sigmoid transplant patients. Histopathology.

[134] Wagner S, Greco F, Hoda MR (2010). Male-to-female transsexualism: technique, results and 3-year follow-up in 50 patients. Urol Int.

[135] Weyers S, Elaut E, De Sutter P (2009). Long-term assessment of the physical, mental, and sexual health among transsexual women. J Sex Med.

[136] Wu JX, Li B, Li WZ (2009). Laparoscopic vaginal reconstruction using an ileal segment. Int J Gynaecol Obstet.

[137] Zavlin D, Wassersug RJ, Chegireddy V (2019). Age-related differences for male-to-female transgender patients undergoing gender-affirming surgery. Sex Med.

[138] Cocci A, Rosi F, Frediani D (2019). Male-to-Female (MtoF) gender affirming surgery: Modified surgical approach for the glans reconfiguration in the neoclitoris (M-shape neoclitorolabioplasty). Arch Ital Urol Androl.

[139] di Summa PG, Watfa W, Krahenbuhl S (2019). Colic-based transplant in sexual reassignment surgery: functional outcomes and complications in 43 consecutive patients. J Sex Med.

[140] Hess J, Henkel A, Bohr J (2018). Sexuality after male-to-female gender affirmation surgery. Biomed Res Int.

[141] Watanyusakul S. (2019). Vaginoplasty modifications to improve vulvar aesthetics. Urol Clin North Am.

[142] Elaut E, De Cuypere G, De Sutter P (2008). Hypoactive sexual desire in transsexual women: prevalence and association with testosterone levels. Eur J Endocrinol.

[143] Freund K, Langevin R, Zajac Y. (1974). The transsexual syndrome in homosexual males. J Nerv Ment Dis.

[144] Bentler PM. (1976). A typology of transsexualism: gender identity theory and data. Arch Sex Behav.

[145] De Cuypere G, T'Sjoen G, Beerten R (2005). Sexual and physical health after sex reassignment surgery. Arch Sex Behav.

[146] Djordjevic ML, Bizic MR. (2013). Comparison of two different methods for urethral lengthening in female to male (metoidioplasty) surgery. J Sex Med.

[147] Lawrence AA, Latty EM, Chivers ML (2005). Measurement of sexual arousal in postoperative male-to-female transsexuals using vaginal photoplethysmography. Arch Sex Behav.

[148] Monstrey S, Selvaggi G, Ceulemans P (2008). Chest-wall contouring surgery in female-to-male transsexuals: a new algorithm. Plast Reconstr Surg.

[149] Weigert R, Frison E, Sessiecq Q (2013). Patient satisfaction with breasts and psychosocial, sexual, and physical well-being after breast augmentation in male-to-female transsexuals. Plast Reconstr Surg.

[150] Kanhai RC, Hage JJ, Karim RB (1999). Exceptional presenting conditions and outcome of augmentation mammaplasty in male-to-female transsexuals. Ann Plast Surg.

[151] Laub DR, Fisk N. (1974). A rehabilitation program for gender dysphoria syndrome by surgical sex change. Plast Reconstr Surg.

[152] Coon D, Lee E, Fischer B (2020). Breast augmentation in the transfemale patient: comprehensive principles for planning and obtaining ideal results. Plast Reconstr Surg.

[153] Selvaggi G. (2020). Discussion: breast augmentation in the transfemale patient: comprehensive principles for planning and obtaining ideal results. Plast Reconstr Surg.

[154] Fitzal F, Turner SD, Kenner L. (2019). Is breast implant-associated anaplastic large cell lymphoma a hazard of breast implant surgery?. Open Biol.

[155] de Blok CJM, Wiepjes CM, Nota NM (2019). Breast cancer risk in transgender people receiving hormone treatment: nationwide cohort study in the Netherlands. BMJ.

[156] Claes KEY, D'Arpa S, Monstrey SJ. (2018). Chest surgery for transgender and gender nonconforming individuals. Clin Plast Surg.

[157] Hancock AB, Krissinger J, Owen K. (2011). Voice perceptions and quality of life of transgender people. J Voice.

[158] Capitán L, Gutiérrez Santamaría J, Simon D (2020). Facial gender confirmation surgery: a protocol for diagnosis, surgical planning, and postoperative management. Plast Reconstr Surg.

[159] Safa B, Lin WC, Salim AM (2019). Current concepts in feminizing gender surgery. Plast Reconstr Surg.

[160] Capitán L SD, Capitán-Cañadas F, Ferrando CA (2020). Comprehensive care of the transgender patient.

[161] Mareckova K, Weinbrand Z, Chakravarty MM (2011). Testosterone-mediated sex differences in the face shape during adolescence: subjective impressions and objective features. Horm Behav.

[162] Capitan L, Simon D, Berli JU (2017). Facial gender confirmation surgery: a new nomenclature. Plast Reconstr Surg.

[163] Ettner R, Monstrey S, Coleman E. Principles of transgender medicine and surgery. 2016.

[164] Capitan L, Simon D, Meyer T (2017). Facial feminization surgery: simultaneous hair transplant during forehead reconstruction. Plast Reconstr Surg.

[165] Capitan L, Simon D, Bailon C (2019). The upper third in facial gender confirmation surgery: forehead and hairline. J Craniofac Surg.

[166] Bellinga RJ, Capitan L, Simon D, Tenorio T (2017). Technical and clinical considerations for facial feminization surgery with rhinoplasty and related procedures. JAMA Facial Plast Surg.

[167] Prein J, Assael LA, Klotch DW (2014).

[168] Ehrenfeld M, Manson PN, Prein J (2012).

[169] Becking AG, Tuinzing DB, Hage JJ (2007). Transgender feminization of the facial skeleton. Clin Plast Surg.

[170] Balaji SM. (2016). Facial feminization - Surgical modification for Indian, European and African faces. Ann Maxillofac Surg.

[171] Raffaini M, Magri AS, Agostini T. (2016). Full facial feminization surgery: patient satisfaction assessment based on 180 procedures involving 33 consecutive patients. Plast Reconstr Surg.

[172] Ousterhout D. Feminization of the chin: a review of 485 consecutive cases. In: International Society of Craniofacial S, International C, Salyer KE, editors. Craniofacial surgery: proceedings of the Tenth International Congress of the International Society of Craniofacial Surgery, Monterey, California (USA), 2003 Bologna, Italy: Medimond International Proceedings; 2003.

[173] Ousterhout D. Feminization of the mandibular body: a review of 688 consecutive cases. In: International Society of Craniofacial S, International C, David DJ, editors. Craniofacial surgery: proceedings of the Eleventh International Congress of the International Society of Craniofacial Surgery: Coolum, Queensland, Australia, 2005. Bologna, Italy: Medimond International Proceedings; 2005.

[174] Yahalom R, Blinder D, Nadel S. (2015). Facial femalization in transgenders. Refu’at ha-peh veha-shinayim (1993).

[175] Boucher F, Gleizal A, Mojallal A (2017). Facial feminization surgery - middle and inferior thirds. Ann Chir Plast Esthet.

[176] Mommaerts MY, Voisin C, Joshi Otero J (2019). Mandibular feminization osteotomy-preliminary results. Int J Oral Maxillofac Surg.

[177] Morrison SD, Satterwhite T. (2019). Lower jaw recontouring in facial gender-affirming surgery. Facial Plast Surg Clin North Am.

[178] Deschamps-Braly J. (2019). Feminization of the chin: genioplasty using osteotomies. Facial Plastic Surg Clin North Am.

[179] Smith YL, Van Goozen SH, Kuiper AJ (2005). Sex reassignment: outcomes and predictors of treatment for adolescent and adult transsexuals. Psychol Med.

[180] van de Grift TC, Elaut E, Cerwenka SC (2017). Effects of medical interventions on gender dysphoria and body image: a follow-up study. Psychosom Med.

[181] Cregten-Escobar P, Bouman MB, Buncamper ME (2012). Subcutaneous mastectomy in female-to-male transsexuals: a retrospective cohort-analysis of 202 patients. J Sex Med.

[182] Griepsma M, de Roy van Zuidewijn DBW, Grond AJK (2014). Residual breast tissue after mastectomy: how often and where is it located?. Ann Surg Oncol.

[183] Esmonde N, Heston A, Jedrzejewski B (2019). What is "Nonbinary" and what do i need to know? a primer for surgeons providing chest surgery for transgender patients. Aesthet Surg J.

[184] Poudrier G, Nolan IT, Cook TE (2019). Assessing quality of life and patient-reported satisfaction with masculinizing top surgery: a mixed-methods descriptive survey study. Plast Reconstr Surg.

[185] Al-Tamimi M, Pigot GL, van der Sluis WB (2018). Colpectomy significantly reduces the risk of urethral fistula formation after urethral lengthening in transgender men undergoing genital gender affirming surgery. J Urol.

[186] Bizic MR, Stojanovic B, Joksic I (2019). Metoidioplasty. Urol Clin North Am.

[187] Stojanovic B, Bizic M, Bencic M (2017). One-stage gender-confirmation surgery as a viable surgical procedure for female-to-male transsexuals. J Sex Med.

[188] Perovic SV, Djordjevic ML. (2003). Metoidioplasty: a variant of phalloplasty in female transsexuals. BJU Int.

[189] Djordjevic ML, Stanojevic D, Bizic M (2009). Metoidioplasty as a single stage sex reassignment surgery in female transsexuals: Belgrade experience. J Sex Med.

[190] Durfee R RW., Laub DR, Gandy P (1974). Proceedings of the second Interdisciplinary Symposium on Gender Dysphoria Syndrome.

[191] Djordjevic ML, Stojanovic B, Bizic M. (2019). Metoidioplasty: techniques and outcomes. Transl Androl Urol.

[192] Selvaggi G, Hoebeke P, Ceulemans P (2009). Scrotal reconstruction in female-to-male transsexuals: a novel scrotoplasty. Plast Reconstr Surg.

[193] Djordjevic ML. (2018). Novel surgical techniques in female to male gender confirming surgery. Transl Androl Urol.

[194] Nikolavsky D, Hughes M, Zhao LC. (2018). Urologic complications after phalloplasty or metoidioplasty. Clin Plast Surg.

[195] Takamatsu A, Harashina T. (2009). Labial ring flap: a new flap for metaidoioplasty in female-to-male transsexuals. J Plast Reconstr Aesthet Surg.

[196] van de Grift TC, Pigot GLS, Kreukels BPC (2019). Transmen's experienced sexuality and genital gender-affirming surgery: findings from a clinical follow-up study. J Sex Marital Ther.

[197] Vukadinovic V, Stojanovic B, Majstorovic M (2014). The role of clitoral anatomy in female to male sex reassignment surgery. Sci World J.

[198] Garaffa G, Christopher NA, Ralph DJ. (2010). Total phallic reconstruction in female-to-male transsexuals. Eur Urol.

[199] Falcone M, Garaffa G, Raheem A (2016). Total phallic reconstruction using the radial artery based forearm free flap after traumatic penile amputation. J Sex Med.

[200] Garaffa G, Raheem AA, Christopher NA (2009). Total phallic reconstruction after penile amputation for carcinoma. BJU Int.

[201] Garaffa G, Spilotros M, Christopher NA (2014). Total phallic reconstruction using radial artery based forearm free flap phalloplasty in patients with epispadias-exstrophy complex. J Urol.

[202] Monstrey S, Hoebeke P, Selvaggi G (2009). Penile reconstruction: is the radial forearm flap really the standard technique?. Plast Reconstr Surg.

[203] Selvaggi G, Monstrey S, Hoebeke P (2006). Donor-site morbidity of the radial forearm free flap after 125 phalloplasties in gender identity disorder. Plast Reconstr Surg.

[204] Van Caenegem E, Verhaeghe E, Taes Y (2013). Long-Term evaluation of donor-site morbidity after radial forearm flap phalloplasty for transsexual men. J Sex Med.

[205] Bettocchi C, Ralph DJ, Pryor JP. (2005). Pedicled pubic phalloplasty in females with gender dysphoria. BJU Int.

[206] Garaffa G, Ralph DJ, Christopher N. (2010). Total urethral construction with the radial artery-based forearm free flap in the transsexual. BJU Int.

[207] van der Sluis WB, Smit JM, Pigot GLS (2017). Double flap phalloplasty in transgender men: surgical technique and outcome of pedicled anterolateral thigh flap phalloplasty combined with radial forearm free flap urethral reconstruction. Microsurgery.

[208] Felici N, Felici A. (2006). A new phalloplasty technique: the free anterolateral thigh flap phalloplasty. J Plast Reconstr Aesthet Surg.

[209] D'Arpa S, Claes K, Lumen N (2019). Urethral reconstruction in anterolateral thigh flap phalloplasty: a 93-case experience. Plast Reconstr Surg.

[210] Perovic SV, Djinovic R, Bumbasirevic M (2007). Total phalloplasty using a musculocutaneous latissimus dorsi flap. BJU Int.

[211] Ranno R, Hyza P, Vesely J (2007). An objective evaluation of contraction power of neo-phallus reconstructed with free re-innervated LD in female-to-male transsexuals. Acta Chir Plast.

[212] Ranno R, Vesely J, Hyza P (2007). Neo-phalloplasty with re-innervated latissimus dorsi free flap: a functional study of a novel technique. Acta Chir Plast.

[213] Wierckx K, Elaut E, Van Caenegem E (2011). Sexual desire in female-to-male transsexual persons: exploration of the role of testosterone administration. Eur J Endocrinol.

[214] Papadopulos NA, Schaff J, Biemer E. (2001). Usefulness of free sensate osteofasciocutaneous forearm and fibula flaps for neophallus construction. J Reconstr Microsurg.

[215] Schaff J, Papadopulos NA. (2009). A new protocol for complete phalloplasty with free sensate and prelaminated osteofasciocutaneous flaps: experience in 37 patients. Microsurgery.

[216] Leriche A, Timsit MO, Morel-Journel N (2008). Long-term outcome of forearm flee-flap phalloplasty in the treatment of transsexualism. BJU Int.

[217] Vesely J, Hyza P, Ranno R (2007). New technique of total phalloplasty with reinnervated latissimus dorsi myocutaneous free flap in female-to-male transsexuals. Ann Plast Surg.

[218] Garaffa G, Christopher NA, Ralph DJ. (2010). Total phallic reconstruction in female-to-male transsexuals. Eur Urol.

[219] Fang RH, Kao YS, Ma S (1999). Phalloplasty in female-to-male transsexuals using free radial osteocutaneous flap: A series of 22 cases. Br J Plast Surg.

[220] Falcone M, Garaffa G, Gillo A (2018). Outcomes of inflatable penile prosthesis insertion in 247 patients completing female to male gender reassignment surgery. BJU Int.

[221] Garcia MM, Christopher NA, De Luca F, Spilotros M, Ralph DJ. (2014). Overall satisfaction, sexual function, and the durability of neophallus dimensions following staged female to male genital gender confirming surgery: the Institute of Urology, London U.K. experience. Transl Androl Urol.

[222] Noe JM, Birdsell D, Laub DR. (1974). The surgical construction of male genitalia for the female to male transsexual. Plast Reconstr Surg.

[223] Zuckerman JM, Smentkowski K, Gilbert D (2015). Penile prosthesis implantation in patients with a history of total phallic construction. J Sex Med.

[224] Soares AB, Franco FF, Rosim ET (2011). Mastopexia com uso de implantes associados a retalho de músculo peitoral maior: técnica utilizada na Disciplina de Cirurgia Plástica da Unicamp. Rev Bras Cir Plást.

[225] McGhee DE, Steele JR. (2011). Breast volume and bra size. Int J Cloth Sci Technol.

[226] McGhee DE, Steele JR. (2006). How do respiratory state and measurement method affect bra size calculations?. Br J Sports Med.

