# Supplementary material for: ESSM Position Statement “Sexual Wellbeing After Gender Affirming Surgery”
Source: Sex Med. 2021 Dec 28;10(1):100471. doi: 10.1016/j.esxm.2021.100471 (PMC8847816; doi:10.1016/j.esxm.2021.100471)
Supplement: Supplementary file 1 [file mmc1.docx]

## 2.1.0 Literature search

A search strategy was developed with aid from a research librarian at the VU University Medical center. Relevant papers were sourced from the Medline, EMBASE, and Cochrane Library electronic databases from May 2017 untilApril 2020 The keywords and index terms, including applicable MeSH and Entree terms, were applied to each database. Search terms were generated under two broad headings referring to subject matter: gender incongruence and sexual wellbeing. This was done to have a wide scope on this subject, later the scope was narrowed down to sexual wellbeing after GAS.

The following MeSH terms were applied to Medline searches: sex reassignment procedures; gender dysphoria; transgender persons; transsexualism; gender incongruence; gender affirming; trans women; trans men; sexual behaviour; coitus, courtship; masturbation; orgasm; dyspareunia; intercourse; copulation; penetration; lubrication; sexual; sensation; pain; arousal; desire; pleasure; satisfaction; dysfunction; wellbeing; relation; behaviour; activity and quality of sex life were applicable for MeSH terminology. Reference lists were checked for relevant articles. Experts in the field were contacted for relevant articles. Articles with publication dates prior to July 2019 were considered.

### 2.1.1. Eligibility and inclusion

Inclusion and exclusion criteria were pre-specified (Table 1). Studies that assessed sexual topics in gender incongruent individuals in any stage of transition- irrespective of any medical, social or psychological interventions- were considered. Studies with fewer than 10 participants were excluded, in efforts to minimizeheterogeneity. Studies reporting on cisgender individuals only were excluded. Broad eligibility criteria were designed to reduce the risk of selection bias. No restrictions were imposed regarding age of participants, duration of follow-up, time of and method of data collection, randomization or blinding, region or time period of the publication. Our search was limited to English language publications only. Commentar­ies, conference abstracts, reviews and published abstracts were excluded.

Table 1 Pre-specified inclusion and exclusion criteria

| Inclusion criteria | Exclusion criteria |
| --- | --- |
| Gender-incongruent population | Non-gender-incongruent samples (LGB, MSM) |
| All transition phases | Case studies or series (n<10) |
| Sexual wellbeing | Studies on risk behaviour |
| Sexual topics | Non-English language |
|  | Reviews, abstracts, commentaries |

2.1.2 Study selection

Results of the literature search were screened for eligibility on title and abstract bytwo senior authors (MO, SPH) independently, through the web-based programme *Covidence.*(64) Conflicts were resolved and the same authors assessed further eligibilityafter reading the full text manuscripts. (put is as supplementary figure Fig 1-?) flow chart Covidence).

### 2.1.3 Data extraction

Data regarding sexual wellbeing was extracted into a standardized template, constructed by the two senior authors. Author’s name, year of publication, study design, intervention, sample size, age range, data related to sexuality and types of GAS were extracted from the articles.

Figure 1 flowchart Covidence


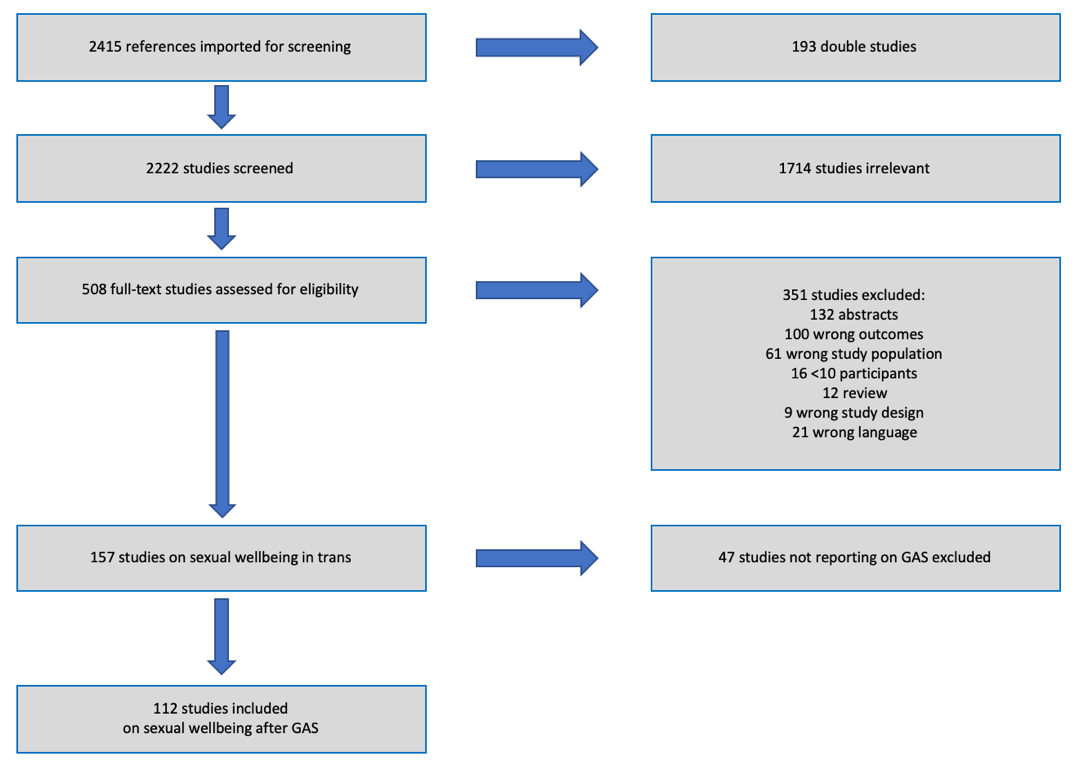


Figure 2 Studies on sexual wellbeing after GAS divided by type of surgical treatment


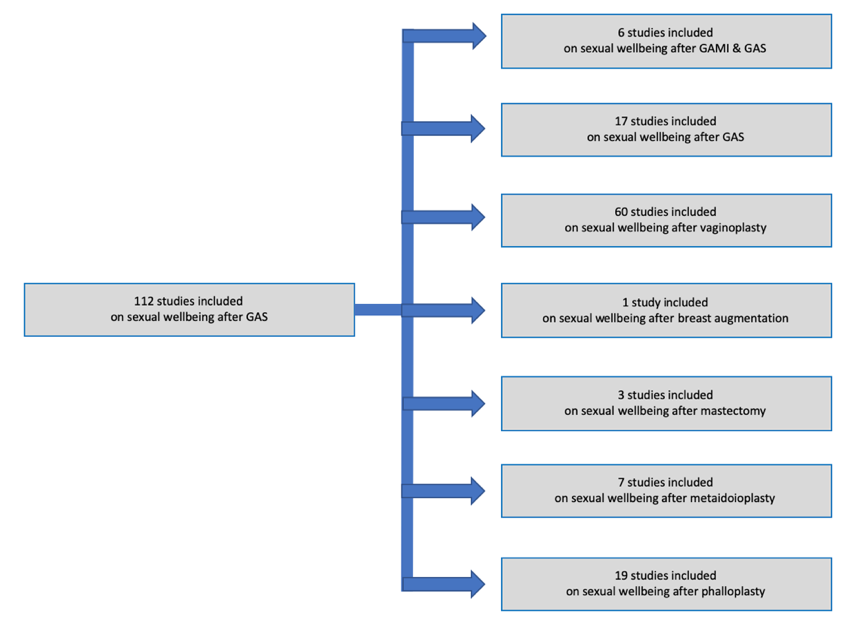


Our search on sexual wellbeing in trans individuals following GAS resulted in 110 studies, reporting on sexual wellbeing after Gender Affirming Surgery (GAS), vaginoplasty, mastectomy, metaidoioplasty, phalloplasty and GAMI together with GAS. We could not find literature reporting on sexual wellbeing after breast augmentation, vocal surgery and facial surgery. Two studies reported on both metaidoioplasty and phalloplasty, comparing both, that is why the sum of the studies in figure 2 (put it as supplementary Figure) is 112.(65, 66)

## 2.2.0 Assessment of quality of evidence

Strength of evidence was graded by the two authors independently, based on the Oxford Centre for Evidence-Based Medicine (OCEBM) levels of evidence criteria.(67)Discrepancies were settled through discussion.
