# Supplementary material for: ESSM Position Statement “Sexual Wellbeing After Gender Affirming Surgery”
Source: Sex Med. 2021 Dec 28;10(1):100471. doi: 10.1016/j.esxm.2021.100471 (PMC8847816; doi:10.1016/j.esxm.2021.100471)
Supplement: Supplementary file 2 [file mmc2.docx]

**Supplement 2. Postsurgical Sexual Wellbeing**

**Sexual Wellbeing following Gender Affirming Surgery**

| Study | Design |  | Sample size | Age range | Sexual Topics | Methods/Tools | Outcomes regarding Sexual Wellbeing |
| --- | --- | --- | --- | --- | --- | --- | --- |
| Cohen-Kettenis et al 1997 Sexual Reassignment of Adolescent | Follow-up study | 4 | 9 AMAB 20 AFAB | 19-27 | Sexual activity, intercourse, orgasm, relationship, masturbation, satisfaction with sex life | Semi-structured interview | Sexually active: n=13 Orgasm: 77%  No partner at time of interview or had never had one: 57% Masturbation: 50% < 1/mo or never, 43% >1/mo; AMAB decrease in frequency, AFAB no change or increase Sexual satisfaction: 71% satisfied, 14% neutral view, 14% were dissatisfied |
| Jarolim et al 2000 Surgical conversion of genitalia | Outcome meassure | 4 | 52 AMAB 30 AFAB | 17-51 | Lubrication, orgasm, clitoral activity | Chart review | Vaginoplasty (n=29): Sexual stimulation leads to production of urethral secretions which served as natural lubrication. Enabled coitus with orgasm  Metoidioplasty (n=28): Preserved erotogenic clitoral activity |
| Johansson et al 2010 A Five-Year Follow-Up | Follow-up study | 4 | 25 AMAB 17 AFAB | 18-60 | Relationships, sexual orientation, sex life, sexual impairment | Semi-structured interview, self-developed questionnaire | Sexual impairment after GAS: 5% AMAB Sex life: 70% better, 25% unchanged, 5% worsened Partner relations: 62% better, 30% unchanged, 8% worsened |
| Kuhn et al 2011 Vaginal prolapse, pelvic floor | Follow-up study | 4 | 52 AMAB 3 AFAB | No data | Sexual activity: satisfaction, frequency | Sheffield prolapse questionnaire | Stable relationship: 71%  75% considered sex life to interfere to some degree with enjoyment of life (better and/or worse) Most were sexually satisfied and had sex on a regular basis |
| Lief et al 1993 Orgasm in the postoperative | Outcome meassure | 4 | 14 AMAB 9 AFAB | 27-63 | Frequency of sex (not restricted to coitus, orgasmic capacity, reasons for anorgasmia, sexual satisfaction, sexual preference | Self-developed questionnaire | AMAB Orgasm: 10 anorgasmic, 10/14 Orgasmic before, 8 lost capacity, 4/14 anorgastic before, 2 gained capacity  Reasons for anorgasmia: dissatisfied with vaginal depth or cosmesis  Frequency of sex: 75% increase Sexual satisfaction: 9/14 increase in satisfaction  AFAB Orgasm: 7/9 Orgasmic, through masturbation, intercourse, and oral sex. 6 anorgasmic before: 4 gained capacity Reasons for anorgasmia: dissatisfied with masculinity level of body  Frequency of sex: 100% increase Same people reporting being orgasmic report being sexually satisfied.  Sexual satisfaction: 6/9 increase in satisfaction |
| Laub et al 1974 A rehabilitation program | Outcome meassure | 4 | 45 AMAB 24 AFAB | No data | Relationships, intercourse, orgasm, prostitution | Chart review | Improvement of sexual adjustment: 31/34,  no change in 3/34 (AMAB) |
| Lobato et al 2006 Follow-Up of Sex Reassignment | Follow-up study | 4 | 18 AMAB 1 AFAB | 18-47 | Satisfaction with sexual experience, partnerships, and relationship with family members, sexually active, frequency of sex, pleasurability of sex. | Self-developed questionnaire | Sexually active: 95% More frequent sex: 64.7%  Pleasurability of sex: 83.3%  Partnership Initiating and maintaining relationship easier post SRS: 64.7% Relationship pre-op: 52.6% Relationship post-op: 73.7% Satisfaction with sexual experience poor or very poor post-op: 11.2% Improvement of sexual experience post-op: 83.3% |
| Lothstein et al 1980 The postsurgical transsexual | Group comparison / Follow-up study | 4 | 8 AMAB 6 AFAB | 21-42 | Sexual adjustment and functioning, improvement sex life,  sexual activity, number of sexual partners, orgasm, sexual behaviour, partnering | Self-developed questionnaire | AMAB Improvement sex life 67% Sexual activity & number of sexual partners: no increase  Orgasm: 2 ejaculatory sensations Sexual behaviour: more flexible and spontaneous (engaging more frequently in oral, anal, and vaginal intercourse) Partnering: tended to seek out new partners, 64% no relationship   AFAB Improvement sex life 67% Sexual activity & number of sexual partners: no increase  Partnering: kept the same partners, 64% no relationship |
| Rakic et al 1996  The outcome of sex reassignment | Follow-up study | 4 | 22 AMAB 10 AFAB | 19-47 | Orgasm, number of sex partners, sexual activity, satisfaction with relationships | Self-developed questionnaire: 'Adjustment to Sex Reassignment Surgery' | Relationship satisfaction: 87% Sexual partner: AMAB 23%, AFAB 80% Several sexual partners: AMAB 50% Orgasm: 50% |
| Selvaggi et al 2007  Genital sensitivity after sex reassignment | Outcome meassure | 4 | 30 AMAB 27 AFAB | No data | Orgasm, sensitivity | Interview and physical examination: Semmes-Weinstein, vibration tests (biothesiometer) | The reconstructed genitalia obtain tactile and erogenous sensitivity Orgasm: AMAB 85%, AFAB 100% |
| Smith et al 2001 Adolescents With Gender Identity Disorder | Group comparison / Follow-up study | 4 | 7 AMAB 13 AFAB | 15-19 | Orgasm, relationship status, sexual orientation, intercourse, sexual functioning | Self-developed questionnaire, Utrecht Gender Dysphoria Scale (UGS), Body Image Scale (BIS) | Sexual partner: 10 patients Satisfaction with sex life: 1 FM was dissatisfied (could not have intercourse with a “normal” penis)  Several AFAB: difficult to live without a penis know their potential sexual partner well. Masturbation: AMAB -->decrease in masturbation frequency, AFAB--> increase or no change.  Orgasms: 69% Sexual orientation: stayed compatible |
| Smith et al 2005 Transsexual subtypes | Group comparison / Follow-up study | 4 | 113 AMAB 74 AFAB | Mean between 23.8-36.8 | Sexual arousal, sensitivity, sexual orientation | Biographical Questionnaire for Transsexuals, Body Image Scale (BIS), Utrecht Gender Dysphoria Scale (UGS) | (Have been) married homosexual < heterosexual Sexually aroused while cross-dressing homosexual < heterosexual |
| Sorensen 1981 A follow-up study | Outcome meassure | 4 | 8 AFAB | 30-60 | Intercourse, sexual satisfaction, masturbation, orgasm, sensitivity, ability to perform intercourse, importance of sexual activity, pain | Structured interview | Sexual partner: all patients Sexual satisfaction: only from clitoris (all); 5 satisfactory, 3 unsatisfactory Masturbation: all, >2 a week Orgasm: all Intercourse: 2 with phalloplasty with rib, but with pain, 6 with dildo Importance of sexual activity: essential in a life with a partner to all Pain: during intercourse in the 2 patients with phalloplasty from rib |
| Sorensen et al 1982 Male and female transsexualism | Outcome meassure | 4 | 29 AMAB 8 AFAB | 20-55 | Orgasm, sexual preference, sexual interest, sexual activity, intercourse, relationship status | Chart review | AMAB attracted to males (passive role) 93% low sexual interest 17% no sexual experience 58% no or low coital activity 2/3 no genital satisfaction  AFAB all attracted to women and previous homosexual relations Experience genital satisfaction by coitus and masturbation 5 of 8 also heterosexual experience (1 genital satisfaction) |
| Tsoi et al 1993 Follow-up study of | Outcome meassure | 4 | 45 AMAB 36 AFAB | 20-36 | Satisfaction with: sexual activities, organ functioning, sex status, sex organ | Semi-structured interview, self-developed questionnaire | Good or satisfactory sexual activity:  MtF 64% vs FtM 61%  Good or satisfactory sex organ function: MtF 91% vs FtM 39%  Good or satisfactory sex status: MtF 95% vs FtM 81% Good or satisfactory sex organ: MtF 91% vs FtM 39% |
| van de Grift et al 2017 A longitudinal study | Follow-up study | 4 | 21 AFAB | Mean 40.1 y | Sexual activity (type of activity), sexual preference and change in preference, partnering, sexual satisfaction | Self-developed questionnaire, chart review, Body Image Scale (BIS) | Female partner 63,2% Male partner 10,5% Single 26,3% Sexual function: metoidioplasty higher sexual satisfaction  Postop sexual activity: more masturbation and sexual activity, genitals more freq used (31% vs 78%) More pleasure, confidence, passive role Changed sexual orientation (“exclusively to men” to “primarily to women”).  Grade for sex life: 5.5 of 10 (SD 2.6) à “impossibility to penetrate/no erection prosthesis”, “not sexually active”, “penile size/sensation” and “partner issues” |
| Wierckx et al 2011 Quality of life and | Follow-up study | 4 | 49 AFAB | 22-54 | Relationship status, sexual preference, sexual activities (frequency, type of activity, pain), sexual wellbeing, masturbation, sexual satisfaction, orgasm, arousal | Self-developed questionnaire | Treatment phase differed within the group Relationship 63,3% Attracted to females 85,7% Partner: heterosexual woman 77,4% Frequency of sex: Never 22,2% 1-2/month 48,1% Several times a week 29,6% Sex satisfaction: (very satisfied) 64,2%  Erection prosthesis (n=32) Frequency of masturbation: Less than monthly- daily Aroused easily: majority Orgasm through masturbation: majority Orgasm by intercourse: majority Change in orgasmic feeling: 58,3% |

**Sexual Wellbeing following Vaginoplasty**

| Study | Design | LoE | Sample size | Age range | Sexual topics | Methods/Tools | Outcomes regarding Sexual Wellbeing |
| --- | --- | --- | --- | --- | --- | --- | --- |
| Amend et al. 2013, Surgical reconstruction | Outcome measures | 3 | 13 AMAB | 20-54 | Intercourse, satisfaction, neoclitoral sensation, vaginal depth, orgasm | Self-developed structured interview | 23 (96%) were satisfied with neoclitoral sensitivity, which led to orgasm. Neoclitoral sensation was excellent in 18, good in 5, and unsatisfactory in 1. Eight (33 %) had engaged in intercourse, without the need for lubrication. None experienced intravaginal hair growth or loss of vaginal capacity. |
| Blanchard et al. 1983, Vaginoplasty outcome | Outcome measures | 3 | 22 AMAB |  | Orgasm, intercourse, self-reported depth adequacy, pain/discomfort during sex, discomfort after sex, frequency of sex, sexual orientation | Structured interview, pelvic exam | 19 (86,4%) had intercourse at least once: 8 experienced no pain, 2 did always, 5 did initially or after a period of sexual inactivity, 4 did slightly. 3 experienced discomfort after sex |
| Bouman et al. 1988, Sex reassignment | Outcome measures | 4 | 76 AMAB | No data | Sexual intercourse, satisfaction during sex, neo-vaginal dimensions, | Chart review | 3 had complaints due to small vaginal diameter, one was unable to perform receptive vaginal sex. 30 had intercourse with men, 11 with men, 15 had not. |
| Bouman et al. 2016, Patient-Reported | Follow-up study | 4 | 31 AMAB | 18-45 | Vaginal intercourse, neo-vaginal dimensions, sexual arousal, sexual feelings, orgasm, desire, lubrication, satisfaction | Female Sexual Function Index, Female Genital Self-Image Scale, Short Questionnaire for Self-Evaluation of Vaginoplasty, Amsterdam Hyperactive Pelvic Floor Scale—Women | 21 were sexually active, 16 had sex more than once. Every participant experienced sexual arousal. 84 % could reach orgasm, 4% could not, and 12% had not tried. |
| Brotto et al. 2005, Psychophysiological and | Prospective cross-sectional study | 3 | 15 AMAB | 21-65 | Thoughts/desire, Frequency of sexual activity, receptivity/initiation, relationship satisfaction, problems affecting sexual function, sexual arousal, nongenital physical arousal, genital arousal, pleasure from direct genital stimulation, orgasm (e.g., clitoral stimulation, intercourse, vibrator use, fantasy), satisfaction with orgasmic function, dissatisfaction or distress, effects of erotic stimuli, objective arousal: using a vaginal pulse amplitude | Self-developed questionnaire during Vaginal pulse amplitude (VPA), Brief Index of Sexual Functioning for Women (BISF-W);  Detailed Assessment of Sexual Arousal (DASA); | 4 were sexually active, 6 were able to achieve orgasm. 10 were satisfied with their orgasmic function, 3 (20%) dissatisfied or distressed. |
| Buncamper et al. 2015, Aesthetic and | Retrospective cross- sectional survey | 4 | 49 AMAB | 29-53 | Sexually activity, desire, arousal, lubrication, orgasm, satisfaction, comfort, sexual intercourse, neo-vaginal dimensions, sexual feelings | Female Sexual Function Index (FSFI), Amsterdam Hyperactive Pelvic Floor Scale-Women (AHPFS-W), Female Genital Self-Imaging Scale, short questionnaire for self-evaluation of vaginoplasty | 36 were sexually active, 27 had attempted intercourse (3 of those tried but were unable). 83.7% had reached orgasm, 10.2% had not, 6.1% had not tried. Orgasmic sensation was equal in 22.4%, less in 28.6%, more in 46.9% and was missing in 2%, compared to prior to vaginoplasty. Provoked vulvodynia was scored with a mean of 1.33 (SD 0.75; 5-point scale from never - very often). Self-reported sexual arousal was present in 44. |
| Buncamper et al. 2017, Penile Inversion | Group comparison | 4 | 100 AMAB | 18-68 | Sexually activity, desire, arousal, lubrication, orgasm, satisfaction, comfort, sexual intercourse, neo-vaginal dimensions, sexual feelings | Female Sexual Function Index (FSFI), Female Genital Self-Imaging Scale | 42 had been sexually active in the last 4 weeks. Median score for vaginal functionality: 8 (range 2-10; 1-10 scale, 10 being better; n= 45). |
| Cardoso da Silva. et al 2016, WHOQOL-100 | Follow-up study | 4 | 47 AMAB | 16-54 | Marital status, sexual activity | WHOQOL-100 | 5 were in a stable relationship, 42 were not. |
| Cocci et al. 2019, Male-to-female | Outcome measures | 4 | 94 AMAB | M 29.5 y | Intercourse, erogenous sensitivity | Not specified | 81 (86.1%) had intercourse. Erogenous sensitivity during dilatation, intercourse or masturbation was present in 78 (82.9%). |
| Collyer et al. 2002, Patient satisfaction | Outcome measures | 4 | 57 AMAB | 21-35 | Orgasm, sexual satisfaction | Self-developed questionnaire | 34 patients were more sexually satisfied post-surgery; 17 patients had no change; 4 patients were less satisfied. |
| di Summa et al 2019, Colic-based | Outcome measures | 4 | 43 AMAB | 22-69 | Satisfaction with the appearance/dimensions of the genitals, satisfaction with genital function(ing), orgasm (clitoral, vaginal or both), difficulties achieving orgasm, dyspareunia | Retrospective chart review, custom questionnaire | *Of n=28* 10 (35.7%) were very satisfied with sexual functioning, 14 (50%) satisfied, 4 (14.3%) unsatisfied, none very unsatisfied. 25 (89.3%) were satisfied or very satisfied with vulvar appearance, 3 (10.7%) unsatisfied, none very unsatisfied. |
| Djordjevic et al. 2011, Rectosigmoid vaginoplasty | Outcome measures | 4 | 27 AMAB 59 women | 18-57 | Vaginal dimensions, mucous production, sexual satisfaction, sexual activity, time till first intercourse, pain | FSFI, interview | Sexual function was rated satisfactory in 21, 6 were unsatisfied. 73 individuals of the entire cohort were sexually active, separate results not provided. 27 experienced temporary mild bleeding and dyspareunia. |
| Eldh et al. 1993, Construction of | Outcome measures | 4 | 20 AMAB | No data | Orgasm, sexual function, clitoral sensation, intercourse | Chart review | 20 could reach orgasm through masturbation or intercourse. 19(95%) were pleased with their neoclitoral sensation, 1 (5%) had no sensitivity. |
| Freundt et al. 1993, A modified | Outcome measures | 4 | 23 AMAB | 16-52 | Sexual relations, vaginal function, sexual satisfaction, intercourse | Stuctured interview, pelvic examination | 5 had regular intercourse, 4 women occasionally, 1 did not. Sexual satisfaction was rated good by 2, satisfactory by 2, doubtful by 5, and unsatisfactory by 1. 2 were satisfied with sex life (20%), 4 were neutral, and 4 dissatisfied. |
| Giraldo et al. 2004, Corona glans | Outcome measures | 4 | 16 AMAB, | 20-41 | Orgasm | Chart review | 16 were able to achieve orgasm. |
| Goddard et al. 2007, Feminizing genitoplasty | Outcome measures | 4 | 233 AFAB, 70 (follow-up) | 19-76 | Clitoral sensation, sexual arousal, vaginal dimensions, intercourse, orgasm | Telephone questionnaire | Of 70 with follow-up, 64 had a neoclitoris and 62 a vaginal canal: 14 had regular intercourse. 31 could reach clitoral orgasm. Of 183 with neoclitoral formation: neoclitoris was sensitive in 158, insensitive in 5, NA in 20. 4 experienced painful or uncomfortable clitoral sensations. |
| Hess et al. 2016, Modified preparation | Follow-up study | 4 | 96 AMAB | 19-62 | Neo-clitoral sensation, orgasm | Semi-quantitative grading of neo-clitoral sensitivity | Assessment of sensitivity by brushing over the clitoris with a brush, and pallesthesia by placing a 64 Hz tuning fork on the clitoris. A semi quantitative scoring system was formed by accumulating both: grade 0, no tactile sensation and complete pallanesthesia; grade 1, reduced pallesthesia and no tactile sensation; grade 2, intact pallesthesia and reduced tactile sensation; grade 3, complete pallesthesia and tactile sensation. n= 79: 11 had grade 1, 12 grade 2, and 56 grade 3. After second-stage (cosmetic corrections). n= 73: 59 could reach orgasm, 7 could not despite trying, 7 (9.6%) had not tried. |
| Hess et al. 2018, Sexuality after | Follow-up study | 4 | 119 AMAB | 16-68 | Sexual orientation, intercourse, frequency of sex, orgasm, orgasm frequency and sensation, satisfaction with clitoral sensitivity, satisfaction with the appearance/dimensions of the genitals, satisfaction with sex life, pleasurability of sex, sexual arousal | Unspecified questionnaire | 33.7% were heterosexual, 37.6% lesbian, and 22.8% bisexual. 67 (56.3%) did not have regular intercourse. Of those who had sexual intercourse, 55.8% rated orgasm more intense following GAS, 20.8% who felt no difference. 73.9% were satisfied with neoclitoral sensitivity, and 67.1% with vaginal depth.  Of n=88: sexual activity was always pleasurable for 31 (35.2%), sometimes pleasurable for 44 (50.0%), and never pleasurable for 13 (14.8%). |
| Imbimbo et al. 2009, A report from | Outcome measures | 4 | 163 AMAB | 21-59 | Sexual activity, type of sexual activity, orgasm, masturbation, Satisfaction with sexual life, vaginal dimensions | Telephone questionnaire | 124 were sexually active: 60 had receptive vaginal sex, 75 receptive anal sex. 32 had masturbated. Satisfaction with sexual life post-surgery was improved in 75% and worsened or unchanged in 25%. |
| Jarolim et al. 2009, Gender reassignment | Outcome measures | 4 | 129 AMAB | 18-54 | Neoclitoral erogenous sensation, orgasm, lubrication | Chart review | Of n=98: 92 (94%) had erogenous sensitivity of the neoclitoris had. 64 (65%) reached orgasm 3 mo., some with urethral secretions, which provided lubrication. |
| Kanhai et al. 2016, sensate vagina | Outcome measures | 4 | 50 AMAB | 19-65 | Erogenous sensation in both clitoral pedicles | Chart review | 46 (92%) experienced erogenous sensitivity and 41 (82 %) sexual sensations in the clitoris. 44 (88%) experienced erogenous sensitivity and 31 (62 %) sexual sensitivity of the sensate pedicled spot. |
| Jiang et al. 2018, Does depth matter | Outcome measures | 4 | 30 AMAB | 28-74 | Relationship status, orgasm, sexual activity. | Case-series | Of n=14: 4 (29%) could achieve orgasm, 3 (21%) could not. 7 (50%) were not sexually active. Of n=30: 17 (57%) were married or in a stable relationship, 13 (43%) were not. |
| Karim et al. 1991, The importance of | Outcome measures | 4 | 13 AMAB | 23-51 | Swelling and narrowing of vagina during sexual arousal | Chart review | 10 experienced vaginal swelling and narrowing during sexual arousal, none did after removal of the corpora spongiosa and cavernosa, none did after removal of the tissue. |
| Kim et al. 2003, Long-term results | Outcome measures | 4 | 28 AMAB | 22-50 | Vaginal dimensions, sexual intercourse, lubricant use, pain during intercourse, orgasm, vaginal bleeding during intercourse | Cross-sectional study | 22 (78.6%) had intercourse: 1 experienced abdominal pain and 2 vaginal bleeding during intercourse, and 19 could reach orgasm during intercourse. |
| Kim et al. 2017, Is Rectosigmoid | Outcome measures | 4 | 44 AMAB 29 vaginal agenesis 8 female pseudohermaphroditis 3 gynaecologic malignancies after radical pelvic surgery | 23-47 | Sexual intercourse, orgasm | Chart review | 79 (94%) had intercourse: 72 experienced orgasm, 2 had mild intermittent abdominal pain, 6 long-lasting abdominal pain, and 6 a small amount of vaginal bleeding after intercourse. |
| Krege et al. 2001, Male-to-female | Follow-up study | 4 | 66 AMAB | 20-57 | Sexual intercourse, problems during intercourse, recurrent bleeding after intercourse, clitoral orgasm, vaginal dimensions | Self-developed questionnaire | n=31 with follow-up: 27 (87%) could reach clitoral orgasm, 18 (58.1%) had intercourse, 8 (25.8%) had problems during intercourse (1 swelling of remnants of the corpus spongiosum; 1 problems intravaginal suture line; 2 pain during intercourse; 1 recurrent bleeding after). |
| Lawrence et al. 2003, Factors associated | Follow-up study | 4 | 232 AMAB | 19–72 | Vaginal dimensions, vaginal lubrication, vaginal discharge, sensation to touch at the vaginal opening, sensation to touch deep in the vagina, vaginal pain with penetration, vaginal erotic sensation, clitoral touch sensation, clitoral erotic sensation, clitoral pain, sexual attraction, sexual experience, arousal | Self-developed questionnaire | The number of surgical complications was negatively correlated, and functional results were positively correlated with the absence of regret regarding vaginoplasty. The amount of psychotherapy and the number of complications were negatively, and functional results were positively correlated with happiness with the results of vaginoplasty. |
| Lawrence et al. 2005, Sexuality before | Follow-up study | 4 | 232 AMAB | 19–72 | Sexual orientation, number of sexual partners, frequency of sexual activity, stable partnered relationships, sexual arousal to cross-dressing or cross-gender fantasy, frequency and characteristics of orgasm after GAS | Self-developed questionnaire | Of n=226: 76% had postsurgical sexual experiences (28% had mostly female partners, 38% mostly male, 25 % bisexual).  Of n=226: 214 (95%) were sexually active prior to surgery, 12 (5.3%) were not. 72% had mostly female partners, 8% mostly male, 15% bisexual.  Of n=227, 217 (95.6%) had masturbated: 82 (36%) almost always orgasm during masturbation, 27 (12%) > half the time, 33 (15%) did < half the time, 34 (15%) rarely, 41 (18%) never, and 10 (4%) NA.   Of n=217: orgasm prior to and after surgery was almost identical for 4 (2%), very similar for 19 (9%), somewhat similar for 53 (24%), slightly similar for 52 (24%), entirely different for 57 (26%), NA for 32 (15%) .  Of n=218: orgasm after surgery was much more pleasurable for 65 (30%), somewhat more pleasurable for 45 (21%), about as pleasurable for 35 (16%), somewhat less pleasurable for 35 (16%), much less pleasurable for 8 (2%), NA for 30 (14%).  Of n=217: 52 (24%) almost always released fluids during orgasm, 22 did > half of the time for 22 (10%), 17 (8%) did < half of the time, 29 (13%) did rarely, 40 (18%) never, and 57 (26%) NA. |
| Lawrence et al. 2006, Patient-reported complications | Follow-up study | 4 | 232 AMAB | 19–72 | Vaginal dimensions, vaginal lubrication, vaginal discharge, sensation to touch at the vaginal opening, sensation to touch deep in the vagina, vaginal pain with penetration, vaginal erotic sensation, clitoral touch sensation, clitoral erotic sensation, clitoral pain, sexual attraction, sexual experience, arousal, frequency of orgasm | Self-developed questionnaire | Mean rating on 0-10 scale, 10 being better, were: 7.8 (SD 2.4) for overall happiness with genital sexual function after GAS; 4.4 (SD 2.8) for vaginal lubrication; 7.1 (SD 2.4) for mean rating for pain with vaginal penetration.  Frequency of achieving orgasm was significantly associated with overall happiness with sexual function. Individuals who could never orgasm were significantly less happy with their sexual function than others. |
| LeBreton et al. 2017, Genital Sensory | Outcome measures | 3 | 28 AMAB | 25-60 | Genital sensitivity, overall satisfaction (patient’s satisfaction with the appearance of their genitals, sexual functioning, and clitoral sensitivity), frequency of sexual activities (masturbation, mutual masturbation, vaginal intercourse, anal stimulation, anal intercourse, and orogenital stimulation) orgasm frequency with each of these activities. | Genital sensitivity: Semmes-Weinstein monofilaments (light touch), vulvalgesiometer (pressure), vibralgic 4 device (vibration), questionnaire developed by Lothstein and Shinar, self-developed questionnaire, Derogatis Fantasy Scale. | Subjective clitoral sensation was not statistically significantly correlated with sexual satisfaction.  Detection thresholds for light touch showed the highest sensitivity on the neck, followed by the anus, abdomen, clitoris, labia minora and then the vaginal opening.   Detection thresholds for pressure showed the highest sensitivity on the neck, followed by the clitoris, anus, abdomen, labia minora and the vaginal opening.   Detection thresholds for vibration showed the highest sensitivity on the clitoris; followed by the labia minora; the neck; the abdomen; and the vaginal opening and anus.  *Frequencies of sexual activities ranged from: 0= N.A.; 1= < 1/year, 2=< 1/month; 3= 1/month; 4= 2/month; 5= 1/week; 6= several times/week; 7= 1/day; 8= >1/day*. 20 (80%) had experienced orgasm. Mean frequencies of achieving orgasm prior to and after having GAS, respectively, were 0.90 (SD 1.38) and 0.56 (SD 1.36) for masturbation, 0.50 (SD 1.47) and -0.24 (SD 1.27) for mutual masturbation, 1.00 (SD 1 .29) and -0.12 (SD 1.09) for vaginal intercourse (penetrative and receptive, respectively), -1.18 (SD 1.30) and -0.16 (SD 1.52) for receptive oral sex, -0.14 (SD 1.36) and -0.36 (SD 1.15), and -0.05 (SD 1.47) and 0.08 (SD 1.04).   Prior to GAS: mean frequency of receptive anal sex was 3.20 (SD 2.63), 2.40 (SD 2.71) for insertive vaginal sex, 2.32 (SD 2.75) for receptive oral sex, and 3.20 (SD 2.48) for receptive anal stimulation. Following GAS: mean frequency of receptive vaginal sex was 3.44 (SD 2.49), 2.04 (SD 2.54) for receptive anal sex, 3.48 (SD 2.45) for receptive oral sex, and 2.48 (SD 2.69) for receptive anal stimulation. The difference between pre- and postoperative frequency of receptive oral sex was statistically significant. |
| Lindemalm et al. 1986, Long-term | Evaluation of GAS | 4 | 13 AMAB | 27-62 | Sexual adjustment, sexual function, libido, sexual activity, orgasm, partner relations | Semi structured interview, chart review | Of n=12: 11 (92%) were sexually active prior to GAS, 1 (8%) was not. Following GAS, 10 (77%) were sexually active.   Orgasm prior to GAS: 9 (69.2%) could, 1 (7.7%) could not, unclear for 3 (23.1%). Orgasm after GAS: 6 (46.2%) could (2 with ejaculation), 6 (46.2%) could not, unclear for 1 (7.6%).  Strength of libido prior to GAS was low for 5, high for 6, NA for 2. Following GAS: low for 7 (1 of which previously high; 2 NA), moderate for 1 (previously low), high for 5. |
| Lindemalm et al. 1987, Prognostic factors | Evaluation of GAS | 4 | 13 AMAB | 27-62 | Sexual adjustment, libido, sexual activity with partner, number of partners, orgasm, object choice, partner relations | Retrospective rating of interview | The following outcomes prior to GAS were associated with fair or good overall sexual adjustment after GAS : high sexual activity with a partner, strong libido, intercourse with women, and bisexual experience. High frequency of masturbation was not associated with good adjustment. |
| Manrique et al. 2018, Gender-Confirmation | Evaluation of rectosigmoid vaginoplasty | 4 | 15 AMAB | 18-32 | Sexual function | Retrospective chart review, Female Sexual Function Index (FSFI), Female Genital Self-Image Scale (FGSIS) | One (6.7%) had by narrowing at the introitus, which required intervention. The mean Female Sexual Function Index score was 28.6 (range, 24-31). Every individual achieved normal sexual function (FSFI ≥ of 25) |
| Mate-Kole et al. 1990, A controlled study | Outcome measures | 4 | 40 AMAB (20 postop, 20 preop) | 21-53 | Sexual interest, sexual relationships | Chart review | Sexual interest during follow-up of 2 years for n=20 following GAS was unchanged for 4, 15 were more active, none were less active. Sexual interest for n=20 awaiting surgery remained unchanged for 17, 0 were more active, 3 were less active. |
| Morrison et al. 2015, Long-Term Outcomes | Outcome measures | 4 | 83 AMAB | 36-78 | Dyspareunia, need for lubricant, mucorrhea, orgasmic capacity, sexual function | Phone interview; chart review | Of n=44: 43 (98%) were able to orgasm. Of n=34: 13 (38%) experienced dyspareunia. Of n=27: 7 (26%) needed lubrication during intercourse. Average rating for satisfaction for n= 24 was 4.24 (1-5 scale). |
| Mukai et al. 2017, Vaginoplasty with | Outcome measures | 4 | 15 AMAB | M 34.2 (SD 4.0) | Intercourse, pain, vaginal dimensions | Chart review | 14 (93.3 %) had intercourse. 1 (6.7%) experienced discomfort during intercourse, because of neovaginal depth of 5-6 cm. |
| Papadopulos et al. 2020, Psychological Pathologies | Follow-up study | 4 | 47 AMAB | 18-57 | Improvement of sex life, sexual orientation, change in sexual preference | Custom questionnaire | 29 (61.7%) experienced an improvement of sex life following GAS.  Prior to surgery: 12 (25.5%) were heterosexual, 22 (46.8%) homosexual, 11 (23.4%) bisexual, and 2 (4.3%) other. Following surgery n=46, 15 (32.6) were heterosexual, 10 (21.7%) were homosexual gay, 21 (45.7%) were bisexual. |
| Papadopulos et al. 2017, Combined vaginoplasty | Follow-up study | 4 | 40 AMAB | M 38.6 (SD 12.6) | Vulvar sensitivity, vaginal dimensions | Chart review and follow-up | Most women reported normal labial and vaginal sensitivity, and strong clitoral sensitivity. |
| Perovic et al. 2000, Vaginoplasty in | Outcome measures | 4 | 89 AMAB | 18-56 | Orgasm, vaginal sensitivity, vaginal moisture, intercourse, vaginal dimensions | Interview | 73 (82%) had orgasmed, 69 (79%) were having intercourse. Presence of vaginal moisture was satisfactory for 71 (80%) and unsatisfactory for 16 (18%) |
| Raigosa et al. 2015, Male-to-Female | Outcome measures | 4 | 60 AMAB | 19–50 | Frequency and quality of intercourse, orgasm, vaginal dimensions, clitoral sensation | Interview (direct questioning during follow-up) | 52 (86%) had regular intercourse. Clitoral sensitivity was acceptable and led to orgasm for all participants. |
| Reed et al. 2015, Non-grafted Vaginal | Outcome measures | 4 | 18 AMAB | No data | Vaginal dimensions, sexual function | FSFI and clinical examination | Of n=10: FSFI domain scores (lubrication 3,7; desire 3,5; arousal 4,0; orgasm 3,9; satisfaction 3,6; pain 4,7) were ≥ mid-range. Average total score was 23.4 (r 2-36). |
| Rehman et al. 1999, The reported sex | Outcome measures | 4 | 28 AMAB | 18-44 | QoL, sexual orientation, sexual activity, type of sexual contact (oral, anal, vaginal, other), sexual satisfaction, orgasm (ability and importance), lubricant use, reasoning for sexual inactivity | Interview, self-developed questionnaire | 15 (53.6%) had intercourse, all had some degree of pain during sex and all were using some form of lubricant. 7 (25%) had masturbated. 14 (50%) reported satisfaction from sexual activities and experienced orgasm most of the time, quality and intensity of orgasms were better postoperatively. 15 (53.6%) could orgasm, 7 (25%) orgasmed infrequently and 6 (21.4%) could not orgasm. |
| Rehman et al 1999  Formation of | Outcome measures | 4 | 10 AMAB | 23-60 | Clitoral sensitivity, QoL, sexual orientation, sexual activity, type of sexual contact (oral, anal, vaginal, other), sexual satisfaction, orgasm (ability and importance), lubricant use, reasoning for sexual inactivity | Interview, self-developed questionnaire | Every individual had intercourse and reported satisfactory sexual activities.  9 (90%) could orgasm (2 experienced neoclitoral necrosis, 1 one could not achieve orgasm). Clitoris sensitivity was good with sensitivity to touch, vibration and light pressure in 8 (80%). |
| Salgado et al. 2018, Primary Sigmoid | Outcome measures | 4 | 12 AMAB | M 47 (SD 15.4) | Vaginal dimensions, reported sensation, intercourse, satisfaction with depth, odour and excessive secretions | Chart review | 5 (42%) had intercourse and reported satisfaction with vaginal depth and pleasurable sensitivity.  None experienced malodour or excessive secretions. |
| Schroder et al. 1999, New women | Outcome measures | 4 | 17 AMAB | 35-58 | Orgasm, masturbation, sexual fantasies, intercourse, relationship status, sexual orientation, sexual satisfaction, genital and breast sensitivity, arousal, sexual desire | Postoperative Male-to-Female Questionnaire (Carroll & Schroder, 1993a), New Woman's Gynecological Index (NWGI) (Schroder, 1993), Stress Inventory (Carroll, 1985), Postoperative Male-to-Female Interview (Carroll & Schroder, 1993b), vaginal plethysmography | Mean self-reported rating of sexual satisfaction was 5.4 ( 0-10 scale, 10 is better).  11 (64.7%) could orgasm through masturbation (8 with ease, 3 with difficulty): 5 achieved multiple orgasms, and 5 ejaculated. Of n=16 sexually active (approximately half had intercourse): 9 orgasmed during partnered activity (4 through penile-vaginal penetration, 3 through masturbation with a partner present). |
| Seyed-Forootan et al. 2018, Autologous Fibroblast | Group comparison/follow-up study | 4 | 24 AMAB | Fibroblast: 28 SD 4y Amnion: 32 SD 3y | Vaginal dimensions, secretions, intravaginal sensitivity, orgasm, intercourse, satisfaction with intercourse | Self-developed questionnaire, interview, clinical examination of vaginal dimensions | Neo-vaginal sensitivity and lubrication was good for everyone. 18 (75%) had sexual experiences: 93.7% of the fibroblast and 87.5% of the amnion group were satisfied with orgasm and intercourse. |
| Sigurjónsson et al. 2017, Long-Term Sensitivity | Outcome measures | 4 | 22 AMAB | 23-63 | Clitoral sensitivity, orgasm, sexual dysfunction | Semmes-Weinstein monofilaments, Bio-Thesiometer, self-developed scale | Average clitoral tactile thresholds was 12.5 g/mm2, average vibration threshold was 0.3 m. Surgical complications were not associated with diminished clitoral sensitivity or orgasmic capacity. |
| Soli et al. 2008, Male to female | Outcome measures | 4 | 15 AMAB | 21–60 | Orgasm, clitoral sensitivity | Interview, self-developed questionnaire | 7 (46.7%) experienced some form of climax during intercourse. Clitoral sensitivity was present and pleasant for every individual, and was present during digital examination by the authors. |
| Stanojevic et al. 2007, Sacrospinous ligament | Outcome measures | 4 | 62 AMAB | 18-58 | Ability to perform intercourse | Chart review | 42 (76%) had intercourse. |
| Stein et al. 1990, Follow-up observations | Follow-up study | 4 | 22 AMAB | 20-49 | Orgasm, vaginal intercourse,  pain during intercourse, need for lubricants | Interview, physical examination | 2 (14.3%) had never orgasmed, 6 (43%) seldom, 2 (14.3%) usually, NA for 4 (28.6%). Orgasm was not at all important for sexual satisfaction for 3 (21.4%), somewhat important for 6 (42.9%), very important for 1 (7.1%), NA for 4. 7 (31.8%) had intercourse: 1 (14.3%) used lubricants always, 3 (42.9%) often, 1 (14.3%) never, unknown for 2 (28.6%).  Of n=9: 6 (66.7%) had intimate lovers prior to GAS, 3 (33.3%) did not. |
| Tavakkoli Tabassi et al. 2014, Fold-back | Outcome measures | 4 | 112 AMAB | M 25.8 (SD 3.3) | Satisfaction with function | Chart review | 96 (85.7%) were satisfied with the appearance and function, 16 (14.3%) were dissatisfied (10 due to depth or stenosis, 6 due to aesthetics). |
| Thalaivirithan et al. 2018, Application of embryonic | Outcome measures | 4 | 30 AMAB | 21-42 | Satisfaction with sexual function, frequency of sexual activities (oral, anal), orgasm, sexual satisfaction | Chart review | 26 (86.6%) could orgasm, 30 (100%) had intercourse. Frequency of receptive, oral sex increased and anal sex decreased significantly following GAS.  Satisfaction with sexual function and the appearance of the labia, vulva and clitoris was good-very good for 98% (5-point scale, unsatisfactory-very good).  Sexual satisfaction was statistically (positively) correlated with vaginal function and depth, clitoral sensation, appearance of the vulva/labia minora, and natural lubrication and negatively correlated with depression scores. |
| Toolenaar et al. 1993, The occurrence of | Cross-sectional study | 4 | 11 AMAB  6 women with MRKHS | AMAB: 22-48 MRKHS: 19-28 | Intercourse, lubricant | Self-developed questionnaire, clinical examination | 14 (82.4%) had regular intercourse, 3 (17,6%) did not have a sexual partner. 15 (88,2%) made use of lubricants. 13 (76.5%) experienced white discharge, 15 (88,2%) slight blood loss (6 following intercourse, 9 spontaneously). 3 experienced vaginal cramping (1 solely following sex). |
| van der Sluis et al. 2016, Long-Term | Outcome measures | 4 | 24 AMAB | 22-73 | Intercourse, adequacy of vaginal dimensions, sexual arousal, orgasm, desire, lubrication, sexual satisfaction, discomfort | Female Sexual Function Index (FSFI), Amsterdam Hyperactive Pelvic Floor Scale for Women, (AHBBS) Female Genital Self-Imaging Scale (FGSIS), self-developed questionnaire | 8 (89%) had intercourse, 1 had never. Mean FSFI satisfaction domain score was 4.2 (SD 1.3), mean score for orgasm 4.0 (SD 2.2). 8 (89%) had performed (frequent) penetrative intercourse, orgasm was possible through direct neoclitoral stimulation.  8 (89%) had intercourse frequently, depth was adequate. 1 did not have intercourse (identified as asexual). Sexual arousal was possible, orgasm could be reached through neoclitoral stimulation.  Mean rating for neovaginal functionality was 7.3 (SD 1.8; 1-10 scale), appearance was 7.4 (SD 1.9) |
| van der Sluis et al. 2016, Morphological spectrum | Outcome measures | 4 | 26 AMAB | 19–52 | Sexual activity (type of activity), sexual preference, lubricant use, condom use, vaginal symptoms (discharge, odour, pain) | Self-developed questionnaire, clinical examination (biopsies, vaginal swabs) | 8 (31%) reported discharge, 4 (15%), 1 (4%) reported tenesmus, 4 (15%) neovaginal pain (3 of which during deep penetration). |
| Wagner et al. 2010, Male-to-female | Outcome measures | 4 | 50 AMAB | 25–52 | Satisfaction with vaginal dimensions, orgasm, intercourse, pain during sex | Self-developed questionnaire | 35 (70%) had achieved clitoral orgasm, 42 (84%) had regular intercourse (2 of which reported pain during intercourse). |
| Watanyusakul 2019, Vaginoplasty Modifications | Outcome measures | 4 | 580 AMAB | 18-65 | Vaginal depth | Not specified | Average depth > 1 year follow-up was 16.0 cm. |
| Weyers et al. 2009, Long-term assessment | Follow-up study | 3 | 50 AMAB | M43.06 (SD 10.42) | Importance of sex, sexual functioning, relationships (status and quality), sexual preference | Female Sexual Function Index (FSFI), serum hormone levels, self-developed questionnaire | 3 (6%) were not interested in sex. Median score for importance of sex in a relationship was 6 (interquartile range 5-9; 0-10 scale).  Mean FSFI total score was 16.95 (SD 10.04). Overall FSFI scores were positively correlated with sexual satisfaction, general health perception and satisfaction with female appearance as perceived by others. FSFI total scores were highest for heterosexual individuals, intermediate for bisexual and lowest for homosexual individuals. There was no correlation between estradiol levels and mode of estrogen administration with testosterone levels and FSFI scores. |
| Wu et al. 2009, Laparoscopic vaginal | Outcome measures | 4 | 11 AMAB 67 DSD women 7 Cis women | AMAB: M 23.5 (SD 3.8) DSD: M 24.7 (SD 4,6) Cis: M 47.8 (SD 4.1) | Intercourse (time between surgery and first contact), orgasm, lubrication, satisfaction with sexual life, vaginal dimensions, dyspareunia, bleeding during intercourse | Chart review, self-developed questionnaire | 71 (88.8%) was sexually active. More than half reported frequent orgasms, and 90% reported adequate lubrication for intercourse. None reported dyspareunia, use of external lubrication, or mild bleeding during intercourse. |
| Zavlin et al. 2017, Male-to-Female | Follow-up study | 4 | 49 AMAB, 40 with questionnaire results | M 38.6 (\|SD 12.6) | Intercourse (satisfaction with), (satisfaction with) orgasmic capacity, orgasm, masturbation, (satisfaction with) clitoral sensitivity, pain during masturbation or intercourse, sexual orientation | Self-developed questionnaire | Mean scores for satisfaction on 0-10 scale (10 is better) with: orgasm 8.21 (SD 2.47, n=38); preoperative intercourse 3.29 (SD 2.75, n=7); postoperative intercourse 6.7 (SD 2.03, n=23).  Mean rating of clitoral sensation was 8.53 (SD 1.93, n= 40), and erogenous clitoral sensation was 8.48 (SD 2.04, n=40). Mean rating of pain during masturbation or intercourse was 2.33 (SD 2.89; n=39; 10 is extreme pain).  7 (17.5%) engaged in regular intercourse prior to GAS, 57.5% following GAS. |
| Zavlin et al. 2019, Age-Related Differences | Cross-sectional study | 4 | 40 AMAB | 19-66 | Sexual orientation, marital status, frequency of intercourse, sexual preference, satisfaction with intercourse, sexually active, | Self-developed questionnaire | Following GAS, younger individuals were mostly attracted to men (52.6%), later-onset individuals mostly to women or both (85.7%). Younger trans individuals were more frequently sexually active (73.7% vs 42.9%). |

**Sexual Wellbeing following Breast Augmentation**

| Study | Design | LoE | Sample size | Age range | Sexual Topics | Methods/Tools | Outcomes regarding Sexual Wellbeing |
| --- | --- | --- | --- | --- | --- | --- | --- |
| Weigert et al 2013 Patient satisfaction | Follow-up study | 4 | 35 AMAB | 18,9-62,6 | Sexual wellbeing | Breast-Q | Sexual wellbeing: 4 months post-op: ↑ 34 points 12> months post-op: ↑ 33 points |

**Sexual Wellbeing following Mastectomy**

| Study | Design | LoE | Sample size | Age range | Sexual Topics | Methods/Tools | Outcomes regarding Sexual Wellbeing |
| --- | --- | --- | --- | --- | --- | --- | --- |
| Esmonde et al 2019 What is “Nonbinary” | Outcome measure | 4 | 58 AFAB | 18-48 | Sexual orientation, relationship status | Retrospective chart review | Surgery improved quality of life, sex life, and comfort in physical appearance with and without clothes. |
| Poudrier et al 2019 Assessing Quality of Life | Cross sectional study | 4 | 58 AFAB | 18-58 | Satisfaction with sex life, sexual confidence (sexually confident without a shirt, likely to remove shirt for sex), comfortable during sexual activities, sexually attractive in clothes, when unclothed | Custom questionnaire | Most respondents rated their quality of life and sexual confidence before top surgery as very low. Post-op, quality of life and sexual confidence improved significantly in all domains. |
| van de Grift et al 2016 Body Image in transmen | Follow-up study | 4 | 26 AFAB | 18-59 | Sexual orientation, sufficiency as sexual partner, pleasure of sexual activities | Appearance Schemas Inventory Revised, Body Image Quality of Life Inventory (BIQLI), Body Image Scale for Transsexuals (BIS), Multidimensional Body-Self Relations Questionnaire, Rosenberg Self-Esteem Scale, Perceived Effect of Surgery (self-developed) | Mastectomy positively influences body image. Positive evaluation of the body and decreased dysphoria during social situations --> increased quality of life and self-esteem. |

**Sexual Wellbeing following Metoidioplasty**

| Study | Design | LoE | Sample size | Age range | Sexual Topics | Methods/Tools | Outcomes regarding Sexual Wellbeing |
| --- | --- | --- | --- | --- | --- | --- | --- |
| Djordjevic et al 2013 Comparison of Two Different Methods | Group comparison / Follow-up study | 4 | 207 AFAB | 18-62 | Neo-phallic dimensions, erection of the clitoris, sensation, sexual arousal, masturbation, orgasm, sexual intercourse | Chart review | Length of neophallus ranged from 4 cm to 10 cm. Majority: pleased with the aesthetic appearance.  All: erection of the clitoris and completely preserved sensation None: problems or difficulties in sexual arousal, masturbation, or orgasms.  Patients who reported sexual intercourse with partners: length of the neophallus was inadequate for full penetration |
| Djordjevic et al 2009 Metoidioplasty as a Single | Outcome measure | 4 | 82 AFAB | 18-54 | Erection of the clitoris, sensation | Chart review | All: erection of the clitoris and completely preserved sensation |
| Djordjevic et al 2018 Novel surgical techniques in | Outcome measure | 4 | 694 AFAB | 18-62 | Erection, sexual arousal, masturbation, orgasm, neophallic sensation (tactile, erogenous), sexual intercourse | Retrospective chart review | Majority: pleased with the aesthetic appearance.  All: erection of the clitoris and completely preserved sensation None: problems or difficulties in sexual arousal, masturbation, or orgasms.  Patients who reported sexual intercourse with partners: length of the neophallus was inadequate for full penetration |
| Stojanovic et al 2017  One-Stage Gender-Confirmation Surgery | Outcome measure | 4 | 374 AFAB | 18-43 | Sexual function, quality of erection, sexual arousal, erogenous sensation | Chart review | Majority: complete satisfaction with appearance, overall complete sexual satisfaction and always experienced orgasm during masturbation. All: good quality of erection, sexual arousal, and completely preserved erogenous sensation. |
| Takamatsu et al 2009 Labial ring flap | Outcome measure | 4 | 43 AFAB | 18-33 | Sensitivity, intercourse | Chart review | One patient: intercourse with his female partner. No complaints of a reduction of erogenous sensation on the clitoral glans. |
| Van de Grift et al 2019 Transmen’s Experienced Sexuality | Cross sectional study | 4 | 38 AFAB | Average 40 (SD 10) | Arousability, sexual sensation, sexual pleasure, interest in sex, sexual initiative, orgasmic capacity/intensity, satisfaction with genital appearance, satisfaction with sexual functioning/relationships, sexual orientation, use of genitals during sexual contact, the influence of GAS on sexual outcomes | Custom questionnaires | Not specific for metaidoioplasty Majority: sexually active. Areas of improvement after surgery: use and enjoyment of both chest and genitals, arousability, sexual interest, and pleasure.  Genital GAS positively impacts transmen’s sexuality, issues with genital sensation or penetration may exist |
| Vukadinovic et al 2014 The Role of Clitoral Anatomy | Outcome measure | 4 | 97 AFAB | 18-41 | Sexual arousal, masturbation, orgasm, ability to perform penetration, sexual activity (type of activity), quality of erection, erogenous sensation, sexual satisfaction | Biographical Questionnaire for Transsexuals and Transvestites, self-developed questionnaire | Majority: complete satisfaction with appearance, overall complete sexual satisfaction and always experienced orgasm during masturbation. All: good quality of erection, sexual arousal, and completely preserved erogenous sensation. Patients who reported sexual intercourse with partners: length of the neophallus was inadequate for full penetration |

**Sexual wellbeing following Phalloplasty**

| Study | Design | LoE | Sample size | Age range | Sexual Topics | Methods/Tools | Outcomes regarding Sexual Wellbeing |
| --- | --- | --- | --- | --- | --- | --- | --- |
| Barrett et al 1998 Psychological and | Group comparison | 4 | 63 AFAB | 17-72 | Satisfaction with sexual function, satisfaction with current relationship, sexual practise | Self-developed questionnaire, semi-structured interview | Genital appearance 5 point scale: preop 1.35  postop 3.84 (95%CI1.87 - 3.11) Current relationship satisfaction preop 4.64  postop 4.45 (95%CI -0.73 - 0.34) sexual function satisfaction preop 3.00 postop 3.45 (95% CI -0.38 - 1.28) |
| Bettocchi et al 2005 Pedicled pubic | Outcome measure | 4 | 85 AFAB | 19-54 | Possibility to have penetrative sexual intercourse | Chart review | Penetrative sex: 16 without penile prosthesis 17 with prosthesis (n=8 malleable; n=9 Dynaflex) 6 lost malleable prosthesis through skin erosion |
| Djordjevic et al 2018 Novel surgical | Outcome measure | 4 | 694 AFAB | 18-62 | Erection, sexual arousal, masturbation, orgasm, neophallic sensation (tactile, erogenous), sexual intercourse | Retrospective chart review | Metaidoioplasty: None reported difficulties or problems related to sexual arousal, masturbation or orgasm  Phalloplasty: Erogenous sensation based on clitoral stimulation in all None reported problems or difficulties in sexual arousal, masturbation or orgasms Sexual intercourse with complete penetration was totally adequate in all with penile implants |
| Falcone et al 2018 Outcomes of | Outcome measure | 4 | 247 AFAB | 21-69 | Phallic sensation, sexual intercourse, orgasm, partner satisfaction | Chart review, Self-developed questionnaire | Satisfactory phallic sensation: 83%.  Penetrative sexual intercourse: 77%  Orgasm: 61%  Partner satisfaction: 60% |
| Fang et al 1999 Phalloplasty in | Outcome measure | 3 | 22 AFAB | No data | Erotic sensation of the clitoris, neophallus sensation, orgasm, masturbation, intercourse | Chart review | All preserved clitorises had erotic sensation Erotic sensation on neophallus: 8 (shaft, hypothesis: coapted forearm cutaneous nerves to ilioinguinal and iliohypogastric nerves) Orgasm by masturbation the neophallus only: 1 (pudendal nerve anastomosis) Regular sex: 9  Orgasm during intercourse: 9 Sexual performance: satisfactory 9 |
| Garaffa et al 2010 Total phallic | Outcome measure | 4 | 115 AFAB | 20-55 | Neo-phallus sensation, sexual activity | Self-developed questionnaire | Complete phallus sensation: 71.5% Sensation within neourethra: 14,7% Insensate phallus: 6% Recent surgery too early to assess 5,2% Phalluses that were lost: 2,6% |
| Garcia et al 2014 Overall satisfaction | Outcome measure | 3 | 5 AFAB | M 35.1 (SD 2.23) | Erogenous sensation, orgasm, masturbation | Interview | SP: Suprapubic Phalloplasty 10 RAP-: Radial Artery free flap Phalloplasty without nerve anastomosis 5 RAP+: Radial Artery free flap Phalloplasty with nerve anastomosis 10  Orgasm pre-op: SP: 9 of 10 RAP -: 4 of 5 RAP +: 7 of 10 Orgasm post-op SP: all RAP -: all RAP +: 8 of 10 Orgasm with direct stimulation of buried clitoris: SP: all RAP -: all RAP +: 4 of 6 masturbate with phallus SP: 9 of 10 RAP -: all RAP +: 9 of 10 diminished orgasm after penile prosthesis: none regret of surgery: none |
| Leriche et al 2008 Long-term outcome | Outcome measure | 4 | 56 AFAB | 20-44 | Cutaneous sensitivity, erogenous sensitivity, sexual satisfaction, satisfactory sexual intercourse with penetration | Self-developed questionnaire | Cutaneous sensitivity of the phalloplasty: 83%  Erogenous sensitivity: 9% Sexual satisfaction (penetration with penile implant): 51% |
| Monstrey et al 2009 Penile reconstruction | Outcome measure | 4 | 280 AFAB, 7 men with various conditions | No data | Sensitivity, improvement in sexuality, orgasm, ability to perform penetration. | Chart review | Tactile sensitivity: 100% Improvement in sexuality: 80% Sexually active: 100% Explantation rate erection prosthesis: 44% Sexual intercourse with penetration: 80% |
| Noe et al 1974 The surgical construction | Outcome measure | 4 | 12 AFAB | No data | Ability to perform penetrative sex, orgasm | Chart review | Intercourse: 10 of 12 Orgasm: 9 of 12 |
| Papadopulos et al 2001 Usefulness of free | Outcome measure | 4 | 24 AFAB | No data | Intercourse, phallic sensitivity, pain | Self-developed questionnaire, clinical and radiologic examination | Questionnaire score: 2=excellent; 1=acceptable; 0=poor Sensibility: Free forearm flap: 1,41 Free fibula flap: 0,66 Intercourse: Free forearm flap: 0,83 Free fibula flap: 2 Pain: Free forearm flap: 1,41 Free fibula flap: 1,5 |
| Ranno et al 2007 Neo-phalloplasty with | Cross-sectional study | 4 | 18 AFAB | 24-38 | Contractile power (measured and asked) | Clinical examination of phallic contraction power (measurement of weight lifted and electromyography) | "Paradox erection": 18 Neo-phallus length (relaxed): 7-17 cm (mean 12.2 cm) Circumference (relaxed): 13-20 cm (mean 13.7 cm) Contract the muscle: all  Average length reduction: 3.08 cm  Average circumference enlargement: 4 cm |
| Ranno et al 2008 | Outcome measure | 4 | 22 MtF | No data | Intercourse, penile dimensions (relaxed and contracted) | Self-developed questionnaire, clinical examination | Sexual intercourse without the need for prosthesis Successful contraction when 2cm shortened with average weight 1129gr |
| Schaff et al 2009 | Outcome measure | 4 | 37 AFAB | No data | Intercourse (and it's quality), sensation | Self-developed questionnaire, chart review | Fibula flap, sexual intercourse: Excellent: 100% Sensibility minor to forearm flap: 83,3% |
| Terrier et al 2014 Surgical outcomes | Outcome measure | 4 | 24 AFAB | 28-57 | Satisfaction with function, satisfaction with sexual life, sexual function and psychosexual well-being | Semi-structured interview, self-developed questionnaire | Satisfaction with: Sexual life: 79%  Sexual activities: 85%  Intercourse: 46%  Frequency of orgasm: 55%  Appearance of penis: 83% Length of penis: 88% Diameter of penis: 88% |
| Van de Grift et al 2019 Transmen's experienced | Cross sectional study | 4 | 38 AFAB | Average 40 (SD 10) | Arousability, sexual sensation, sexual pleasure, interest in sex, sexual initiative, orgasmic capacity/intensity, satisfaction with sexual functioning/relationships, sexual orientation, use of genitals during sexual contact, the influence of GAS on sexual outcomes | Custom questionnaires | Sexual partner: 78% Sexual attraction: mainly women Use of chest during sex: 40% Use of genitals during sex: 78% Satisfaction with sexual function: Metaidoioplasty: 63,5% Phalloplasty: 28% Arousability: ↑32%; = 65%; ↓3% Sexual sensation: ↑45%; = 39%; ↓16% Sexual pleasure: ↑40%; = 47%; ↓13% Interest in sex: ↑39%; = 45%; ↓16% Sexual initiative: ↑26%; = 58%; ↓16% Orgasmic intensity: ↑21%; = 52%; ↓23% Orgasmic capacity: ↑18%; = 52%; ↓26% |
| Vesely et al 2007 | Outcome measure | 4 | 22 AFAB | 24–38 | Ability to have sexual intercourse | Self-developed questionnaire | Sexual intercourse: 42%  Muscle movement: 100% to stiffen the penis and/or move the penis during sexual intercourse  Penetration, but too short to keep inside: 11% No sexual activity: 16% Penetration not possible: 31% (too wide, too small, or too soft) |
| Wierckx et al 2011 Quality of life and | Follow-up study | 4 | 49 AFAB | 22-54 | Relationship status, sexual preference, sexual activities (frequency, type of activity, pain), sexual wellbeing, masturbation, sexual satisfaction, orgasm, arousal | Self-developed questionnaire | Sexual orientation:  Attracted to females: 42 Bisexual: 2 Attracted to males: 5  Relationship: 31 of 49 Gender of partner:  Heterosexual woman: 24 Homo/bisexual woman: 4 Homo/bisexual man: 1 AMAB transsexual: 2  Freq sexual activities:  1-2/month: 48% Several times weekly: 30%  Sexual satisfaction: (Very) satisfied: 64%  Use of clitoris during coitus:  Touching: before: 12,8% after: 13% Stimulation: before: 34% after: 38,3%  Use of vagina during coitus:  Touching: before: 5,8% after: 11,4% Penetration: before: 25,5% after: 11,4%  Results with vs without erection prosthesis: Freq of masturbation: (More than) weekly: 64,5% vs 30,8%  Freq of easy arousal:  Half of time: 45,2 vs 53,8 Orgasm through masturbation: (Almost) always: 67,9% vs 58,3% Orgasm through coitus: (Almost) always: 38,9% vs 37,5% Change in orgasmic feeling: 64,3% vs 58,3% |
| Wierckx et al 2011 Sexual desire | Outcome measure | 3 | 45 AFAB | 22-54 | Sexual desire, frequency of sexual activity and masturbation, sexual satisfaction with the current partner | The Dutch version of the Sexual Desire Inventory | Sexual desire after GAS: (Much) higher: 32  No change: 11 Decrease: 1 Nor willing to answer: 1  Satisfaction with sexual life: 64%  Neutral 18% Unsatisfied: 18% |
| Zuckerman et al 2015 Penile prosthesis | Outcome measure | 4 | 15 AFAB 16 Cis men | M 35.6 | Sexual activity | Chart review | Sexually active post-implant: 85% |

AFAB: Assigned Female At Birth (transmasculine individual, trans man); AMAB: Assigned Male At Birth (transfeminine individual, trans woman); MRKHS: Mayer Rokitansky Kuster Hauser Syndrome; LoE: Level Of Evidence (Oxford Centre for Evidence-Based Medicine 2011);
